# Alkyl Bismuth Cations: Synthesis, Characterization, and Application as Z‐Type Ligands

**DOI:** 10.1002/chem.70803

**Published:** 2026-02-25

**Authors:** Johannes Schwarzmann, Joel Nitzsche, Cissie Slopianka, Liva Hartmann, William Augustinov, Alexandre Gagnon, Carsten von Hänisch, Crispin Lichtenberg

**Affiliations:** ^1^ Department of Inorganic Chemistry Philipps Universität Marburg Marburg Germany; ^2^ mar.quest | Marburg Center for Quantum Materials and Sustainable Technologies Marburg Germany; ^3^ Département de Chimie Université du Québec à Montréal Montréal Québec Canada

**Keywords:** alkyl bismuth cations, bismane, *cyclo*propyl, metal‐only Lewis pairs, Z‐type ligands

## Abstract

When compared to their aryl‐substituted counterparts, the chemistry of alkyl bismuth cations is still in its infancy. In order to tackle this synthetic challenge and close this gap of knowledge, we have developed protocols for the synthesis of precursors with unusual bismacyclic motifs and *cyclo*propyl ligands, Bi(CH_2_)_5_Br and Bi*c*Pr_2_Cl. These and the more common precursor Bi*i*Pr_2_Cl could be turned into the cationic species [BiR_2_(SbF_6_)] (*R*_2_ = (CH_2_)_5_, *c*Pr_2_, *i*Pr_2_), the first examples of alkyl bismuth cations with longer alkyl chains and cyclic motifs. The ability of these compounds to act as soft Z‐type ligands is demonstrated by the formation of metal‐only Lewis pairs in reactions with the platinum(0) precursor [Pt(PCy_3_)_2_]. The products of these reactions [(PCy_3_)_2_Pt→BiR_2_(SbF_6_)] show unsupported Pt→Bi bonds, and could be isolated and fully characterized. Pt oxidation and ring‐opening of the *cyclo*propyl ligand were uncovered as a decomposition pathway.

## Graphical abstract

Rare examples of alkyl bismuth cations [BiR_2_(SbF_6_)] are presented, including *cyclo*propyl ligands and a bismacyclic structure. The bismuth‐centered Lewis acids act as Z‐type ligands toward the electron‐rich platinum atom in [Pt(PCy_3_)_2_], generating a set of metal‐only Lewis pairs. The structural and spectroscopic properties of these rare types of bismuth compounds have been investigated.

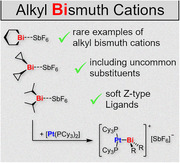

## 1 Introduction

**SCHEME 1:**
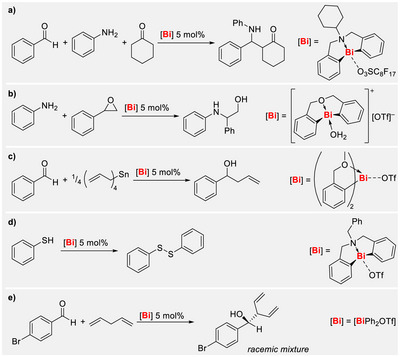
Reactions catalyzed by diarylbismuth cations: (a) Mannich‐type reaction; (b) epoxide opening; (c) allylation of aldehydes; (d) oxidation of thiophenol; and (e) allylic C─H functionalization.

Lewis acidic bismuth compounds have increasingly been in the focus of research efforts in recent years, aiming at the investigation of unusual Lewis acid/base pair formation and catalytic applications [1, 2, 3, 4, 5, 6, 7, 8, 9, 10]. For instance, simple inorganic salts such as BiBr_3_ or Bi(OTf)_3_ can serve as commercially available Lewis acid catalysts in various organic reactions and show a pronounced functional group tolerance [6, 7, 8]. A considerable Lewis acidity of organobismuth compounds can be obtained by turning them into mono‐ or dicationic derivatives [9, 10, 11, 12, 13]. Applications of these as catalysts include Mannich‐type reactions (Scheme 1a) [14, 15], ring opening of epoxides (Scheme 1b) [16], allylation of aldehydes (Scheme 1c) [17], oxidation of thiophenol (Scheme 1d) [18], or allylic C─H functionalization (Scheme 1e) [19]. In addition, some diaryl bismuth nitrates have been explored with respect to their antibacterial and cytotoxic properties [20, 21].

**FIGURE 1:**
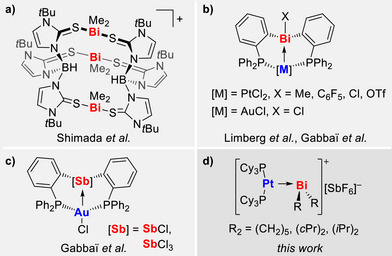
(a) Trinuclear alkyl bismuth cation. (b) Compounds with tunable bismuth‐based Z‐type ligands. (c) Compounds with tunable antimony‐based Z‐type ligands. (d) This work: variation of the alkyl group in [BiR_2_]^+^ and their use as bismuth‐based Z‐type ligands to give compounds with unsupported Pt→Bi bonds.

Currently, the literature on organobismuth cations is heavily focused on aryl ligands, most likely due to the higher stability of the triaryl bismuth precursors [2, 22, 23, 24, 25, 26, 27, 28, 29, 30] and the more challenging synthesis, storage, and handling of trialkyl bismuth compounds [31, 32, 33, 34, 35]. Nevertheless, rare examples of cationic organobismuth compounds that do not take advantage of the stabilizing effect of aryl ligands have been disclosed. For instance, the bis(allyl)bismuth cation has been isolated and utilized as an allyl transfer reagent and as an initiator for the controlled radical polymerization of activated olefins [36]. When it comes to alkyl bismuth cations, a trinuclear monocationic complex, [(BiMe_2_)_3_(Tm*^t^* ^Bu^)_2_][BiMe_2_Cl_2_] has been reported (Tm*^t^* ^Bu^ = hydro*tris*(2‐mercapto‐1‐*tert*‐butylimidazolyl)borate; Figure 1a) [37]. The dimethylbismuth cation [BiMe_2_(SbF_6_)] is the only well‐defined mononuclear alkyl‐stabilized bismuth cation to date [38]. It shows a rich coordination chemistry with various substrates, including BiMe_3_ [38], isocyanides, and phosphanes [39], as well as intriguing reactivity patterns in dynamic covalent chemistry [40]. More recently, the dimethylbismuth cation has been reported to form a Lewis acid/base pair with a platinum(0) precursor, revealing its ability to act as Z‐type ligand and to form unsupported metal→bismuth bonds [41]. In this context, peerless reactivity patterns have been reported, such as the umpolung reaction of a Pt→Bi bond by reaction with H_2_ to give a product with a Bi→Pt interaction [41].

The concept of Z‐type ligands has recently gained increased interest, as it offers a unique approach to modulate the electronic properties and reactivity of molecular coordination entities [42, 43, 44, 45, 46]. In the chemistry of heavier group 15 elements, Z‐type ligands used to be strongly focused on chelating ligands, which use phosphane functional groups to bring a transition metal atom into position for supported metal→pnictogen donor acceptor interactions (Figure 1b,c) [43, 47, 48, 49, 50, 51]. While the influence of varying substitution patterns and different pnictogen oxidation states on metal→pnictogen bonding have been investigated, the influence of bismuth‐bound substituents on the overall properties of the compounds with unsupported metal→bismuth bonds remains an open scientific question.

Here, we report the synthesis and full characterization of three alkyl bismuth cations, including uncommon bismacyclic and *cyclo*propyl structural motifs and their coordination to Pt(0) as Z‐type ligands, along with the analysis of their structural and spectroscopic properties.

## 2 Results and Discussion

While various dialkyl bismuthanes have been reported [38, 40, 52, 53, 54, 55], saturated cyclic bismuthanes remain far less explored. Early work has demonstrated the accessibility of such compounds [55, 56, 57], but crystallographic investigations and reactivity studies remain limited so far. This is likely due to the lack of well‐defined, isolable precursors that can readily be functionalized in well‐predictable transformations. Wieber and Rudolph fortuitously discovered the saturated heterocycles 1‐methylbismolane (MeBi(CH_2_)_4_) and 1‐methylbismane (MeBi(CH_2_)_5_). The bismuthalkanes Me_2_Bi(CH_2_)_n_BiMe_2_ (*n* = 4, 5), which they had initially aimed to prepare via the reaction of in situ generated Me_2_BiNa with Br(CH_2_)_n_Br (*n* = 4, 5), were found to undergo decomposition within a few days, liberating Me_3_Bi as well as either methyl–bismolane (MeBi(CH_2_)_4_) or methyl–bismane (MeBi(CH_2_)_5_). These cyclic compounds are liquid, display remarkable thermal stability, and can be isolated in pure form by removal of Me_3_Bi through distillation. To deliberately access these compounds, Wieber and Rudolph carried out the reaction of methylbismuth dibromide with the corresponding di‐Grignard reagent BrMg(CH_2_)_n_MgBr (*n* = 4, 5). However, the yields were rather low, merely amounting to about 20% [57]. The reactivity of these purely organometallic compounds toward further functionalization or bonding interaction with external substrates has not been probed to date.

In this study, we therefore set out to perform an analogous reaction with bismuth trihalides with the objective of generating the corresponding halidobismane derivatives as precursors for further reactions such as the formation of new bismuth cations.

**SCHEME 2:**
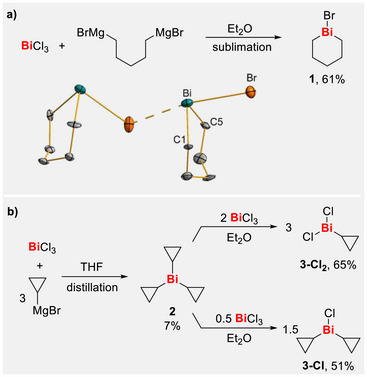
(a) Synthesis of **1** and its and molecular structure in the solid state. Displacement ellipsoids are drawn at 50% probability level; hydrogen atoms are omitted for clarity. Two out of four independent formula units are shown, equivalent to a cut‐out of the one‐dimensional coordination polymer in the solid state. Selected bond lengths [Å] and angles [°]: Bi–Br: 2.8534(19)–3.0191(18), Bi–C: 2.23(2)–2.263(16), Br–Bi–Br: 170.02(5)–173.56(6), Bi–Br–Bi: 89.79(5)–111.93(6), C–Bi–C: 92.1(7)–93.5(7). (b) Synthesis of analytically pure **2** and its chloro‐derivatives **3‐Cl** and **3‐Cl_2_**.

To obtain 1‐bromo bismane ([Bi(CH_2_)_5_Br] (**1**); Scheme 2a), the Grignard reagent BrMg(CH_2_)_5_MgBr was freshly prepared and reacted with BiCl_3_ in diethyl ether at 0°C. Since the desired product and the magnesium salts formed as by‐products exhibit similar solubilities in most organic solvents, **1** was isolated by sublimation. The reaction did not afford the expected chloro‐bismuthane, but yielded the 1‐bromo‐bismane instead, which was isolated as a yellow solid in 61% yield. This outcome is attributable to halide exchange with MgBrCl generated during the reaction. Such halide exchange reactions are common in alkyl and aryl bismuth chemistry [54, 58, 59, 60, 61]. The lower Lewis acidity of the bromo species makes the aggregation less favorable than in the chloro species [62, 63], facilitating the sublimation of the clean product **1** from the reaction mixture. Colorless crystals of [Bi(CH_2_)_5_Br] were obtained from a THF solution at −30°C (triclinic space group *P* $\bar{1}$, *Z* = 8; Scheme 2a) [64]. In the solid state, **1** forms coordination polymers in which each bismuth atom interacts with two bridging bromine atoms. This leads to the formation of an infinite helix‐shaped chain along the crystallographic *c* axis. The interatomic Bi─Br distances amount to 2.853(2)–3.019(2) Å. These values lie within the range of Bi─Br distances found in BiBr(*t*Bu)_2_ (2.7270(4)−3.3655(5)Å.) [53] and BiBr(*i*Pr)_2_ (2.8785(5)−2.9311(5) Å) [54] and are notably shorter than the sum of the van der Waals radii of bismuth and bromine (3.92 Å) [65, 66]. The six‐membered ring adopts a half‐chair conformation due to the endocyclic bismuth atom, and the C–Bi–C angles vary from 92.1(7) to 93.5(7). The Br–Bi–Br angles lie between 170.02(5)° and 173.56(6)°, which is slightly larger than the Br–Bi–Br angle in BiBr(*t*Bu)_2_ (164.56(1)°) [53] and slightly smaller than that of BiBr(*i*Pr)_2_ (176.11(3)°) [54].

A second similarly elusive bismuthane we intended to explore is tri*cyclo*propylbismuthane (**2**) because *cyclo*propyl groups are electronically situated between alkyl and aryl substituents due to the sp^2^‐like character of the carbon‐centered orbital that is involved in the formation of M─*cyclo*propyl bonds [67]. First prepared by Gagnon et al. [68, 69, 70], open questions around the synthesis of Bi(*c*Pr)_3_ remained, because two types of products were described: first, a solid obtained after work‐up under air, and second, a yellow oil that was obtained under an inert atmosphere. Both types of products showed a similar reactivity in metal‐catalyzed reactions, and the convenience of the solid being synthesized without the necessity for inert conditions during work‐up and being storable in the freezer is intriguing. However, the physical, chemical, and spectroscopic properties of pure Bi(*c*Pr)_3_ remain uncertain at this point. Conducting the synthesis and work‐up under inert conditions led to the formation of a brown oil as a crude product, from which analytically pure Bi(*c*Pr)_3_ (**2**) could be obtained by vacuum distillation, resulting in a colorless oil, albeit in very low yield (Scheme 2b). The low yield was most likely due to thermal decomposition of **2** under heating, giving bismuth black as one of the degradation products that was observed in the distillation residue. The colorless appearance of Bi(*c*Pr)_3_ after the distillation process was a first hint at its high purity, which was confirmed by NMR spectroscopy and elemental analysis. We were not able to identify the colored contaminant that was present prior to distillation, as it was not detected in the ^1^H NMR spectrum but remained soluble in 1,4‐dioxane or pentane. Starting from pure Bi(*c*Pr)_3_, one or two *cyclo*propyl groups were selectively exchanged for a chlorido ligand in comproportionation reactions with 0.5 or 2 equivalents of BiCl_3_. Bi(*c*Pr)Cl_2_ (**3‐Cl_2_**) was formed as a colorless solid [64], whereas Bi(*c*Pr)_2_Cl (**3‐Cl**) was isolated as a yellow oil. Both compounds allow further functionalization at the bismuth atom.

**SCHEME 3:**
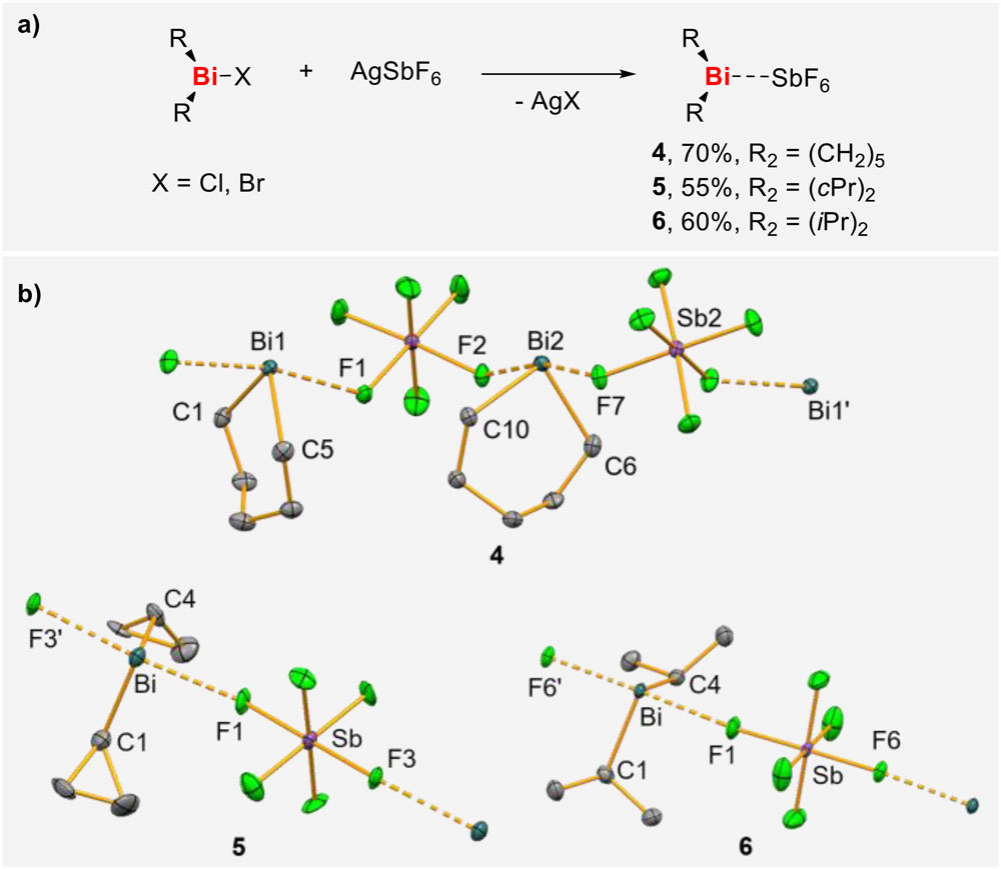
(a) Synthesis of novel bismuth cations. (b) Molecular structures of **4**, **5**, and **6**, displacement ellipsoids are drawn at 50% probability level, hydrogen atoms are omitted for clarity. Selected bond lengths [Å] and angles [°]: for **4** Bi1–C1 2.231(3), Bi1–C5 2.242(3), Bi2–C6 2.244(3), Bi1–F1 2.4675(19), Bi2–F2 2.4638(19), Bi2–F7 2.5144(19), C1–Bi1–C5 92.75(12), C6–Bi2–C10 93.40(12) C1–Bi1–F1 86.71(10), F2–Bi2–F7 166.79(7); for **5** Bi–C1 2.13(4), Bi–C4 2.21(3), Bi–F1 2.469(19), Bi–F3′ 2.514(17), C1–Bi–C4 93.2(14), C1–Bi–F1 88.6(11), F1–Bi–F3′ 170.4(6); for **6** Bi–C1 2.271(3), Bi–C4 2.273(3), Bi–F1 2.4727(18), Bi–F6′ 2.4644(18), C1–Bi–C4 91.63(11), C1–Bi–F1 93.50(9), F1–Bi–F6′ 176.17(6).

Dialkyl bismuth cations with sufficiently weakly coordinating anions remain extremely rare, with only a single representative, [BiMe_2_(SbF_6_)] (**I**), having been reported to date. Compounds **1** and **3‐Cl** as well as the literature‐known organobismuth halide [Bi(*i*Pr)_2_Cl] [54, 71, 72] were identified as promising candidates to expand this class of compounds. Indeed, reactions of these precursors with AgSbF_6_ in DCM gave the target compounds [BiR_2_(SbF_6_)] (*R* = (CH_2_)_5_ (**4**), *c*Pr (**5**) *i*Pr (**6**)), which were isolated and fully characterized (Scheme 3). Their molecular structures were confirmed by single crystal X‐ray diffraction analyses (**4**: monoclinic space group *P*2_1_/*c*, *Z* = 4, **5**: triclinic space group *P* $\bar{1}$, *Z* = 2, **6**: monoclinic space group *P*2_1_/*n*, *Z* = 4) [64]. They all form one‐dimensional coordination polymers in the solid state where the bismuth atoms are connected via fluorine atoms of the SbF_6_^–^. For **4** these fluorine atoms are in *cis* position, whereas in **5** and **6** a connection via *trans* positions is found. The Bi∙∙∙F interatomic distances amount to 2.4638(19) Å to 2.5246(19) Å (for **4**), 2.469(19) Å and 2.514(17) Å (for **5**) and 2.4727(18) Å and 2.4644(18) Å (for **6**). These values are within the range of distances found for the Bi∙∙∙F contacts in [BiMe_2_(SbF_6_)] (2.451(3) Å and 2.542(3) Å) [38] and clearly below the sum of the van‐der‐Waals radii of bismuth and fluorine (3.54 Å) [65], which suggests a relevant coordination in the solid state. Significant Bi∙∙∙F interactions were also found in DCM solution, as indicated by a broad singlet in the ^19^F NMR spectra, which is typical for SbF_6_^–^ units showing contacts to the counter cation in solution [39]. In all structures, the bismuth atom is found in a bisphenoidal coordination geometry, with the fluorine atoms of the SbF_6_^–^ units occupying the axial positions and the carbon atoms of the alkyl substituents residing in the equatorial positions. The C–Bi–F and the C–Bi–C angles range between 84.04(10)° and 93.50(9)° for all compounds, and the F–Bi–F angles are found between 166.79(7)° and 176.17(6)°, indicating the typical involvement of two p(Bi) orbitals in the formation of bonds in the equatorial plane and one p(Bi) orbital in the formation of bonds in the axial positions. The Bi–C bond lengths amount to 2.231(3) Å to 2.244(3) Å (for **4**), 2.21(3) Å and 2.13(4) Å (for **5**) and 2.273(3) Å and 2.271(3) Å (for **6**). For **4** and **5**, these values are similar to those found in [BiMe_2_(SbF_6_)] (2.215(5) Å and 2.223(5) Å). In **6**, slightly longer bond lengths were found, which is in line with the longer Bi–C bonds in the starting material Bi(*i*Pr)_2_Cl (2.312(12) Å and 2.318(12) Å), which was ascribed to higher steric bulk of the *iso*‐propyl group [72].

**FIGURE 2:**
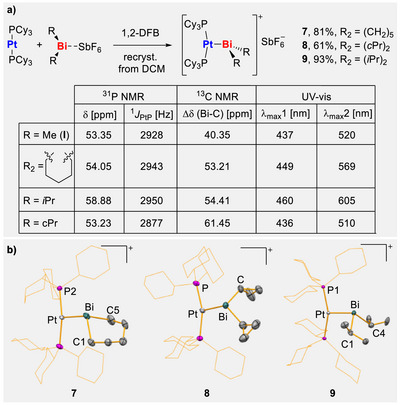
(a) Synthesis of Bi–Pt adducts **7–9**. (b) Molecular structure of compounds **7–9**, displacement ellipsoids are drawn at 50% probability level, hydrogen atoms, [SbF_6_]^–^ counterions, and lattice‐bound solvent molecules are omitted, and *cyclo*hexyl groups are shown as wireframes for clarity. For **8** this only serves as proof of connectivity. Selected bond lengths [Å] and angles [°]: for **7**: Pt–Bi 2.7190(9), Pt–P2 2.324(5), Bi–C1 2.284(6), Bi–C5 2.264(6), P1–Pt–P2 162.90(13), P2–Pt–Bi 96.68(11), C1–Bi–Pt 109.89(13), C5–Bi–Pt 105.99(16); for **9**: Pt–Bi 2.677(9), Pt–P1 2.307(2), Bi–C1 2.309(10), Bi–C4 2.307(10), P1–Pt–P2 159.09(7). P1–Pt–Bi 92.99(5), C1–Bi–C4 99.7(3), C1–Bi–Pt 106.6(2), C4–Bi–Pt 100.5(3).

In order to probe the ability of compounds **4**–**6** to act as Z‐type ligands, these cationic species were reacted with [Pt(PCy_3_)_2_] in 1,2‐difluorobenzene (1,2‐DFB). The reactions proceeded instantaneously, as indicated by a color change from yellow/orange to dark red (for **4** and **5**) or dark green (for **6**). The products of the reactions could be isolated as crystalline materials in 61%–93% yield and were identified as the targeted bismuth–platinum adducts [Pt(PCy_3_)_2_(BiR_2_)(SbF_6_)] (*R*_2_ = (CH_2_)_5_ (**7**) *c*Pr_2_ (**8**), *i*Pr_2_ (**9**); Figure 2). The corresponding ^1^H‐NMR spectra show distinct multiplets for the PCy_3_ units accompanied by characteristic signals for each ligand. A pronounced upfield‐shift of specific resonances in ^1^H and ^13^C NMR spectra is observed for the adducts **7**–**9** when compared to those of the corresponding starting materials **4**–**6**. For example, the resonances of the bismuth‐bound carbon atoms experience an upfield‐shift of 54.4–61.5 ppm in the ^13^C NMR spectra of **7**–**9**, reflecting the donation of electron density from the platinum to the bismuth center in these compounds. For compound **9**, ^1^H NMR spectroscopic analysis reveals a hindered rotation of the *i*Pr groups about the Bi–C bond, which is frozen out in ^1^H NMR spectra at 0°C in DCM solution, shows a coalescence temperature of +50°C (closed vessel), and is ascribed to steric interactions with the *cyclo*hexyl substituents (*cf*. discussion of XRD data, *vide infra*). For all cases, the ^19^F NMR spectra show a multiplet between –106 and –145 ppm, which is typical for not or weakly coordinating SbF_6_^–^ moieties [39]. ^31^P NMR spectroscopic analysis reveals signals for the PCy_3_ groups at 54.05 (^1^ *J*_PtP_ = 2943 Hz; **7**), 53.23 (^1^ *J*_PtP_ = 2877 Hz; **8**), and 58.88 ppm (^1^ *J*_PtP_ = 2950 Hz; **9**), respectively. These values are in line with those detected for previously reported [(PCy_3_)_2_Pt→BiMe_2_(SbF_6_)] (**I**) (53.35 ppm, ^1^ *J*_PtP_ = 2928 Hz) [41]. The changes in chemical shift indicate the modulation of electron density in the platinum complex fragment by variation of the organic groups in the bismuth Z‐type ligands.

The molecular structures of **7** (monoclinic space group *P*2_1_/*c*, *Z* = 4), **8** (monoclinic space group *C*2/*c*, *Z* = 8, only serves as a proof of connectivity), and **9** (monoclinic space group *P*2_1_/*c*, *Z* = 4) were determined via single crystal X‐ray diffraction analysis (Figure 2b) [64]. Each platinum atom is found in a T‐shaped coordination environment, interacting with two phosphane units and the bismuth cation. The coordination geometry around the bismuth atoms is distorted trigonal pyramidal, with primary bonding interactions between each bismuth atom and two alkyl groups as well as one platinum atom. In the case of **7**, a weak secondary interaction of the bismuth atom with a fluorine atom of the SbF_6_^–^ counter anion can be assigned based on distance criteria (Bi···F, 3.334(6) Å (not shown in Figure 2b); sum of the van‐der‐Waals radii Bi/F, 3.54 Å) [65]. However, this Bi···F contact seems not to persist in DCM solution, as determined by ^19^F NMR spectroscopy (*vide supra*). The bismuth–platinum bond lengths amount to 2.7190(9) Å (**7**) and 2.6779(5) Å (**9**), which is close to the value found for **I** (2.6867(2) Å) [41]. A marginal to moderate increase of the Pt–P bond lengths is found for **7** (2.307(2) Å) and **9** (2.324(5) Å), when compared to **I** (2.2898(11) Å and 2.2984(11) Å), which is likely due to the more pronounced +I effect and the higher steric bulk of the alkyl ligands in **7** and **9**. The steric influence of the substituents is more clearly visible in the largest torsion angle between the Pt–P bond and one Bi–C bond. For **I** this parameter shows a value of 164.7(2)° and increases slightly in the case of **7** (173.3(2)°) since the difference in steric demand is only minor. In **9** the relevant P–Pt–Bi–C torsion angle is greatly decreased to 133.0(2)°. Steric interactions between the bismuth‐bound substituents and the *cyclo*hexyl groups can be derived from the shortest H–H distances. These amount to 2.3831 Å (in **I**) but are shortened to 2.1938 Å (in **7**) and 2.1235 Å (in **9**). The Bi–C bond lengths increase from **I** (2.269(5) Å and 2.236(5) Å) over **7** (2.264(6) Å and 2.284(6) Å) to **9** (2.307(10) Å and 2.309(10) Å). This is in line with the increase in bond lengths for the respective cations (2.215(5) Å and 2.223(5) Å for [BiMe_2_(SbF_6_)], 2.237(3) Å and 2.244(3) Å for **4**, 2.273(3) Å and 2.271(3) A for **6**) [38, 41].

**FIGURE 3:**
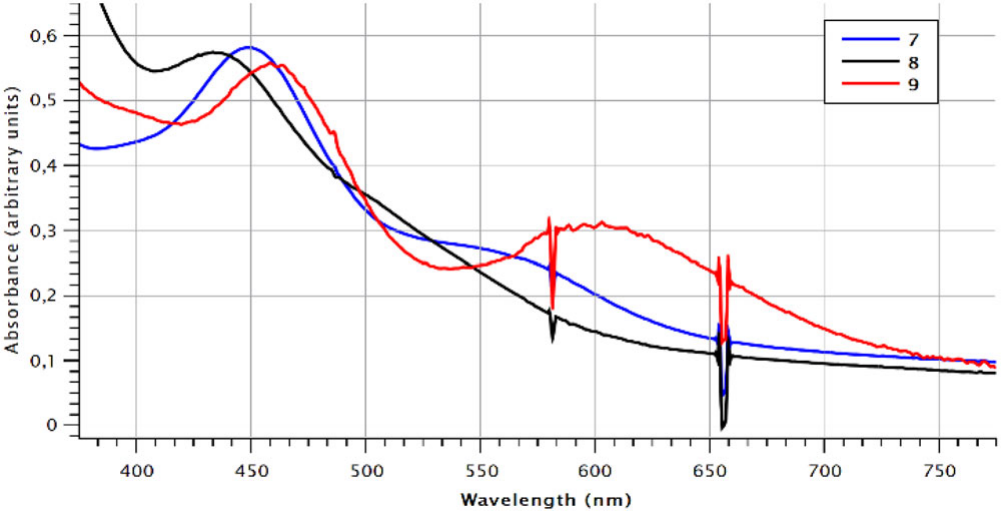
Stacked display of the UV–vis spectra of 7 (blue), 8 (black), and 9 (red) between 375 and 775 nm. The sharp dips at 580 and 655 nm are due to a lamp‐switch within the instrument.

The intensely colored compounds **7**–**9** were further investigated by UV–vis spectroscopy (Figure 3). The spectra of these structurally related compounds show a similar overall appearance, with one absorption band around 450 nm (449 for **7**, 436 for **8**, and 460 nm for **9**) and a second absorption at around 550 nm (569 for **7**, 510 for **8**, and 605 nm for **9**) that is only seen as a shoulder for compound **8**. This compares well to the spectroscopic data of the parent compound **I** (*λ*_max_ = 437 nm, 527 nm) [41]. The observed trend is in congruency with previous theoretical investigations of the absorption processes in a two‐component relativistic approach, which associated the two absorption bands with the depletion of electron density in the Bi–C *σ*‐bonds in the electron density difference plots (Supporting Information) [41].

DFT calculations on the B3LYP/def2TZVP level of theory with dispersion corrections were performed to analyze the cations **7^+^**–**9^+^** in more detail. In all cases, the Pt→Bi bond is strongly polarized toward Pt according to natural bond orbital (NBO) analysis, with Pt and Bi atomic orbitals contributing ca. 70% and 30%, respectively, to the creation of the bond‐forming orbitals. Major contributors are the s‐ and d‐orbitals of each Pt atom and one p‐orbital of each Bi atom (Supporting Information). The variation of the alkyl substituents in **7^+^**–**9^+^** results in a slight modulation of the HOMO/LUMO gap, ranging between 3.02 (**9^+^**), 3.27 (**7^+^**), and 3.38 eV (**8^+^**). This originates from differences in the LUMO energies of the Lewis acidic building blocks [BiR_2_]^+^ (*R*_2_ = (CH_2_)_5_, *c*Pr_2_, *i*Pr_2_) that vary between –8.55 for [Bi(*c*Pr)_2_]^+^, –8.75 for [Bi(*i*Pr)_2_]^+^, and –9.23 eV for [Bi(CH_2_)_5_]^+^. These are higher than the LUMO energy of the bismuth complex fragment [BiMe_2_]^+^ (–9.63 eV), which grants access to the literature‐known compound **I**. This is also reflected in the Gibbs energies associated with the reactions Pt(PCy_3_)_2_ + [BiR_2_]^+^ → [Pt(PCy_3_)_2_(BiR_2_)]^+^, which is most exergonic for the formation of **I^+^** [*R*_2_ = (CH_2_)_5_ (**7^+^**: –70.6 kcal mol^−1^), *c*Pr_2_, (**8^+^**: –64.3 kcal mol^−1^), *i*Pr_2_ (**9^+^**: –66.5 kcal mol^−1^), Me_2_ (**I^+^**: –73.9 kcal mol^−1^)] [73].

**SCHEME 4:**
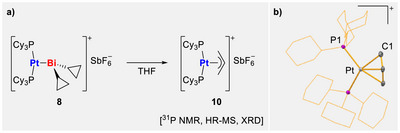
(a) Decomposition of **8** to give structurally authenticated **10**. (b) Molecular structure of **10**, displacement ellipsoids are drawn at 50% probability level, hydrogen atoms, [SbF_6_]^–^ counterion and lattice‐bound solvent molecules are omitted and *cyclo*hexyl groups shown as wireframe for clarity. Selected bond lengths [Å] and angles [°]: Pt–P1 2.3289(6), Pt–P2 2.3216(6), Pt–C1 2.199(3), Pt–C2 2.187(4), Pt–C3 2.207(2), P1–Pt–P2 111.00(2), P1–Pt–C1 91.34(7), P1–Pt–C2 120.92(12), P1–Pt–C3 157.64(7), P2–Pt–C3 91.29(7), C1–Pt–C3 66.55(10).

In agreement with the theoretical analyses, the Pt–Bi metal‐only Lewis pairs (MOLPs) **7**–**9** remain intact in weakly coordinating solvents such as dichloromethane and 1,2‐difluorobenzene. In addition, they can also be handled in a typical *σ*‐donor ligand such as THF for a given time. The time until first signs of degradation in THF were detected by NMR spectroscopy [74] ranges from ca. 20 min (in the case of **8**) to 5 d (in the case of **7**), while no signs of decomposition were noted for **I** in THF [41]. For compounds **7** and **9**, their more labile nature in polar media (compared to **I**) is ascribed to the more pronounced +I effect of the alkyl ligands, and the decomposition proceeds unselectively according to ^31^P NMR spectroscopic reaction monitoring. Compound **8** quantitatively decomposes within three days in THF. At early stages of this reaction (up to ca. 50% conversion), the selective formation of one new phosphorus‐containing product is observed according to ^31^P NMR spectroscopic reaction monitoring (*δ* = 27.26 ppm (^1^ *J*_PtP_ = 3796 Hz), which subsequently undergoes unselective degradation. The intermediate in the decomposition process could be identified through single crystal X‐ray diffraction analysis and high‐resolution mass spectrometry as [Pt(PCy_3_)_2_(η^3^‐C_3_H_5_)(SbF_6_)] (**10**, monoclinic space group *P*2_1_/*n*, *Z* = 4, Scheme 4, for detailed structural discussion, see Supporting Information) [64]. When rationalizing the sequence of reactions leading to **10**, it must be kept in mind that reactions of Pt(PCy_3_)_2_ with [BiR_2_(SbF_6_)] (R = aryl) have recently been shown to result in oxidative aryl transfer from Bi to Pt to give [PtR(PCy_3_)_2_(SbF_6_)] [75]. It can be anticipated that the *cyclo*propyl substituent in **8** contributes through an sp^2^‐like orbital to Bi–C bonding [67], thereby resembling the bonding situation of a Bi–C^aryl^ interaction. We therefore suggest the intermediate formation of [Pt*c*Pr(PCy_3_)_2_(SbF_6_)] in an oxidative *cyclo*propyl transfer from Bi to Pt, which subsequently undergoes ring‐opening of the *cyclo*propyl ligand in the coordination sphere of platinum. Similar complexes [76, 77, 78, 79] and ring opening reactions with various Pt complexes are known to the literature, including the formation of analogous *η* ^3^‐allyl complexes, when there is a weakly coordinating anion present as a second substituent [77, 78, 79]. These findings support the character of *cyclo*propyl‐substituted bismuth complexes as intermediate between alkyl‐ and aryl‐substituted bismuth compounds.

## 3 Conclusion

In conclusion, protocols for the synthesis and isolation of the unusual dialkyl bismuth monohalide precursors [Bi(CH_2_)_5_Br] and [Bi*c*Pr_2_Cl] have been presented. The latter was obtained from a modified literature procedure that grants access to analytically pure [Bi(*c*Pr)_3_]. The corresponding cations [Bi(*c*Pr)_2_(SbF_6_)] and [Bi(CH_2_)_5_(SbF_6_)], as well as [Bi(*i*Pr)_2_(SbF_6_)] have been isolated and fully characterized, representing rare cases of dialkyl bismuth monocations. When combined with the Lewis‐basic [Pt(PCy_3_)_2_], the monocationic species form the MOLPs [Pt(PCy_3_)_2_(BiR_2_)(SbF_6_)] (*R*_2_ = (CH_2_)_5_, *c*Pr_2_, *i*Pr_2_), featuring unsupported Pt→Bi donor/acceptor interactions. The bismuth complex fragments [BiR_2_]^+^ in these compounds act as Z‐type ligands and allow for the tunability of their electronic properties, as evidenced by NMR and UV–vis spectroscopic analyses. Subtle differences in the substitution pattern at the bismuth atom can considerably influence the stability and reactivity of the Bi/Pt MOLPs, as indicated by the sequence of oxidative Bi‐to‐Pt‐*c*Pr‐transfer followed by ring‐opening of the *cyclo*‐propyl derivative leading to an  *η* ^3^‐allyl‐Pt^II^ complex. It is anticipated that these findings will aid the design of future bismuth‐based Z‐type ligands as well as the modulation of electronic properties and reactivity patterns of resulting complexes.

## Conflicts of Interest

The authors declare no conflicts of interest.

## Supporting information




**Supplementary File 1**: Details of synthetic procedures and analytical data are provided in the Supporting Information. The authors have cited additional references within the Supporting Information [80, 81, 82, 83, 84, 85, 86, 87, 88, 89, 90, 91, 92, 93, 94, 95, 96, 97, 98, 99, 100].
