# Supplementary material for: Alkyl Bismuth Cations: Synthesis, Characterization, and Application as Z‐Type Ligands
Source: Chemistry. 2026 Feb 25;32(18):e70803. doi: 10.1002/chem.70803 (PMC13174907; doi:10.1002/chem.70803)
Supplement: Supplementary file 1 — Details of synthetic procedures and analytical data are provided in the Supporting Information. The authors have cited additional references within the Supporting Information [80, 81, 82, 83, 84, 85, 86, 87, 88, 89, 90, 91, 92, 93, 94, 95, 96, 97, 98, 99, 100]. [file CHEM-32-e70803-s001.pdf]

Table of Contents

1. **Experimental** 2
   1.1. **General Considerations** 2
   1.2. **Preparation of [Bi(CH_2)$_5$Br] (1)** 3
   1.3. **Preparation of [(Bi(CH_2)$_5$)(SbF_6)] (4)** 3
   1.4. **Preparation of [Pt(PCy_3)$_2$(Bi(CH_2)$_5$)(SbF_6)] (7)** 4
   1.5. **Preparation of [Bi(*c*Pr)$_3$] (2)** 4
   1.6. **Preparation of [Bi(*c*Pr)Cl_2] (3-Cl_2)** 5
   1.7. **Preparation of [Bi(*c*Pr)$_2$Cl] (3-Cl)** 6
   1.8. **Preparation of [Bi(*c*Pr)$_2$(SbF_6)] (5)** 6
   1.9. **Preparation of [Pt(PCy_3)$_2$Bi(*c*Pr_2)(SbF_6)] (8)** 7
   1.10. **Preparation of [Bi(*i*Pr)$_2$(SbF_6)] (6)** 8
   1.11. **Preparation of [Pt(PCy_3)$_2$Bi(*i*Pr_2)(SbF_6)] (9)** 8
2. **NMR Spectra** 10
3. **UV-Vis spectra** 26
4. **High Resolution Mass Spectra** 28
5. **IR Spectra** 34
6. **Structural characterization of $BiC_5H_{10}Br$ (1)** 39
7. **Structural characterization of [Bi(*c*Pr)Cl_2]** 40
8. **Structural characterization of [Pt(PCy_3)$_2$(η^3-C_3H_5)][SbF_6] (10)** 42
9. **Single-crystal X-ray diffraction** 43
10. **DFT calculations** 48
11. **References** 69

1. Experimental

1.1. General Considerations

All Experiments were, if not stated otherwise, conducted under an atmosphere of dry argon using Schlenk- and glovebox techniques. All purchased reagents were used as received, unless otherwise stated. $BiCl_3$ was sublimed prior to use. Solvents were degassed and purified according to standard laboratory procedures. NMR spectra were recorded on Bruker Avance spectrometers operating at 300 or 500 MHz with respect to ^{1}H. ^{1}H- and ^{13}C NMR chemical shifts are reported relative to $SiMe_4$ using the residual signal of the deuterated solvent as a secondary standard.[80] ^{19}F- and ^{31}P NMR chemical shifts are reported relative to $CFCl_3$ or 85% aqueous H_3PO_4 respectively as external standards. UV-vis spectra were recorded on an Analytik Jena Specord S600, mass spectrometry was conducted on a JEOL AccuTOF-GCv using LIFDI as an ionization method or a Thermo Fischer Scientific Orbitrap Q Exactive Plus using CI, ESI or LIFDI as an ionization method. Samples ionized by ESI were infused into the mass spectrometer under inert atmosphere through a syringe pump. Elemental analyses (C, H, N) were performed on a vario MICRO cube. IR spectra were recorded in an MBraun Labstar glovebox using a Bruker Alpha FT-IR spectrometer at room temperature. The spectra were evaluated with the software Opus 7.2.139_Wizard. The samples were measured by first recording a background spectrum, then placing a small amount of the sample onto the ATR crystal and recording a second spectrum. For band characterization, the following abbreviations were used: s = strong, m = medium, w = weak, vw = very weak, and br = broad. Single-crystals suitable for X-ray diffraction were coated with polyisobutylene or perfluorinated polyether oil in a glovebox, transferred to a nylon loop and then transferred to the goniometer of a Bruker D8 Quest or D8 Venture diffractometer equipped with a molybdenum (λ = 0.71073 Å) X-Ray tube or a Stoe StadiVari diffractometer equipped with a copper (λ = 1.5406) Å) X-Ray tube. Using Olex2[81], the structure was solved with the XT[82] structure solution program using Intrinsic Phasing and refined with the XL refinement package using Least Squares minimization.[83] All non-hydrogen atoms were refined anisotropically. Hydrogen atoms were included in structure factors calculations. All hydrogen atoms were assigned to idealized geometric positions. Deposition numbers 2505662 (**1**), 2505663 (**3-Cl$_2$**), 2505664 (**4**), 2505665(**5**), 2505666 (**6**), 2505667 (**7**), 2505668 (**9**), and 2505669 (**10**) contain the supplementary crystallographic data for this paper. These data are provided free of charge by the joint Cambridge Crystallographic Data Centre and Fachinformationszentrum Karlsruhe Access Structures service www.ccdc.cam.ac.uk/structures.

$[Pt(PCy_3)_2]$ was synthesized according to a literature procedure.[84]

1.2. Preparation of [Bi(CH$_2$)$_5$Br] (1)

Dried magnesium turnings (3.50 g, 144.0 mmol, 2.3 eq.) and 1,2-dibromoethane (2.71 g, 14.4 mmol 0.10 eq.) were charged in a three-necked flask together with 100 mL diethyl ether. The mixture was stirred at 40 °C while a solution of 1,5-dibromobutane (14.39 g, 62.62 mmol, 1.00 eq.) in diethyl ether (25 mL) was added dropwise over a period of 30 min. The mixture was then refluxed for 1 h. The two-phase mixture was allowed to settle for 30 min and the bottom layer containing the Grignard reagent was transferred into a dropping funnel by cannulation. The Grignard reagent was slowly added to a diethyl ether solution of BiCl$_3$ (19.75 g, 62.62 mmol, 1.00 eq.) at 0 °C within two hours. The reaction mixture was allowed to warm to room temperature and stirred overnight. After removal of volatile components under reduced pressure a solid yellow residue of **1** and MgBrCl was formed. Analytically clean **1** was obtained through sublimation at 90 °C and $1 \cdot 10^{-3}$ mbar for four days (13.49 g, 37.57 mmol, 60%).

^{1}H NMR (500 MHz, CDCl$_3$) δ = 1.64-1.71 (m, 2 H, C*H*$_2$-3), 2.08-2.12 (m, 4 H, C*H*$_2$-1,5), 3.38-3.47 (m, 4 H, C*H*$_2$-2,4) ppm.

^{13}C{^{1}H} NMR (126 MHz, CDCl$_3$): δ = 22.8 (s, *C*H$_2$-2,4), 36.9 (s, *C*H$_2$-3), 40.4 (s, *C*H$_2$-1,5) ppm.

Elemental analysis: calculated for [BiC$_5$H$_{10}$Br] (359.02 g/mol): C 16.73, H 2.81, found: C 16.59, H 2.79.

HRMS (CI): calculated for [BiC$_5$H$_{10}$]$^+$ (279.11 g/mol): m/z = 279.05863, found: m/z = 279.05850.
IR (cm^{-1}): 2957 (w), 2914 (w), 2879 (m), 2834 (s), 1437 (m), 1333 (m), 1272 (m), 1215 (s), 1185 (m), 1135 (m), 1110 (m), 1072 (w), 1024 (w), 963 (m), 912 (s), 853 (m), 779 (w), 725 (s), 678 (m), 476 (m), 459 (m), 414 (w).

1.3. Preparation of [(Bi(CH$_2$)$_5$)(SbF$_6$)] (4)

[Bi(CH$_2$)$_5$Br] (**1**) (50 mg, 0.139 mmol, 1.0 eq.) was suspended in dichloromethane (3 mL) and added to a solution of AgSbF$_6$ (48 mg, 0.139 mmol, 1.0 eq.) in dichloromethane (2 mL). Upon addition the solution turns bright orange, and a colorless precipitate is formed. After filtration the solution is layered with *n*-pentane (5 mL) and stored at –30 °C for three days. Yellow crystals of [Bi(CH$_2$)$_5$(SbF$_6$)] formed and were isolated by filtration and drying *in vacuo* (50 mg, 0.097 mmol, 70%)

^{1}H NMR (500 MHz, CD$_2$Cl$_2$): δ = 1.90 (pent, $^3J_{HH}$ = 5.58 Hz, 2 H, C*H*$_2$-3), 2.63 (t, $^3J_{HH}$ = 6.48 Hz, 4 H, C*H*$_2$-1), 5.08 (br, s, 4 H C*H*$_2$-2) ppm.

^{13}C{^{1}H} NMR (126 MHz, CD$_2$Cl$_2$): δ = 21.07 (s, *C*H$_2$-2), 40.66 (s, *C*H$_2$-3), 81.74 (s, *C*H$_2$-1) ppm.

^{19}F{^{1}H} NMR (283 MHz, CD$_2$Cl$_2$): δ = –127.8 (br, s, Sb*F*$_6$) ppm.

Elemental analysis: calculated for [C$_5$H$_{10}$BiSbF$_6$] (514.87 g/mol): C 11.66, H 1.96; found: C 11.64, H 1.87.

IR (cm^{-1}): 2928 (w), 2853 (w), 1448 (w), 1346 (w), 1223 (m), 916 (w), 837 (w), 670 (s), 635 (m), 577 (s).

1.4. Preparation of [Pt(PCy_3)$_2$(Bi(CH_2)$_5$)(SbF_6)] (7)

[Bi(CH_2)$_5$(SbF_6)] (**4**) (25 mg, 0.049 mmol, 1.0 eq.) was dissolved in 1,2-difluorobenzene (1 mL) and added to a solution of Pt(PCy_3)$_2$ (37 mg, 0.049 mmol, 1.0 eq.) in 1,2-difluorobenzene (1 mL). Upon addition the solution turns dark red. Layering the solution with *n*-pentane and storing at –30 °C gives dark red crystals of [Pt(PCy_3)$_2$(Bi(CH_2)$_5$)(SbF_6)] that were isolated by filtration and drying *in vacuo* (50 mg, 0.039 mmol, 81%). Remaining DFB can be removed by recrystallisation from DCM.

^{1}H NMR (500 MHz, CD_2Cl_2) δ = 1.24-1.39 (br m, 18 H: axial or equatorial 6 H of 4-PCy_3 and axial or equatorial 12 H of 2,6-PCy_3), 1.40-1.49 (m, 1 H, axial or equatorial Bi(CH_2)$_5$-3), 1.53-1.66 (m, 14 H: axial or equatorial 2 H of Bi(CH_2)$_5$-1 and axial or equatorial 12 H of 3,5-PCy_3), 1.77-1.85 (m, 6 H, axial or equatorial half of 4-PCy_3), 1.85-1.88 (m, 1 H, axial or equatorial Bi(CH_2)$_5$-3), 1.89-1.97 (m, 12 H, axial or equatorial 2,6-PCy_3), 1.99-2.07 (m, 12 H, axial or equatorial 3,5-PCy_3), 2.07-2.12 (br m, 8 H: axial or equatorial 2 H of Bi(CH_2)$_5$-2 and 6 H of 1-PCy_3), 3.50-3.62 (m, 2 H, axial or equatorial Bi(CH_2)$_5$-2), 4.01-4.11 (br m, 2 H, axial or equatorial Bi(CH_2)$_5$-1) ppm.

^{13}C{^{1}H} NMR (126 MHz, CD_2Cl_2) δ = 26.55 (s, 4-PCy_3), 28.03 (t, $^2J_{PC}$ = 5.95 Hz, 2,6-PCy_3), 28.53 (s, Bi(*C*H_2)$_5$-1), 31.82 (s, 3,5-PCy_3), 34.22 (s, Bi(*C*H_2)$_5$-3), 36.44 (t, $^1J_{PC}$ = 13.46 Hz, 1-PCy_3), 41.05 (s, Bi(*C*H_2)$_5$-2) ppm.

^{19}F{^{1}H} NMR (283 MHz, CD_2Cl_2): δ = –106 to –145 (br, m, SbF_6) ppm.

^{31}P{^{1}H} NMR (122 MHz, CD_2Cl_2): δ = 54.1 (s, $^1J_{PPt}$ = 2943 Hz) ppm.

Elemental analysis: calculated for [$C_{41}H_{76}BiF_6P_2PtSb$] (1270.82 g/mol): C 38.75, H 6.03; found: C 38.96, H 6.15.

IR (cm^{-1}): 2918 (m).2855 (m), 1443 (m), 1351 (m), 1175 (w), 1107 (m), 1004 (w), 960 (w), 892 (w), 849 (w), 652 (s), 510 (m), 469 (w).

1.5. Preparation of [Bi(*c*Pr)$_3$] (2)

Magnesium turnings (3.9 g, 0.16 mol, 4.2 eq.) were suspended in diethylether (75 mL) and activated by addition of one grain of previously sublimed iodine. *cyclo*Propylbromide (1 mL) was added neat to start the Grignard-reaction. Subsequently, a solution *cyclo*propylbromide (11 mL) in diethylether (45 mL) was added (overall 12 mL, 0.14 mol, 3.7 eq.), while the reaction mixture was carefully stirred and occasionally cooled with an ice bath. After the addition was completed, the reaction mixture was stirred for 15 min at room temperature and 90 min at 50 °C. The resulting Grignard solution was filtered and used without further purification.

$BiCl_3$ (12.0 g, 38.1 mmol, 1.0 eq.) was dissolved in Et_2O (50 mL), cooled to –10 °C, and the Grignard solution was added dropwise, while stirring the reaction mixture with a mechanical stirrer. After complete addition, the solvent was evaporated *via* condensation into a liquid-N_2-cooled flask at ambient pressure and heating the reaction flask to 40 °C. The remaining oil was distilled under reduced pressure ($3x10^{-3}$ mbar) at 100 °C, resulting in a colorless oil of low viscosity (800 mg, 2.41 mmol, 7%).

^{1}H NMR (500 MHz, $CDCl_3$): δ = 0.80-0.84 (m, 6 H, 2,3-*c*Pr), 1.05-1.10 (m, 6 H, 2,3-*c*Pr), 1.16-1.24 (m, 3 H, 1-*c*Pr) ppm.

^{1}H NMR (300 MHz, C_6D_6): δ = 0.80-0.88 (m, 6 H, 2,3-*c*Pr), 1.00-1.08 (m, 6 H, 2,3-*c*Pr), 1.09-1.19 (m, 3 H, 1-*c*Pr) ppm.

^{1}H NMR (300 MHz, CD_2Cl_2): δ = 0.77-0.88 (m, 6 H, 2,3-*c*Pr), 1.02-1.12 (m, 6 H, 2,3-*c*Pr), 1.14-1.29 (m, 3 H, 1-*c*Pr) ppm.

^{1}H NMR (300 MHz, $(CD_3)_2SO$): δ = 0.75-0.85 (m, 6 H, 2,3-*c*Pr), 0.99-1.10 (m, 6 H, 2,3-*c*Pr), 1.11-1.21 (m, 3 H, 1-*c*Pr) ppm.

^{13}C{^{1}H} NMR (126 MHz, $CDCl_3$): δ = 2.64 (s, CH_2, 2,3-*c*Pr), 6.47 (br s, CH, 1-*c*Pr) ppm.

Elemental analysis: calculated for [$C_9H_{15}Bi$] (332.20 g/mol): C 32.54, H 4.55; found: C 32.92, H 4.484.

IR (cm^{-1}): 3056 (m), 2982 (s), 2855 (w), 1453 (w), 1431 (m), 1218 (s), 1182 (s), 1096 (w). 1047 (m), 1020 (s), 979 (w), 856 (s), 806 (s), 734 (w), 652 (m), 437 (s).

The data in dmso-d_6 especially shows the difference to the solid Bi(*c*Pr)$_3$, published by Gagnon *et al.* where the corresponding resonances are observed at 1.22 (6 H), 1.57 (6 H) and 1.91 (3 H) in the same solvent. Since the group obtained proof for the formation of Bi(*c*Pr)$_3$ in HR-MS spectra, but the elemental analysis differs by up to 13% from calculated values, we suggest that the work-up under air may lead to (partial) oxidation of the desired compound.[68,69]

1.6. Preparation of [Bi(*c*Pr)Cl$_2$] (3-Cl$_2$)

$BiCl_3$ (0.50 g, 1.5 mmol, 2.0 eq.) was dissolved in diethylether (10 mL) and cooled to 0 °C. Bi(*c*Pr)$_3$ (**2**) (0,24 g, 0.75 mmol, 1.0 eq.) was diluted in diethylether (10 mL) and added in small portions to the $BiCl_3$-solution. The reaction was stirred at 0 °C for 30 min and for 18 h at room temperature, during that time a brown precipitate was formed. The solution was filtered and all volatiles were removed *in vacuo* to yield [Bi(*c*Pr)Cl$_2$] as a colorless solid (0.52 g, 0.16 mmol, 65%)

^{1}H NMR (500 MHz, CD_3CN): δ = 2.27 (m, 2 H, 2,3-*c*Pr), 3.29 (m, 2 H, 2,3-*c*Pr), 3.63 (m, 1 H, 1-*c*Pr) ppm.

^{13}C{^{1}H} NMR (126 MHz, CD_3CN): δ = 4.72 (s, *C*H_2, 2,3-*c*Pr), 90.51 (br s, *C*H, 1-*c*Pr, detected *via* ^{1}H-^{13}C-HSQC Experiment) ppm.

Elemental analysis: calculated for [$C_3H_5BiCl_2$] (320.95 g/mol): C 11.23, H 1.57; found: C 11.63, H 1.991.

IR (cm^{-1}): 2960 (w), 1425 (w), 1258 (s), 1181 (m), 1014 (s), 847 (w), 792 (s), 439 (m).

1.7. Preparation of [Bi(*c*Pr)₂Cl] (3-Cl)

$BiCl_3$ (237 mg, 0.75 mmol, 1.0 eq.) was dissolved in diethylether (10 mL) and a solution of Bi(*c*Pr)$_3$ (500 mg, 1.51 mmol, 2.0 eq.) in diethylether (10 mL) was added at 0 °C. The reaction mixture was stirred at 0 °C for 5 h. The solvent was removed under reduced pressure while keeping the reaction vessel at 0 °C. The remaining highly viscous oil was transferred to a glovebox, diluted with dichloromethane and filtered. The yellow solution was stored in the freezer (–30 °C) and the content of dichloromethane was determined *via* ^{1}H NMR spectroscopy (1.26 g containing 9 eq. DCM, 1.15 mmol, 51%).

^{1}H NMR (500 MHz, $CDCl_3$) δ = 1.73 (dt, $^3J_{HH}$ = 5.24 Hz, $^2J_{HH}$ = 8.98 Hz, 4 H, 2,3-*c*Pr), 1.93 (dd, $^3J_{HH}$ = 5.75 Hz, $^2J_{HH}$ = 10.68 Hz, 4 H, 2,3-*c*Pr), 2.52 (m, 2 H, 1-*c*Pr) ppm.

^{1}H NMR (300 MHz, C_6D_6) δ = 1.44-1.53 (m, 4 H, 2,3-*c*Pr), 1.84-1.92 (m, 4 H, 2,3-*c*Pr), 1.93-2.05 (m, 2 H, 1-*c*Pr) ppm.

^{1}H NMR (300 MHz, CD_2Cl_2) δ = 1.66-1.75 (m, 4 H, 2,3-*c*Pr), 1.86-1.94 (m, 4 H, 2,3-*c*Pr), 2.42-2.55 (m, 2 H, 1-*c*Pr) ppm.

^{13}C{^{1}H} NMR (126 MHz, $CDCl_3$): δ = 3.54 (s, *C*H$_2$, 2,3-*c*Pr), 48.94 (br s, *C*H, 1-*c*Pr) ppm.

HRMS (LIFDI): calculated for [$BiC_6H_{10}Cl$]$^+$ (326.58 g/mol): m/z = 326.02749, found: m/z = 326.02712; calculated for [BiC_6H_{10}]$^+$: m/z = 291.0582, found: m/z = 291.05795.

IR (cm^{-1}): 3063 (m), 2989 (s), 1427 (m), 1221 (m), 1181 (s), 1092 (w), 1053 (m), 1024 (s), 854 (s), 808 (s).

1.8. Preparation of [Bi(*c*Pr)₂(SbF₆)] (5)

Bi(*c*Pr)$_2$Cl (**3-Cl**) (200 mg containing 9 eq. DCM, 0.18 mmol, 1.0 eq.) was dissolved in dichloromethane (2 mL) and added to a solution of $AgSbF_6$ (63 mg, 0.18 mmol, 1.0 eq.) in dichloromethane (3 mL) at ambient temperature. The colorless precipitate that was formed during the reaction was separated from the yellow solution by filtration and extracted with dichloromethane (2 mL). The combined phases of dichloromethane were layered with *n*-pentane (10 mL) to form crystals of [Bi(*c*Pr)$_2$(SbF_6)] in the freezer (–30 °C) after three days. The crystals were isolated by filtration and drying *in vacuo* (55 mg, 0.10 mmol, 55%).

^{1}H NMR (500 MHz, CD_2Cl_2): 2.65-2.84 (m, 4 H, 2,3-*c*Pr), 3.30-3.49 (m, 4 H, 2,3-*c*Pr), 4.89-5.09 (m, 2 H, 1-*c*Pr) ppm.

$^{13}C\{^1H\}$ NMR (126 MHz, CD_2Cl_2): δ = 4.96 (s, *C*H_2, 2,3-*c*Pr), 98.62 (br s, *C*H, 1-*c*Pr) ppm.

$^{19}F\{^1H\}$ NMR (283 MHz, CD_2Cl_2): δ = –125 (br, s, Sb*F*$_6$) ppm.

Elemental analysis: calculated for [$C_6H_{10}BiSbF_6$] (526.88 g/mol): C 13.68, H 1.91; found: C 13.33, H 2.22.

IR (cm^{-1}): 1228 (w), 1185 (m), 1030 (m), 855 (m), 659 (s), 576 (s), 469 (w).

1.9. Preparation of [Pt(PCy_3)$_2$Bi(*c*Pr_2)(SbF_6)] (8)

[Bi(*c*Pr)$_2$(SbF_6)] (**5**) (20 mg, 0.038 mmol, 1.0 eq.) was dissolved in 1,2-difluorobenzene (2 mL) and added to a solution of [Pt(PCy_3)$_2$] (29 mg, 0.038 mmol, 1.0 eq) in 1,2-difluorobenzene (4 mL) at ambient temperature. The reaction mixture turns dark red upon addition. After removing the solvent *in vacuo*, the crude product was dissolved in dichloromethane (1 mL) and then precipitated by addition of *n*-pentane (15 mL). After filtration and drying *in vacuo* [Pt(PCy_3)$_2$Bi(*c*Pr)$_2$(SbF_6)] was isolated as a red powder (30 mg, 0.023 mmol, 61%).

^1H NMR (300 MHz, CD_2Cl_2): δ = 1.26-1.36 (br, m, 18 H, axial or equatorial 6 H of 4-PCy_3 and axial or equatorial 12 H of 2,6-PCy_3), 1.41-1.55 (br, m, 4 H, Bi-*c*Pr C*H*$_2$-2/3), 1.56-1.71 (br, m, 12 H, axial or equatorial 12 H of 3,5-PCy_3), 1.73-1.85 (br, m, 10 H, axial or equatorial 6 H of 4-PCy_3, 4H of Bi-*c*Pr C*H*$_2$-2/3), 1.87-1.96 (br, m, 12 H, axial or equatorial 12 H of 2,6-PCy_3), 1.97-2.14 (br, m, 12 H, axial or equatorial 12 H of 3,5-PCy_3), 2.17-2.35 (br, m, 6 H, 1-PCy_3), 3.45-3.65 (m, 2 H, Bi-*c*Pr C*H*-1) ppm.

$^{13}C\{^1H\}$ NMR (75 MHz, CD_2Cl_2): δ = 5.34 (s, Bi-*c*Pr *C*2/3), 5.54(s, Bi-*c*Pr *C*2/3), 26.51 (s, 4-PCy_3), 27.93 (t, $^2J_{PC}$ = 5.56 Hz, 2,6-PCy_3), 31.66 (s, 3,5-PCy_3), 36.19 (t, $^1J_{PC}$ = 13.21 Hz, 1-PCy_3), 37.17 (s, Bi-*c*Pr *C*1, detected *via* ^1H-^{13}C-HSQC spectrum) ppm.

$^{19}F\{^1H\}$ NMR (283 MHz, CD_2Cl_2): δ = –106 to –143 (br, m, Sb*F*$_6$) ppm.

$^{31}P\{^1H\}$ NMR (122 MHz, CD_2Cl_2) δ = 52.8 (s, $^1J_{PtP}$ = 2949 Hz) ppm.

HRMS (LIFDI): calculated for [$C_{42}H_{76}BiP_2Pt$]$^+$: m/z = 1046.4872, found: m/z = 1046.4864; [$C_{39}H_{71}P_2Pt$]$^+$: m/z = 796.4677, found: m/z = 796.4673.

HRMS (ESI): calculated for [$C_{42}H_{76}BiP_2Pt$]$^+$: m/z = 1046.4872, found: m/z = 1046.4864; [$C_{39}H_{71}P_2Pt$]$^+$: m/z = 796.4677, found: m/z = 796.4664.

Elemental analysis: calculated for [$C_{42}H_{76}BiF_6P_2PtSb$] (1282.83 g/mol): C 39.32, H 5.97; found: C 39.04, H 6.08.

IR (cm^{-1}): 2922 (m), 2849 (m), 1446 (m), 1175 (w), 1003 (w), 849 (m), 738 (w), 652 (s), 513 (m).

1.10. Preparation of [Bi(*i*Pr)$_2$(SbF$_6$)] (6)

[Bi(*i*Pr)$_2$Cl] (100 mg, 0.30 mmol, 1.0 eq.) was dissolved in dichloromethane (5 mL) and added to a solution of AgSbF$_6$ (104 mg, 0.30 mmol, 1.0 eq.) in dichloromethane (5 mL) at ambient temperature. Upon addition the solution turned bright orange and a colorless precipitate was formed. After filtration, the precipitate was extracted with dichloromethane (2 x 4 mL). All dichloromethane phases were combined and all volatiles removed under reduced pressure (10^{-3} mbar) leading to the isolation of [Bi(*i*Pr)$_2$(SbF$_6$)] (95 mg, 0.18 mmol 60%)

^{1}H NMR (300 MHz, CD_2Cl_2): δ = 3.72 (sept, 2 H, $^3J_{HH}$ = 7.37 Hz, C*H*(CH$_3$)$_2$), 4.01 (d, 12 H, $^3J_{HH}$ = 7.37 Hz, CH(C*H*$_3$)$_2$) ppm.

^{13}C{^{1}H} NMR (75 MHz, CD_2Cl_2): δ = 19.48 (s, CH(*C*H$_3$)$_2$), 119.48 (s, *C*H(CH$_3$)$_2$) ppm.

^{19}F{^{1}H} NMR (283 MHz, CD_2Cl_2): δ = –124 (br, s, Sb*F*$_6$) ppm.

Elemental analysis: calculated for [$C_6H_{14}BiSbF_6$] (530.91 g/mol): C 13.57, H 2.66; found: C 13.84, H 2.77.

IR (cm^{-1}): 2864 (m), 1459 (m), 1371 (w), 1181 (m), 1138 (s), 844 (w), 658 (s), 633 (s), 569 (s).

1.11. Preparation of [Pt(PCy$_3$)$_2$Bi(*i*Pr$_2$)(SbF$_6$)] (9)

[Bi(*i*Pr)$_2$(SbF$_6$)] (**6**) (120 mg, 22.6 mmol, 1.0 eq.) was dissolved in 1,2-difluorobenzene (4 mL) and added to a solution of [Pt(PCy$_3$)$_2$] (171 mg, 22.6 mmol, 1.0 eq.) in 1,2-difluorobenzene (6 mL) at ambient temperature. The reaction mixture turns dark green and was stirred for 1 h at room temperature. All volatiles were removed *in vacuo* and the crude product was dissolved in dichloromethane (2 mL) and layered with *n*-pentane (6 mL). At –30 °C dark green crystals formed after three days, that were isolated by filtration and drying *in vacuo* (271 mg, 21.1 mmol, 93%)

^{1}H NMR (500 MHz, CD_2Cl_2, 300 K): δ = 1.29-1.36 (br, m, 18 H, axial or equatorial 6 H of 4-PCy$_3$ and axial or equatorial 12 H of 2,6-PCy$_3$), 1.55-1.68 (br, m, 12 H, axial or equatorial 12 H of 3,5-PCy$_3$), 1.80-1.83 (br, m, 6 H, axial or equatorial 6 H of 4-PCy$_3$), 1.90-1.96 (br, m, 12 H, axial or equatorial 12 H of 2,6-PCy$_3$), 1.98-2.08 (br, m, 12 H, axial or equatorial 12 H of 3,5-PCy$_3$), 2.20-2.35 (br, m, 6 H, 1-PCy$_3$), 2.51 (br, s, 3 H, Bi–*i*Pr C*H*$_3$), 3.39 (br, s, 3 H, Bi–*i*Pr C*H*$_3$), 4.00 (sept, $^3J_{HH}$ = 7.42 Hz, 1 H, Bi–*i*Pr C*H*) ppm.

^{1}H NMR (500 MHz, CD_2Cl_2, 273 K): δ = 1.26-1.32 (br, m, 18 H, axial or equatorial 6 H of 4-PCy$_3$ and axial or equatorial 12 H of 2,6-PCy$_3$), 1.53-1.64 (br, m, 12 H, axial or equatorial 12 H of 3,5-PCy$_3$), 1.78-1.81 (br, m, 6 H, axial or equatorial 6 H of 4-PCy$_3$), 1.88-1.93 (br, m, 12 H, axial or equatorial 12 H of 2,6-PCy$_3$), 1.95-2.02 (br, m, 12 H, axial or equatorial 12 H of 3,5-PCy$_3$), 2.18-2.31 (br, m, 6 H, 1-PCy$_3$), 2.44 (d, $^3J_{HH}$ = 7.11 Hz, 3 H, Bi–*i*Pr C*H*$_3$), 3.38 (d, $^3J_{HH}$ = 6.94 Hz, 3 H, Bi–*i*Pr C*H*$_3$), 3.98 (sept, $^3J_{HH}$ = 7.47 Hz, 1 H, Bi–*i*Pr C*H*) ppm.

$^{13}C\{^1H\}$ NMR (126 MHz, CD_2Cl_2, 300 K): δ = 20.33 (br, s, Bi–*i*Pr CH_3), 24.30 (br, s, Bi–*i*Pr CH_3), 26.46 (s, 4-PCy_3), 27.94 (t, $^2J_{PC}$ = 5.48 Hz, 2,6-PCy_3), 31.50 (s, 3,5-PCy_3), 36.14 (t, $^1J_{PC}$ = 13.08 Hz, 1-PCy_3), 65.07 (s, Bi–*i*Pr *C*H) ppm.

$^{19}F\{^1H\}$ NMR (283 MHz, CD_2Cl_2): δ = –105 to –140 (br, m, SbF_6) ppm.

$^{31}P\{^1H\}$ NMR (122 MHz, CD_2Cl_2) δ = 58.9 (s, $^1J_{PtP}$ = 2950 Hz) ppm.

HRMS (ESI): calculated for $[C_{42}H_{80}BiP_2Pt]^+$: m/z = 1050.5185, found: m/z = 1050.5171.

Elemental analysis: calculated for $[C_{42}H_{80}BiF_6P_2PtSb]$ (1286.99 g/mol): C 39.20, H 6.27; found: C 38.83, H 6.25.

IR (cm^{-1}): 2922 (m), 2849 (m), 1445 (m), 1174 (w), 1127 (w), 1003 (w), 887 (w), 849 (w), 737 (w), 653 (s), 511 (m).

2. NMR Spectra

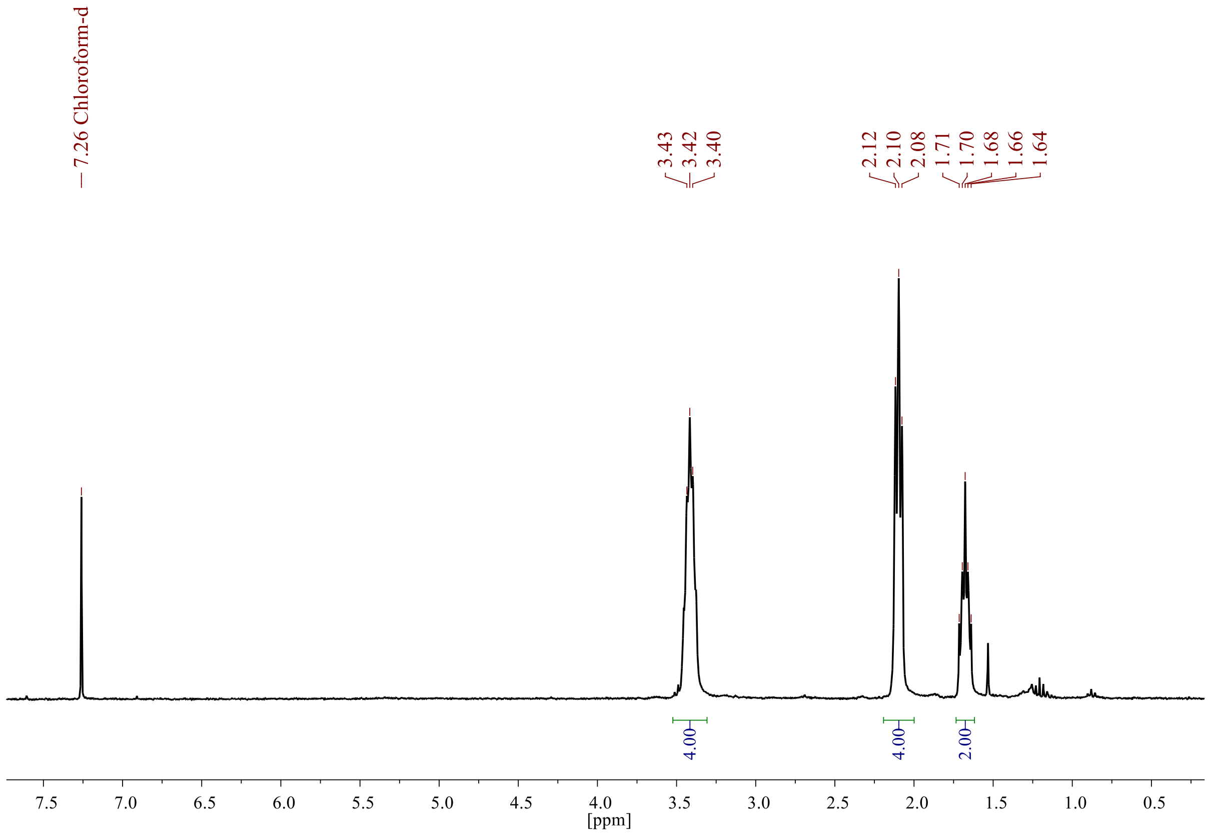

Figure S1: ^{1}H NMR spectrum of $[Bi(CH_2)_5Br]$ (**1**) in $CDCl_3$.

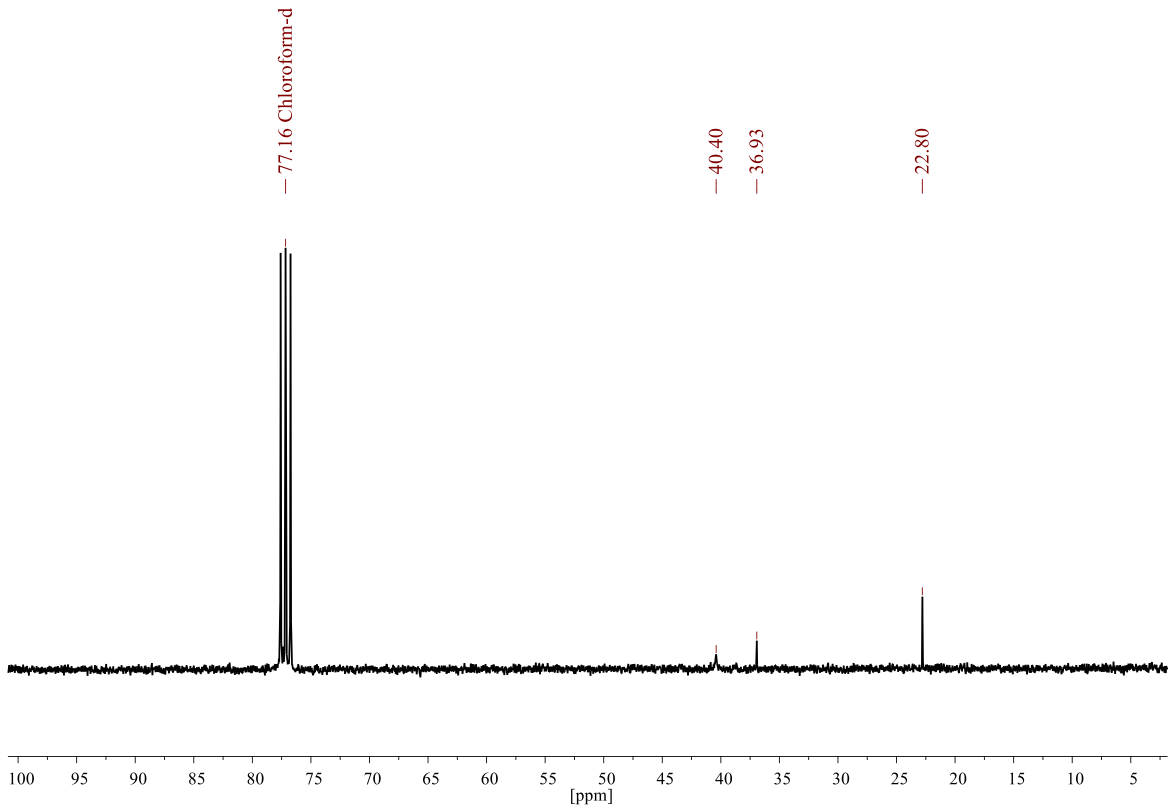

Figure S2: ^{13}C NMR spectrum of $[Bi(CH_2)_5Br]$ (**1**) in $CDCl_3$.

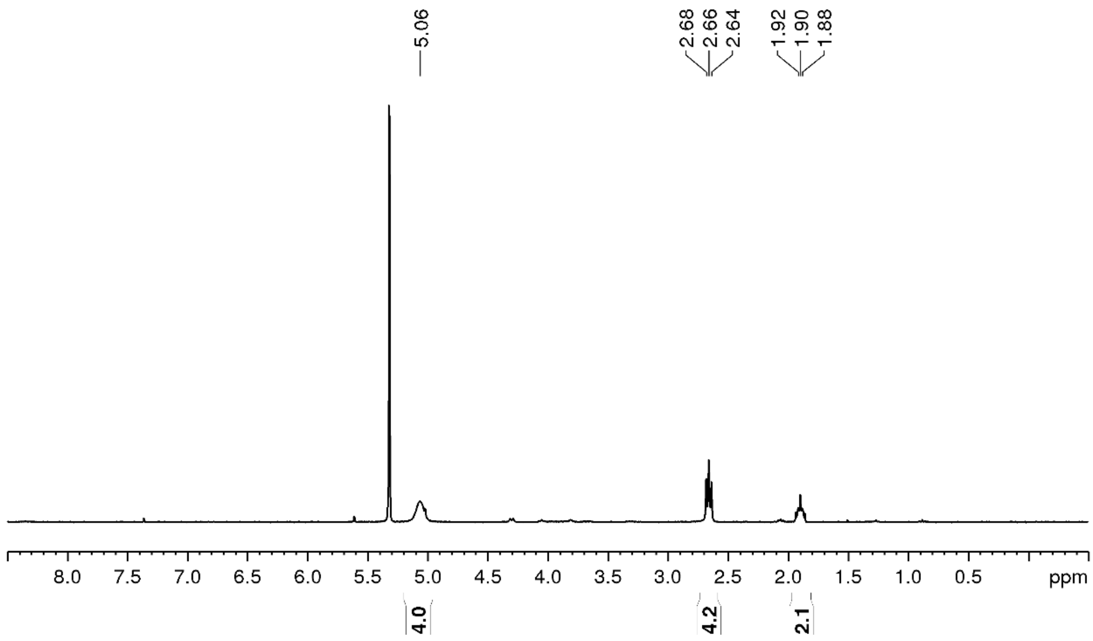

Figure S3: ^{1}H NMR spectrum of $[Bi(CH_2)_5(SbF_6)]$ (**4**) in CD_2Cl_2.

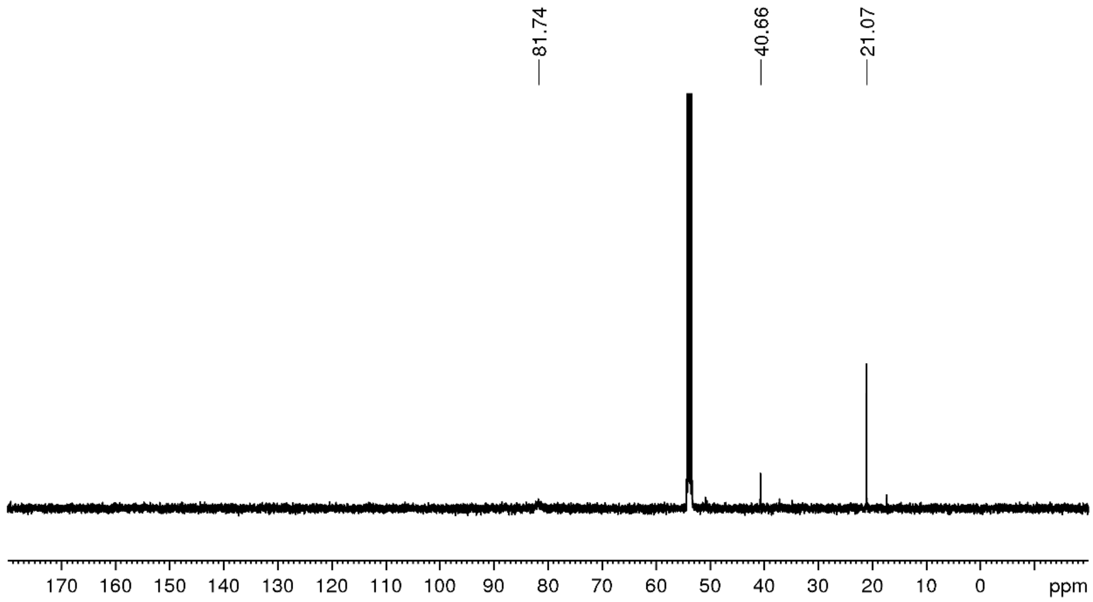

Figure S4: ^{13}C NMR spectrum of $[Bi(CH_2)_5(SbF_6)]$ (**4**) in CD_2Cl_2.

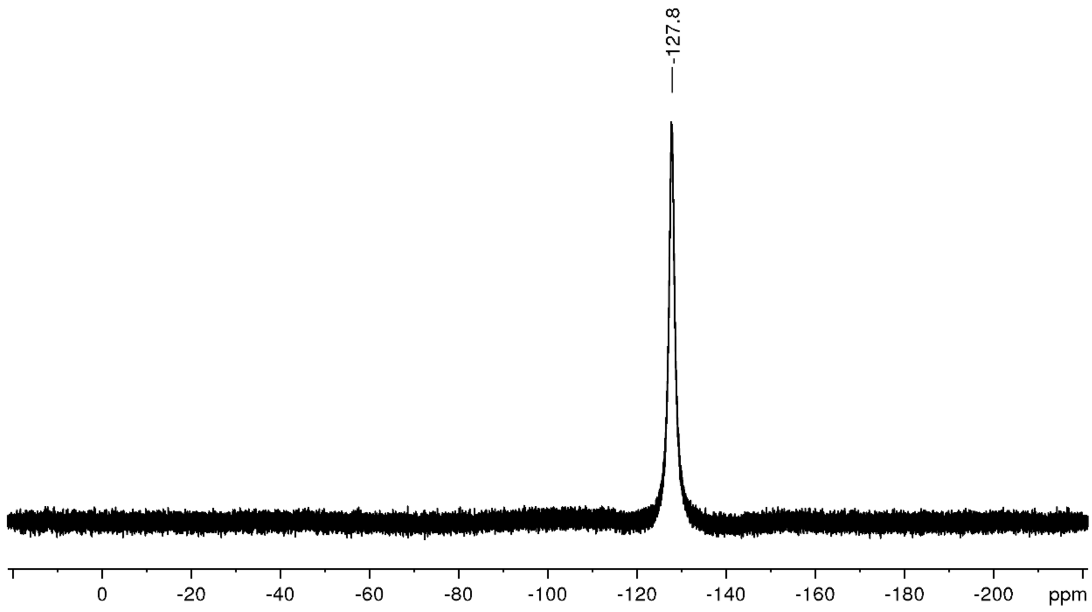

Figure S5: $^{19}F\{^1H\}$ NMR spectrum of $[Bi(CH_2)_5(SbF_6)]$ (**4**) in CD_2Cl_2.

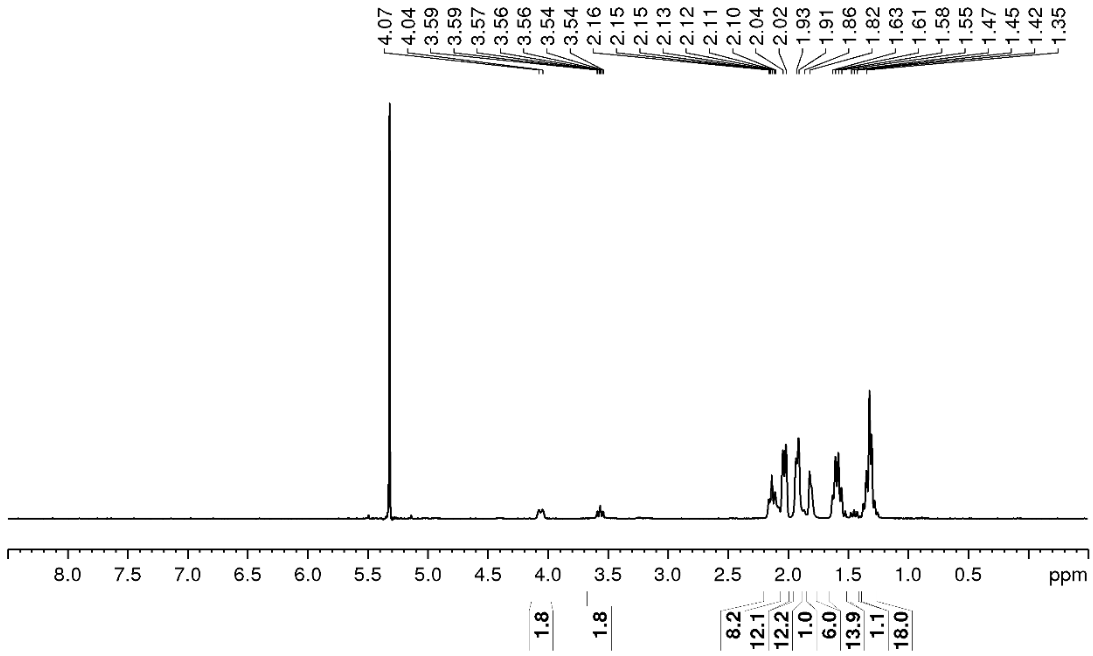

Figure S6: ^1H NMR spectrum of $[Pt(PCy_3)_2(Bi(CH_2)_5)(SbF_6)]$ (**7**) in CD_2Cl_2.

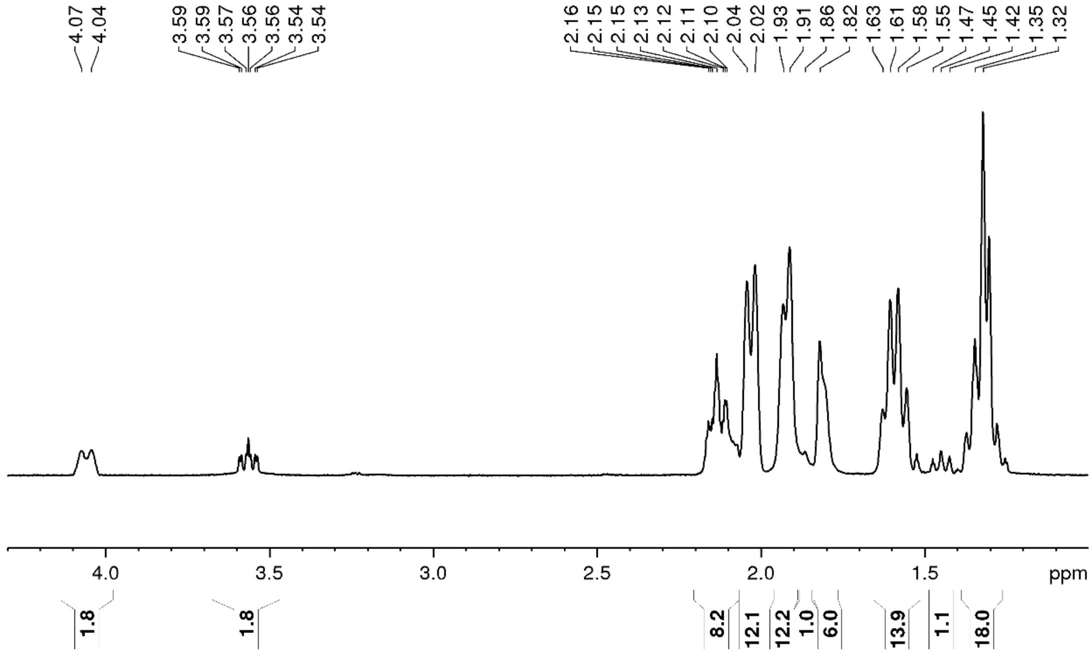

Figure S7: Enlargement of the ^{1}H NMR spectrum of $[Pt(PCy_3)_2(Bi(CH_2)_5)(SbF_6)]$ (**7**) in CD_2Cl_2 between 1.2 and 4.2 ppm.

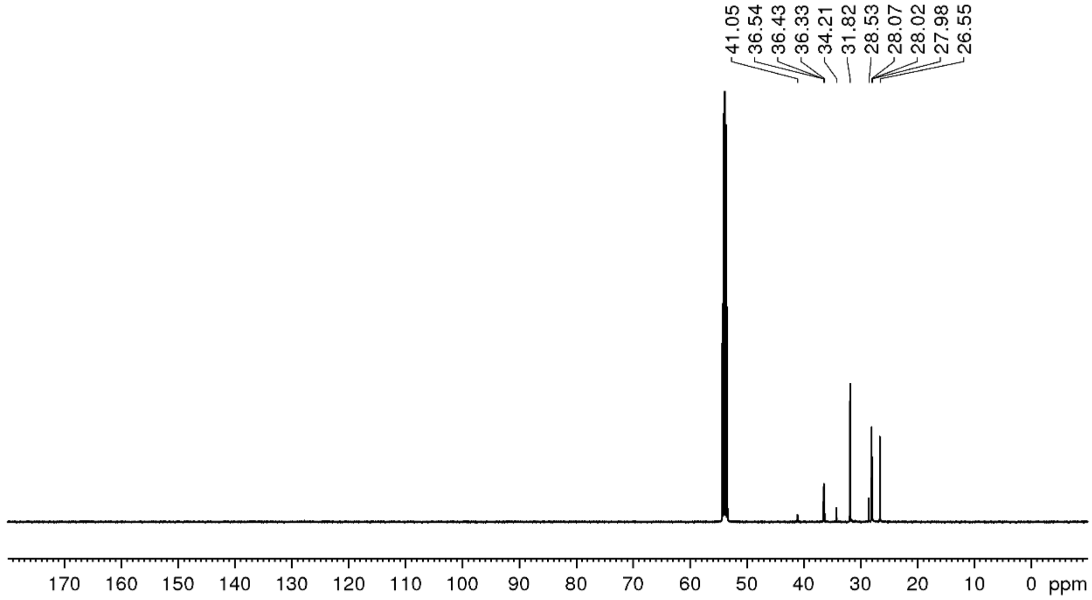

Figure S8: ^{13}C NMR spectrum of $[Pt(PCy_3)_2(Bi(CH_2)_5)(SbF_6)]$ (**7**) in CD_2Cl_2.

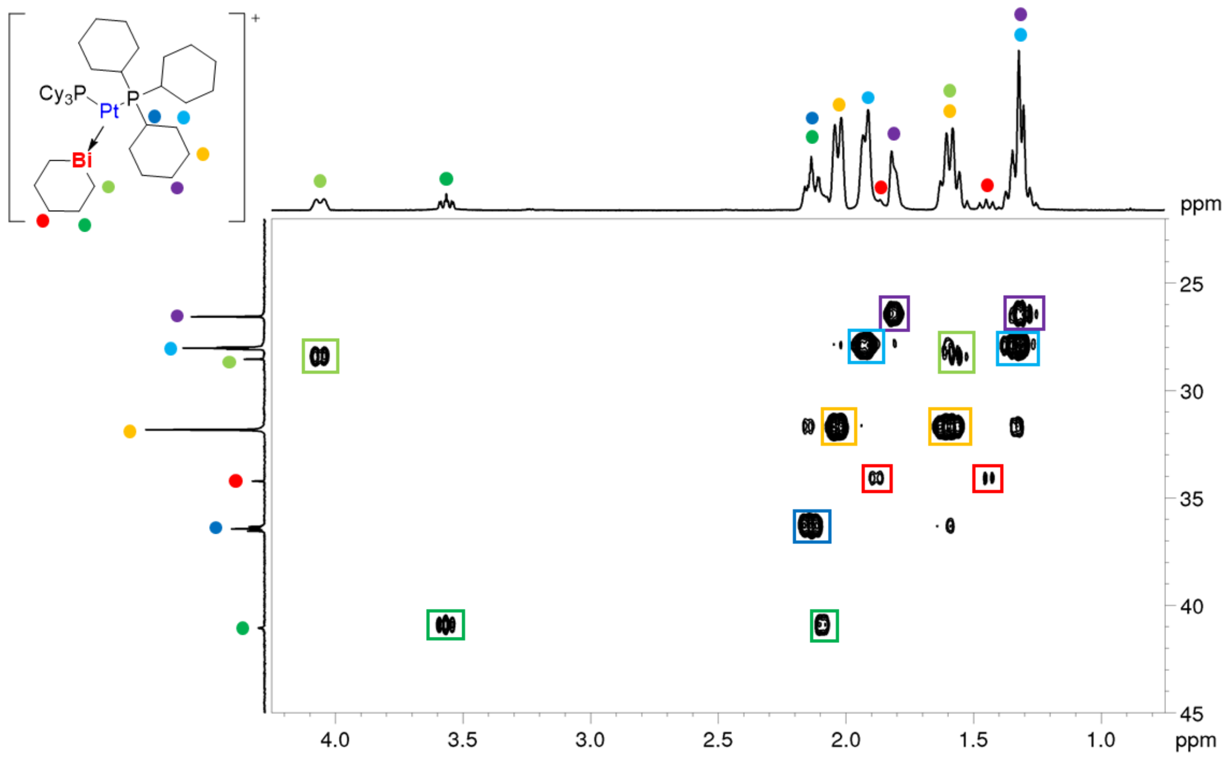

Figure S9: ^{1}H-^{13}C HSQC spectrum of [Pt(PCy$_3$)$_2$Bi(CH$_2$)$_5$(SbF$_6$)] (**7**) in CD$_2$Cl$_2$. All resonances are assigned to one signal in the ^{13}C NMR spectrum and one or two signals in the ^{1}H NMR spectrum due to the splitting of axial and equatorial protons.

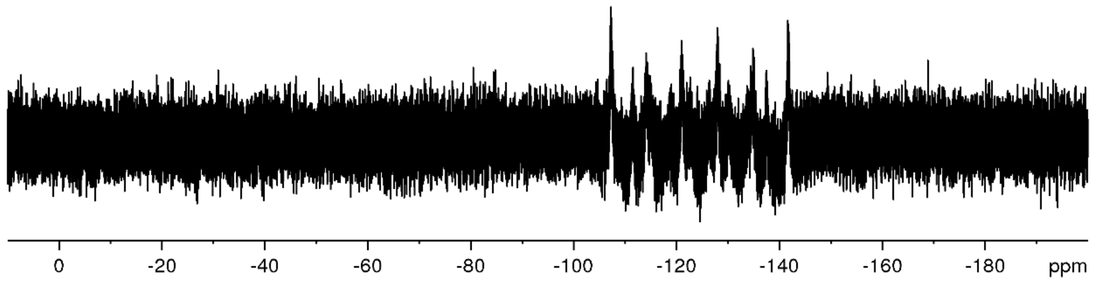

Figure S10: ^{19}F{^{1}H} NMR spectrum of [Pt(PCy$_3$)$_2$Bi(CH$_2$)$_5$(SbF$_6$)] (**7**) in CD$_2$Cl$_2$.

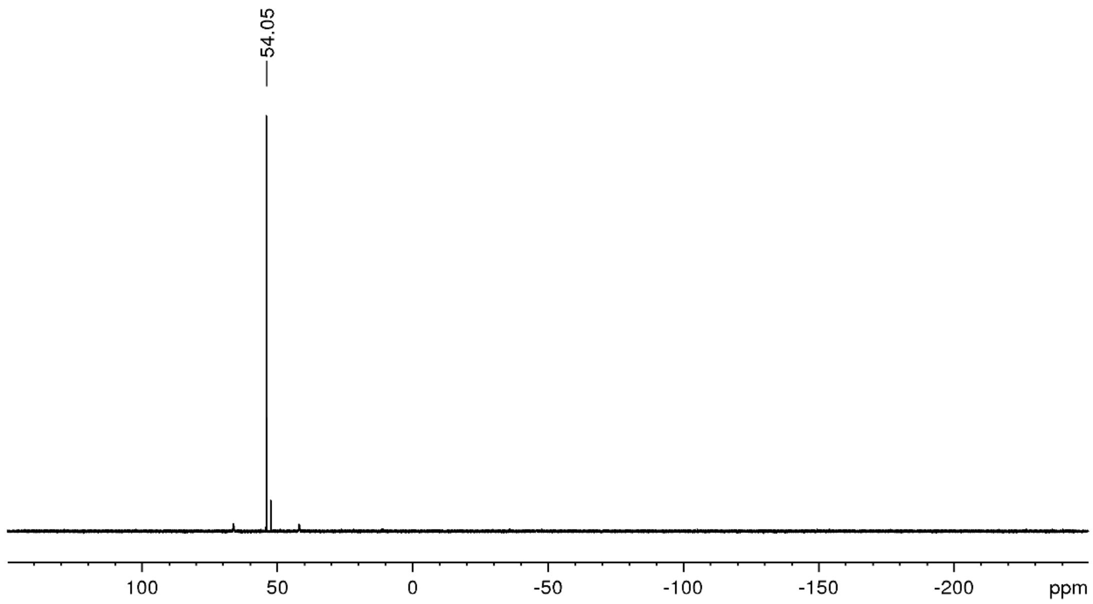

Figure S11: $^{31}P\{^1H\}$ NMR spectrum of $[Pt(PCy_3)_2Bi(CH_2)_5(SbF_6)]$ (**7**) in CD_2Cl_2.

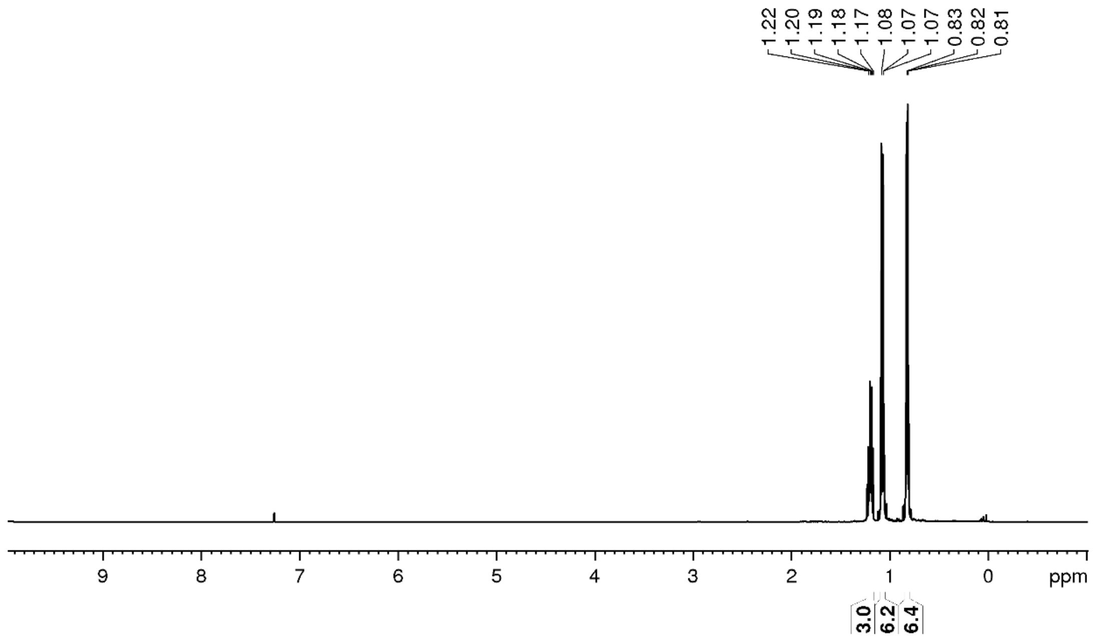

Figure S12: ^1H NMR spectrum of $[Bi(c\text{Pr})_3]$ (**2**) in $CDCl_3$.

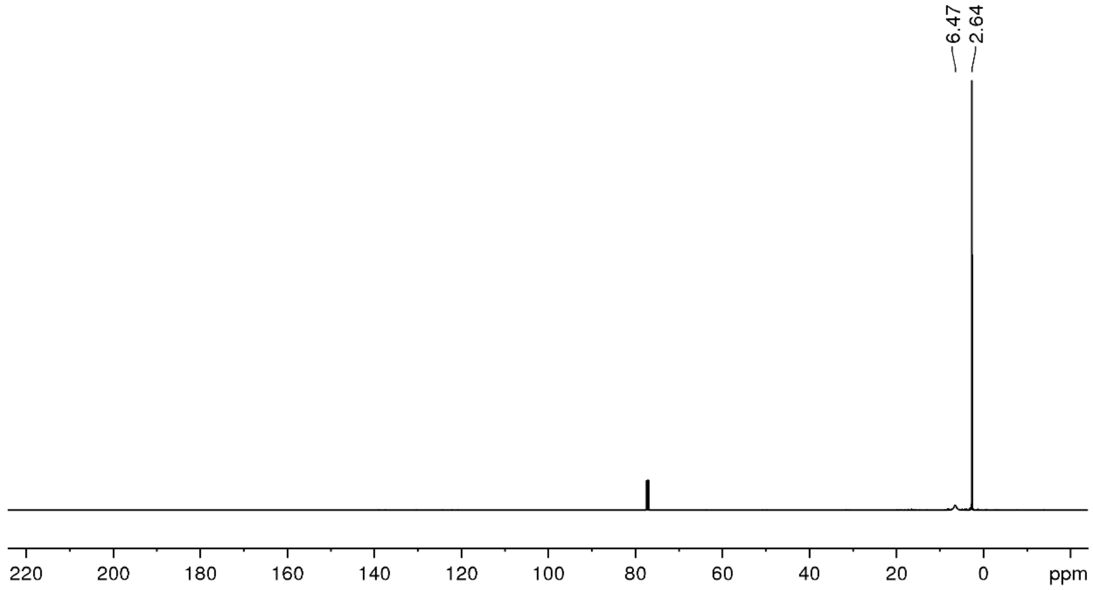

Figure S13: ^{13}C NMR spectrum of [Bi(cPr)$_3$] (**2**) in $CDCl_3$.

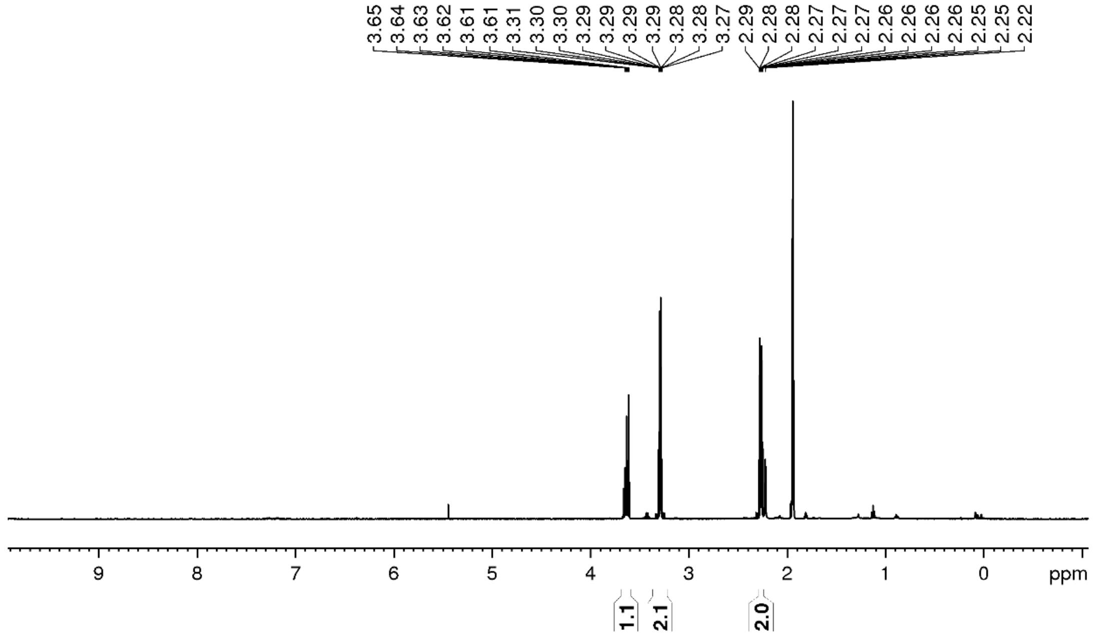

Figure S14: ^{1}H NMR spectrum of [Bi(cPr)Cl_2] (**3-Cl$_2$**) in CD_3CN.

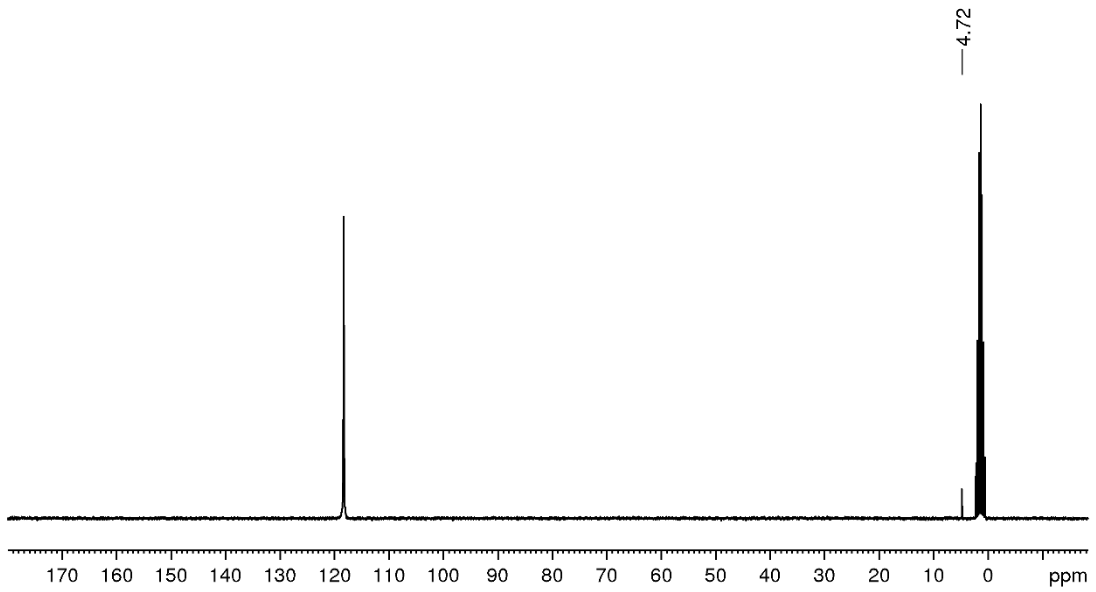

Figure S15: ^{13}C NMR spectrum of [Bi(*c*Pr)Cl$_2$] (**3-Cl$_2$**) in CD_3CN.

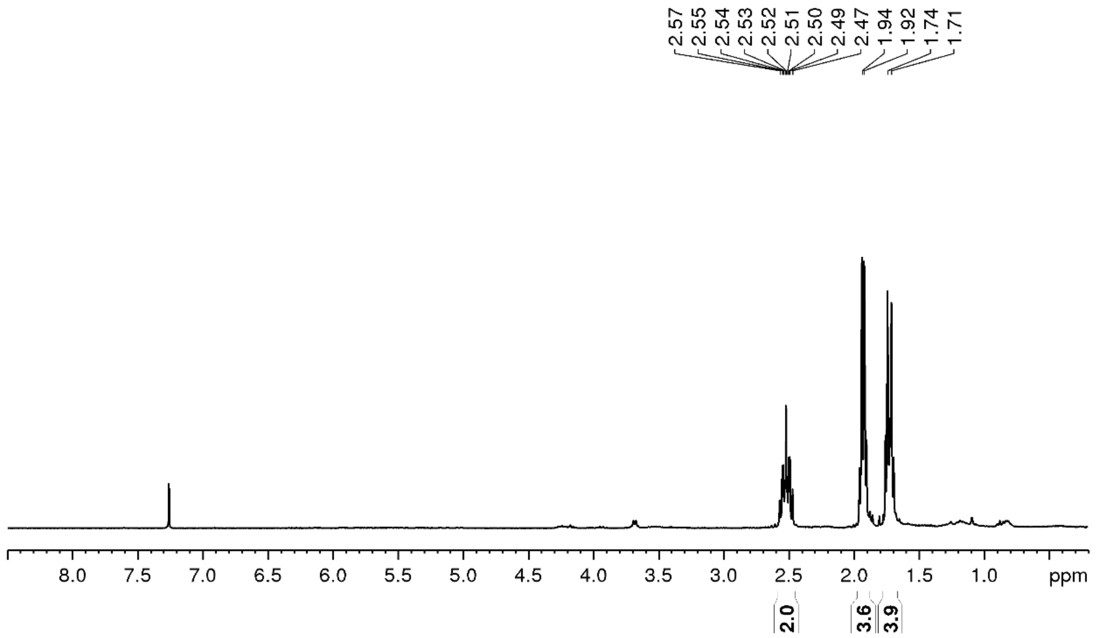

Figure S16: ^{1}H NMR spectrum of [Bi(*c*Pr)$_2$Cl] (**3-Cl**) in $CDCl_3$.

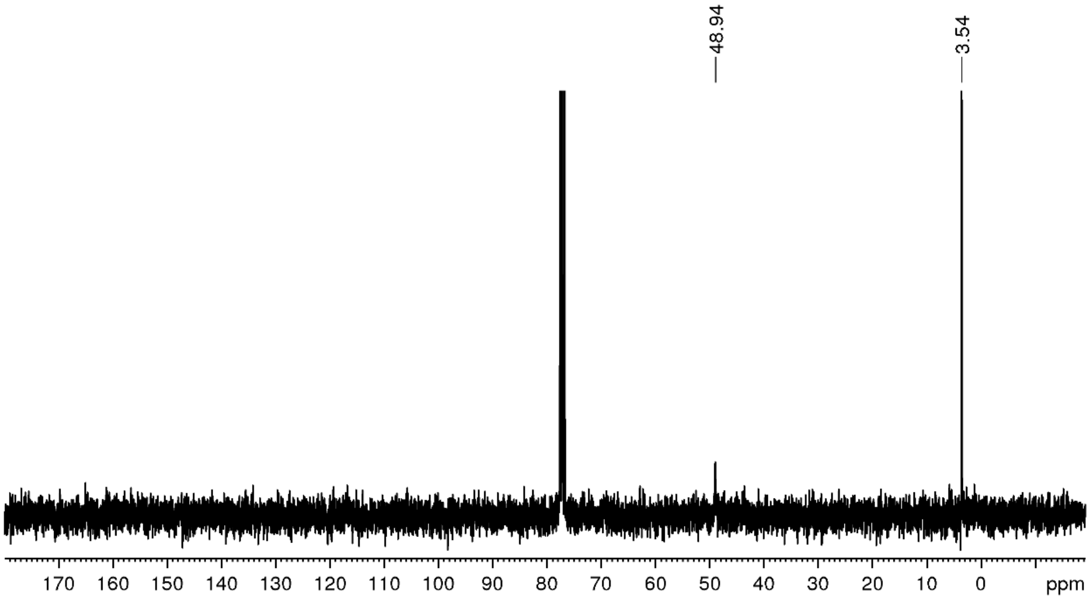

Figure S17: ^{13}C NMR spectrum of [Bi(*c*Pr)$_2$Cl] (**3-Cl**) in $CDCl_3$.

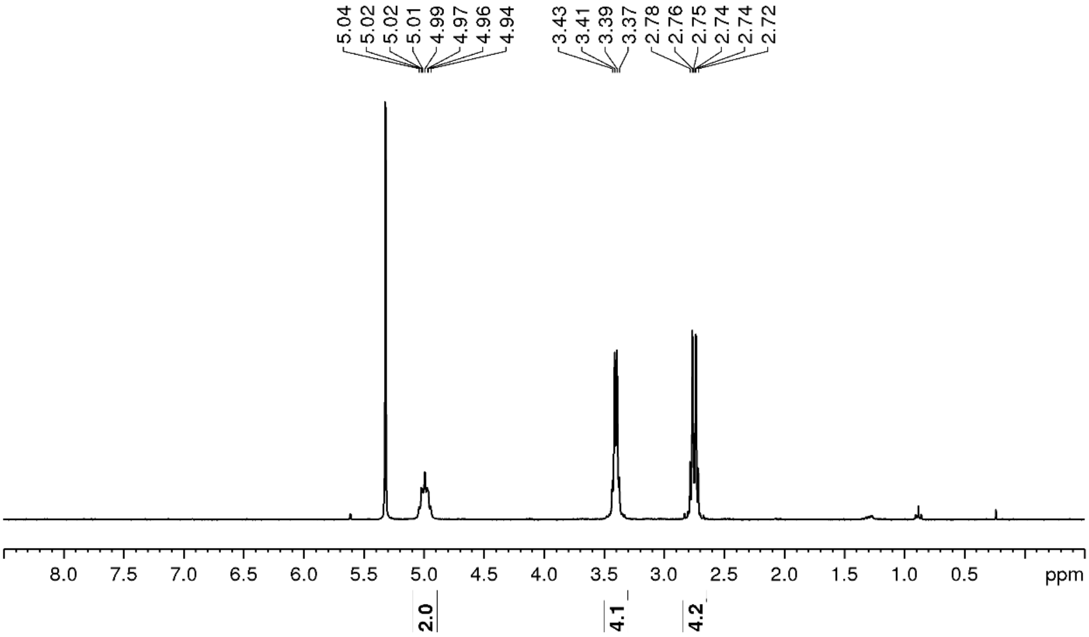

Figure S18: ^{1}H NMR spectrum of [Bi(*c*Pr)$_2$(SbF_6)] (**5**) in CD_2Cl_2.

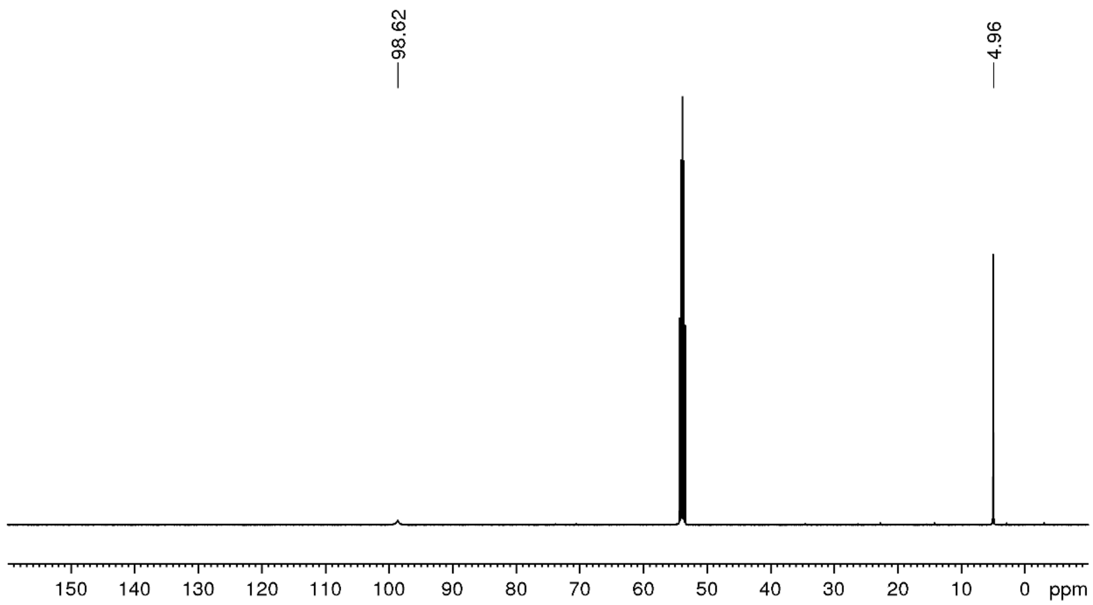

Figure S19: ^{13}C NMR spectrum of $[Bi(\textit{c}Pr)_2(SbF_6)]$ (**5**) in CD_2Cl_2.

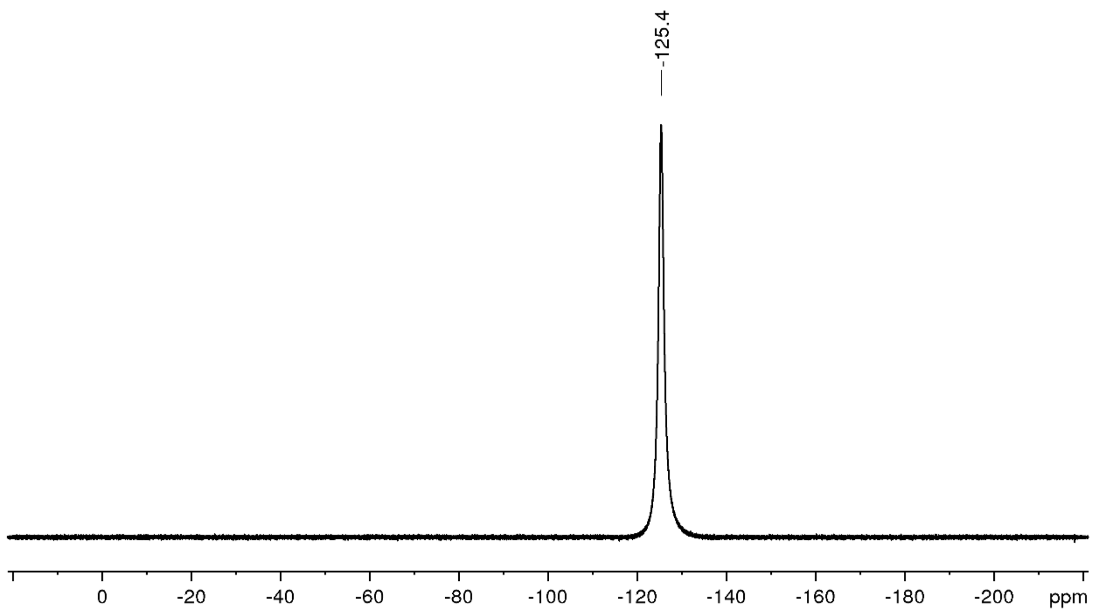

Figure S20: $^{19}F\{^1H\}$ NMR spectrum of $[Bi(\textit{c}Pr)_2(SbF_6)]$ (**5**) in CD_2Cl_2.

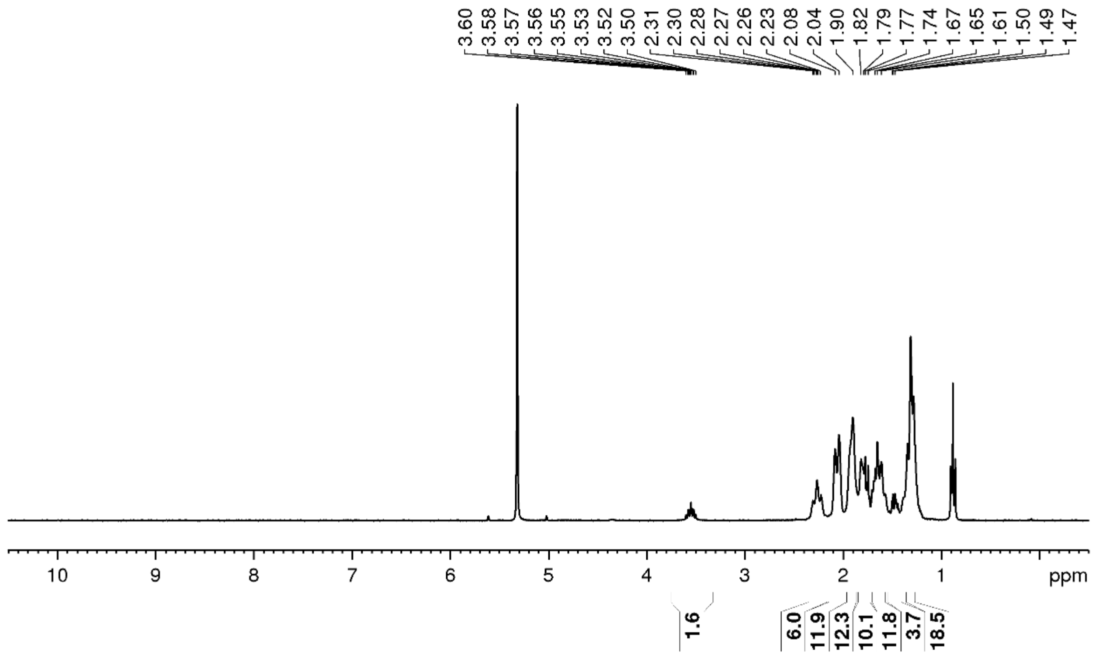

Figure S21: ^{1}H NMR spectrum of [Pt(PCy_3)$_2$Bi(*c*Pr)$_2$(SbF_6)] (**8**) in CD_2Cl_2.

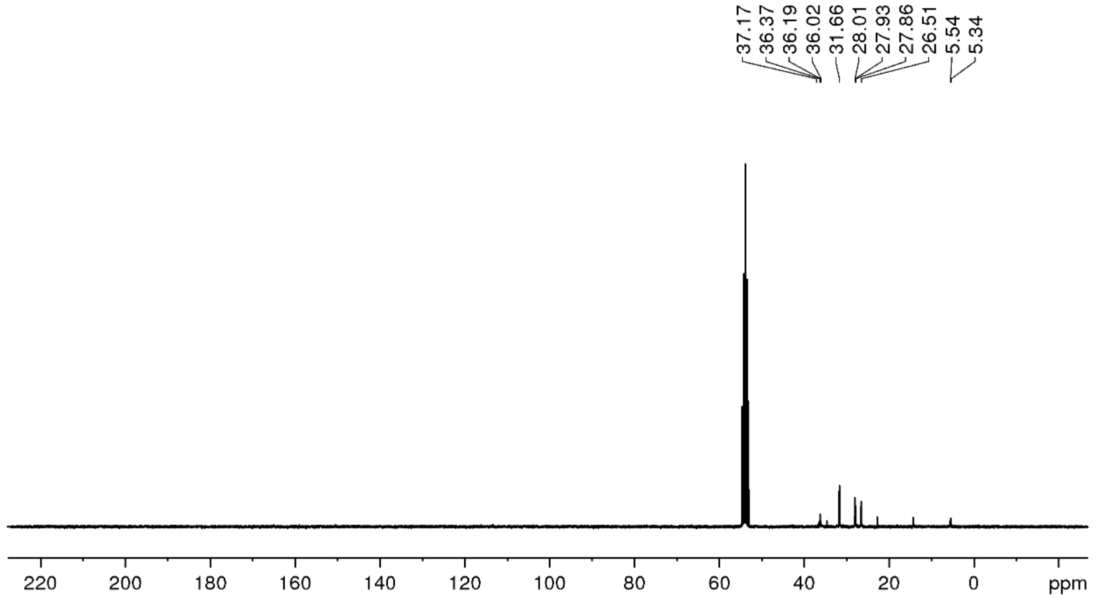

Figure S22: ^{13}C NMR spectrum of [Pt(PCy_3)$_2$Bi(*c*Pr)$_2$(SbF_6)] (**8**) in CD_2Cl_2.

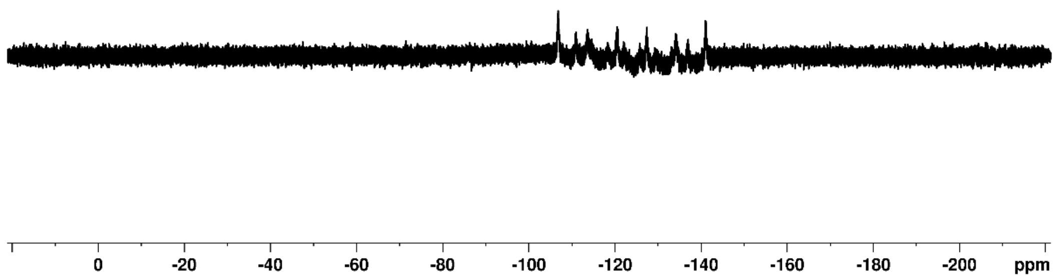

Figure S23: $^{19}F\{^{1}H\}$ NMR spectrum of $[Pt(PCy_3)_2Bi(c\text{Pr})_2(SbF_6)]$ (**8**) in CD_2Cl_2.

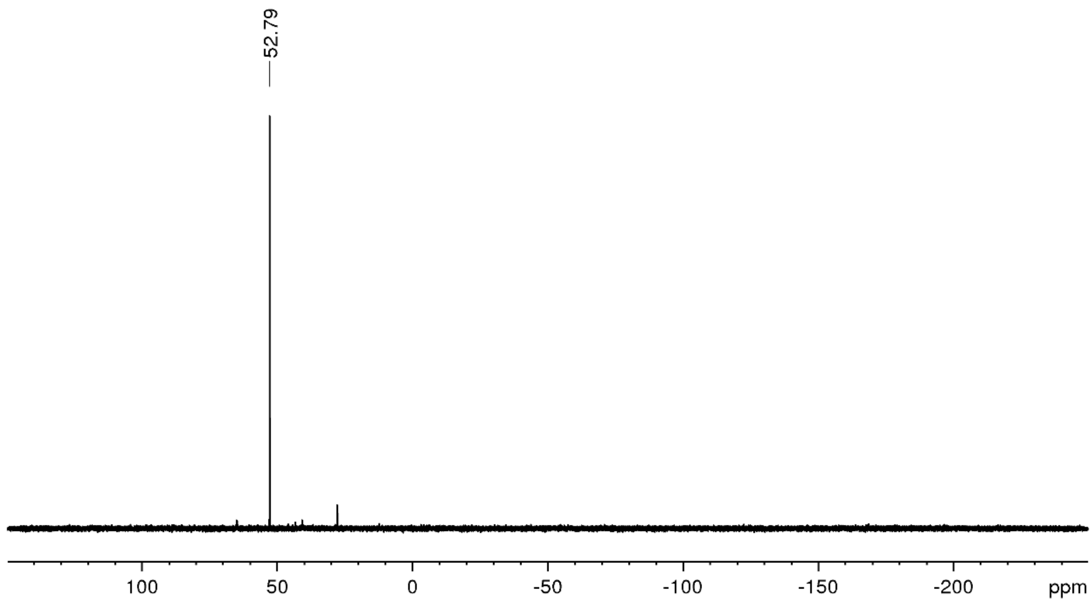

Figure S24: $^{31}P\{^{1}H\}$ NMR spectrum of $[Pt(PCy_3)_2Bi(c\text{Pr})_2(SbF_6)]$ (**8**) in CD_2Cl_2.

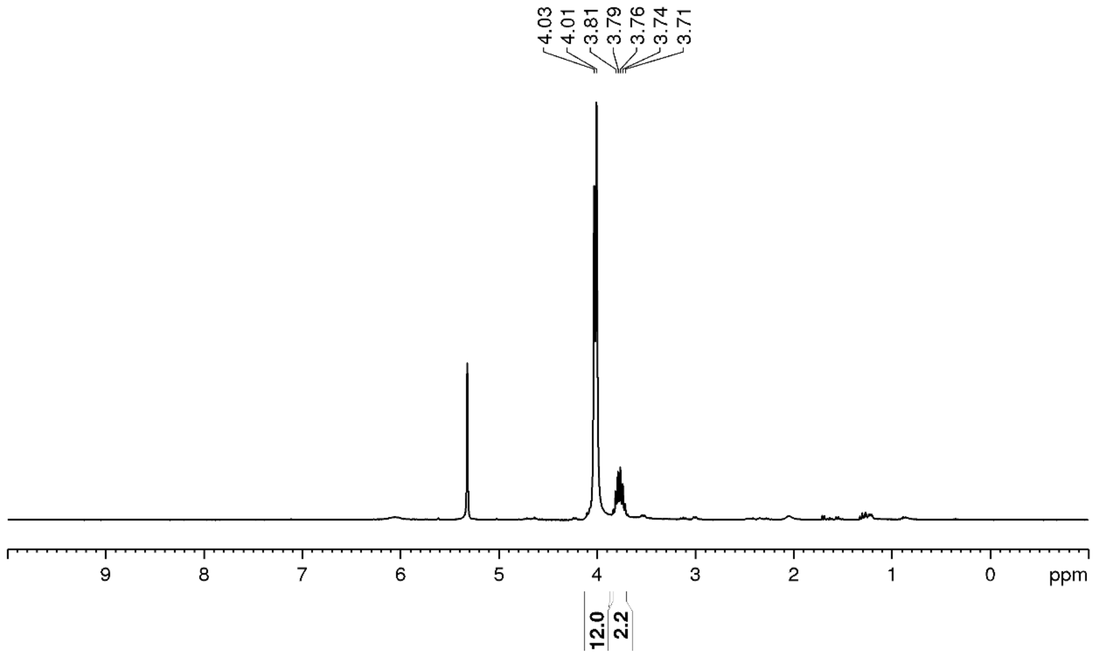

Figure S25: ^{1}H NMR spectrum of $[Bi(\textit{i}Pr)_2(SbF_6)]$ (**6**) in CD_2Cl_2.

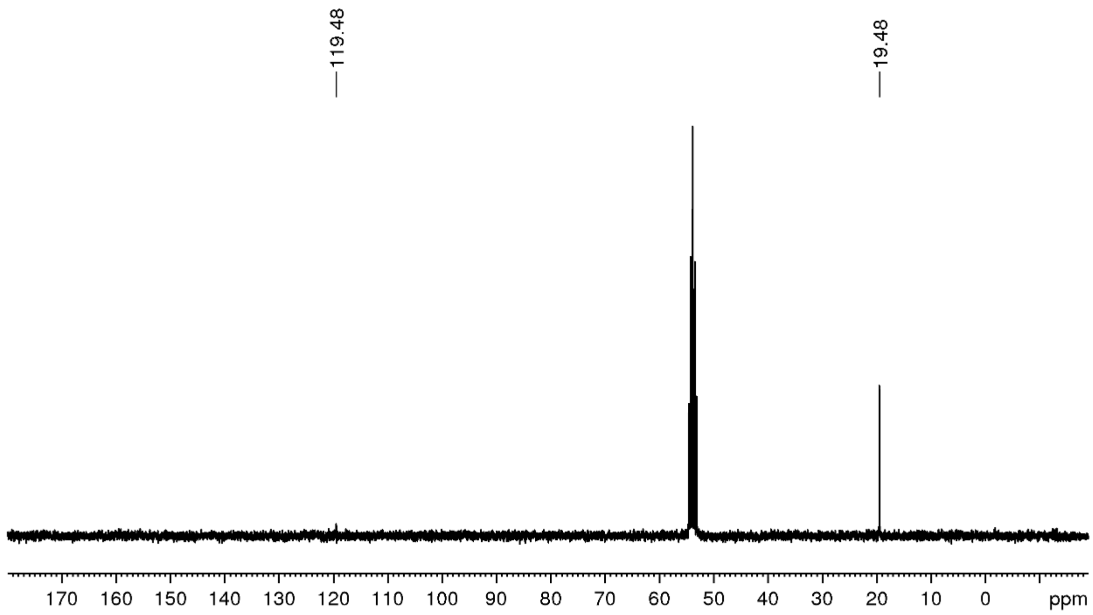

Figure S26: ^{13}C NMR spectrum of $[Bi(\textit{i}Pr)_2(SbF_6)]$ (**6**) in CD_2Cl_2.

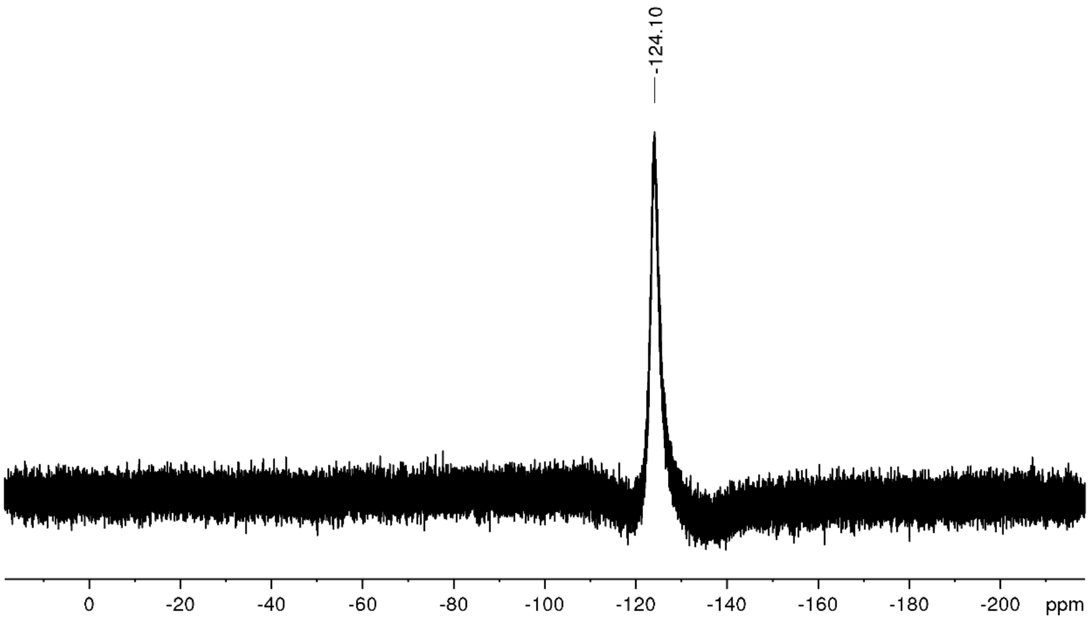

Figure S27: $^{19}F\{^1H\}$ NMR spectrum of [Bi(*i*Pr)$_2$(SbF$_6$)] (**6**) in CD_2Cl_2.

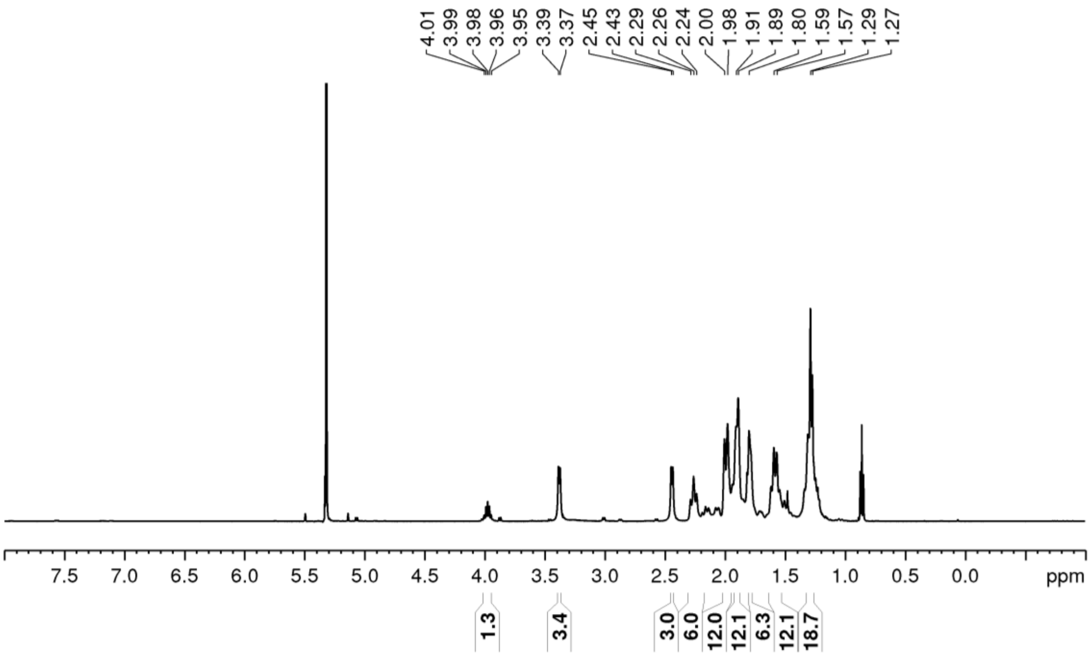

Figure S28: ^1H NMR spectrum of [Pt(PCy$_3$)$_2$Bi(*i*Pr)$_2$(SbF$_6$)] (**9**) in CD_2Cl_2 at 273 K.

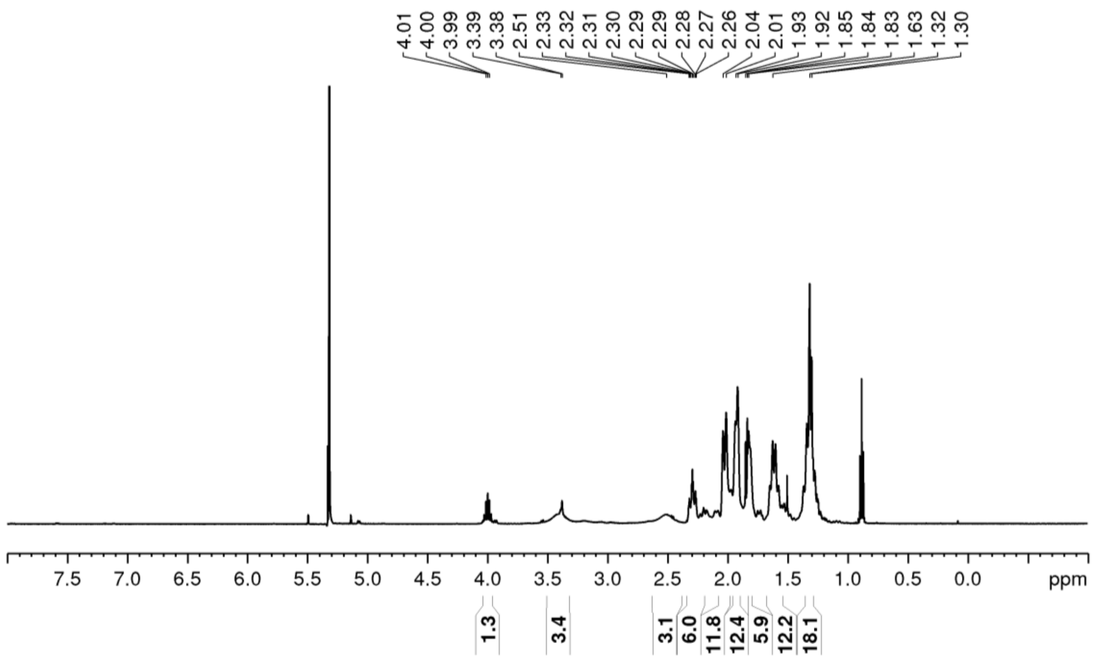

Figure S29: ^{1}H NMR spectrum of [Pt(PCy$_3$)$_2$Bi(*i*Pr)$_2$(SbF$_6$)] (**9**) in CD_2Cl_2 at 300 K.

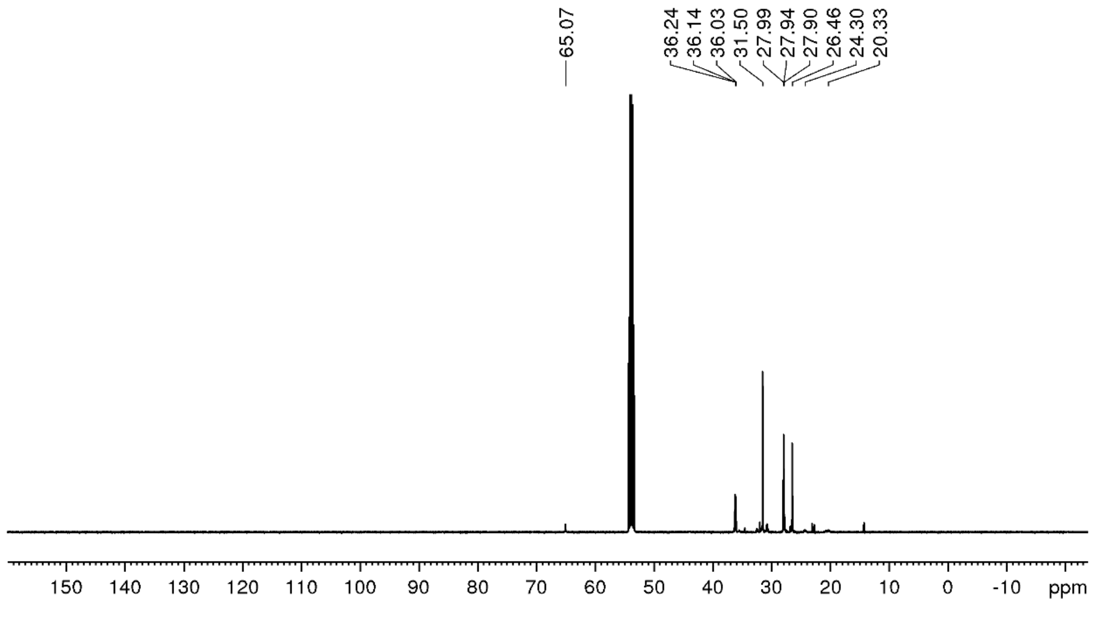

Figure S30: ^{13}C NMR spectrum of [Pt(PCy$_3$)$_2$Bi(*i*Pr)$_2$(SbF$_6$)] (**9**) in CD_2Cl_2.

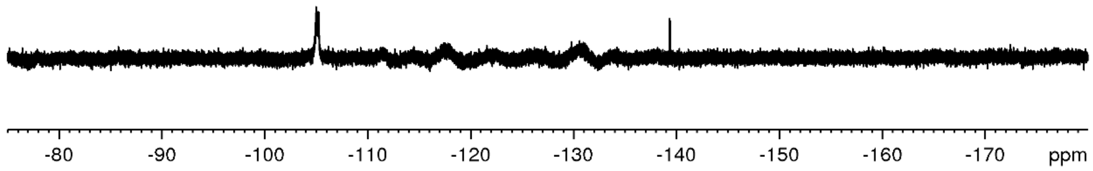

Figure S31: $^{19}F\{^{1}H\}$ NMR spectrum of $[Pt(PCy_3)_2Bi(i\text{Pr})_2(SbF_6)]$ (**9**) in CD_2Cl_2.

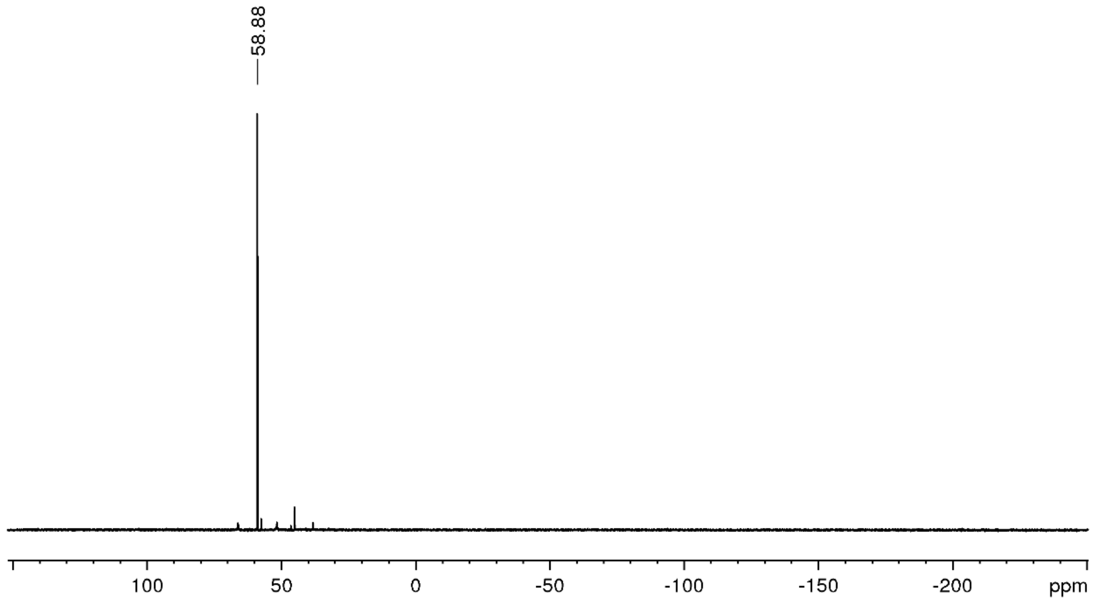

Figure S32: $^{31}P\{^{1}H\}$ NMR spectrum of $[Pt(PCy_3)_2Bi(i\text{Pr})_2(SbF_6)]$ (**9**) in CD_2Cl_2.

3. UV-Vis spectra

The intensely colored compounds **7-9** were further investigated by UV-vis spectroscopy (see main part and figures below). The spectroscopic data of the parent compound **I** (λ_{max} = 437 nm, 527 nm)[41] has been interpreted aided by theoretical investigations of the absorption processes in a two-component relativistic approach. The two main absorption bands were associated with the depletion of electron density in the Bi–C σ-bonds in the electron density difference plots.[41] This is in agreement with the trend that is observed in this work. Compounds **7** and **9** bear Bi–C bonds with a secondary and a tertiary carbon atom, respectively. Thus, inductive effects can be expected to lead to σ(Bi–C) bonding orbitals that are higher in energy and thus lead to a red-shift of the absorption bands of these compounds, which his observed experimentally. The *cyclo*propyl substituents in **8** represent a special case, since the constrained ring-structure has been argued to lend sp^2-like hybridization to the orbital that is responsible for exocyclic bond formation.[67] Thus, a higher degree of substitution, but also a higher s-character are apparent as two opposing trends when comparing the influence of the *cyclo*propyl vs. methyl group on the UV/vis spectra of **8** and **I**. As a result, the maximal of their main absorption bands are similar to each other.

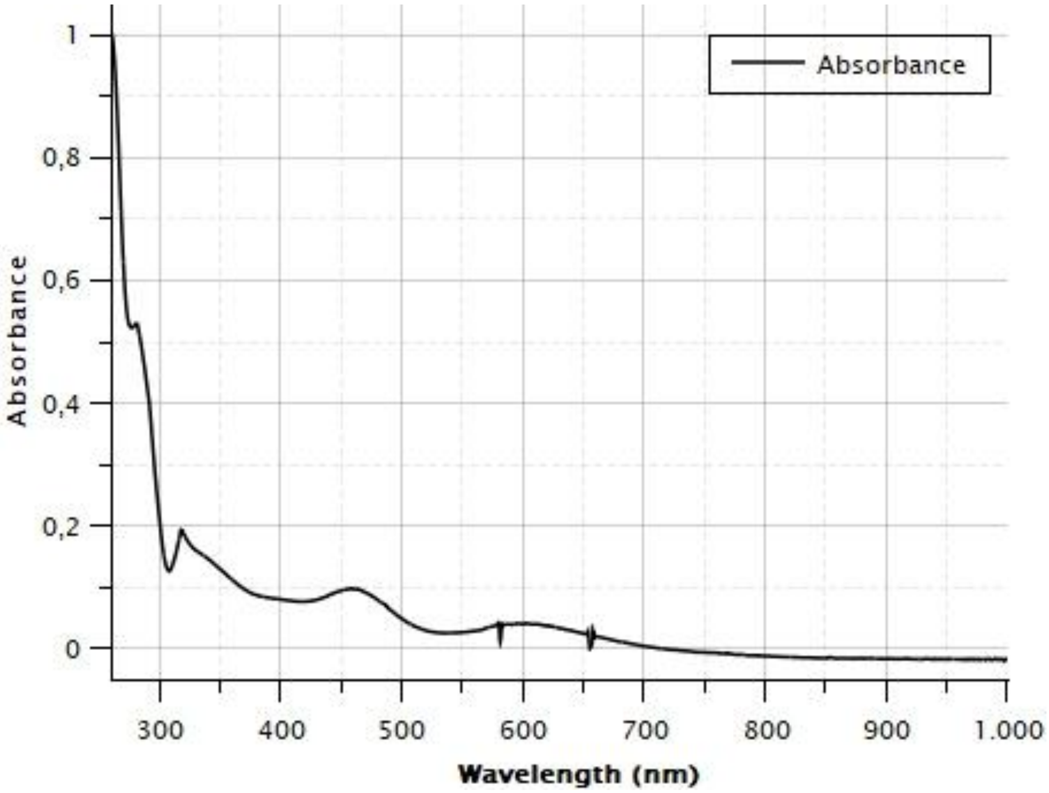

Figure S33: UV-Vis spectrum of [Pt(PCy_3)$_2$(Bi*i*Pr_2)(SbF_6)] in dichloromethane (c = 8.27·10^{-5} mol/L), λ_{max} = 318.5, 460.0, 605.0 nm.

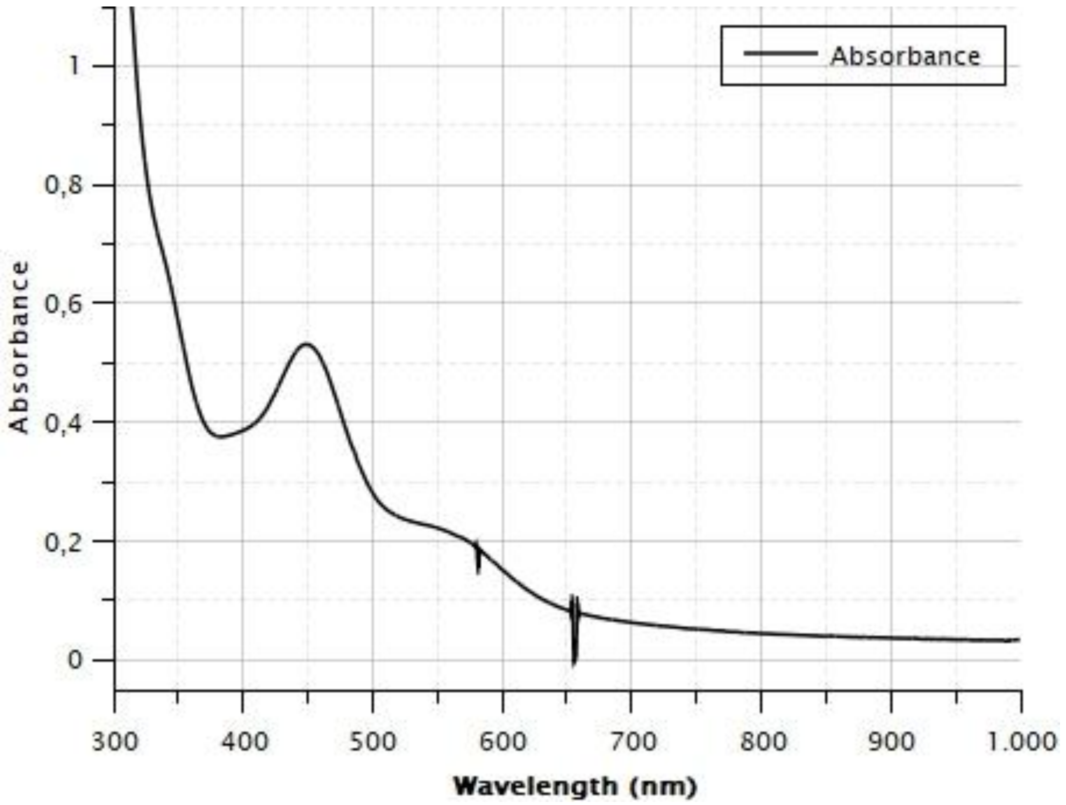

Figure S34: UV-Vis spectrum of $[Pt(PCy_3)_2(Bi(CH_2)_5)(SbF_6)]$ in dichloromethane ($c = 2.4 \cdot 10^{-4}$ mol/L), λ_{max} = 448.5, 568.5 nm.

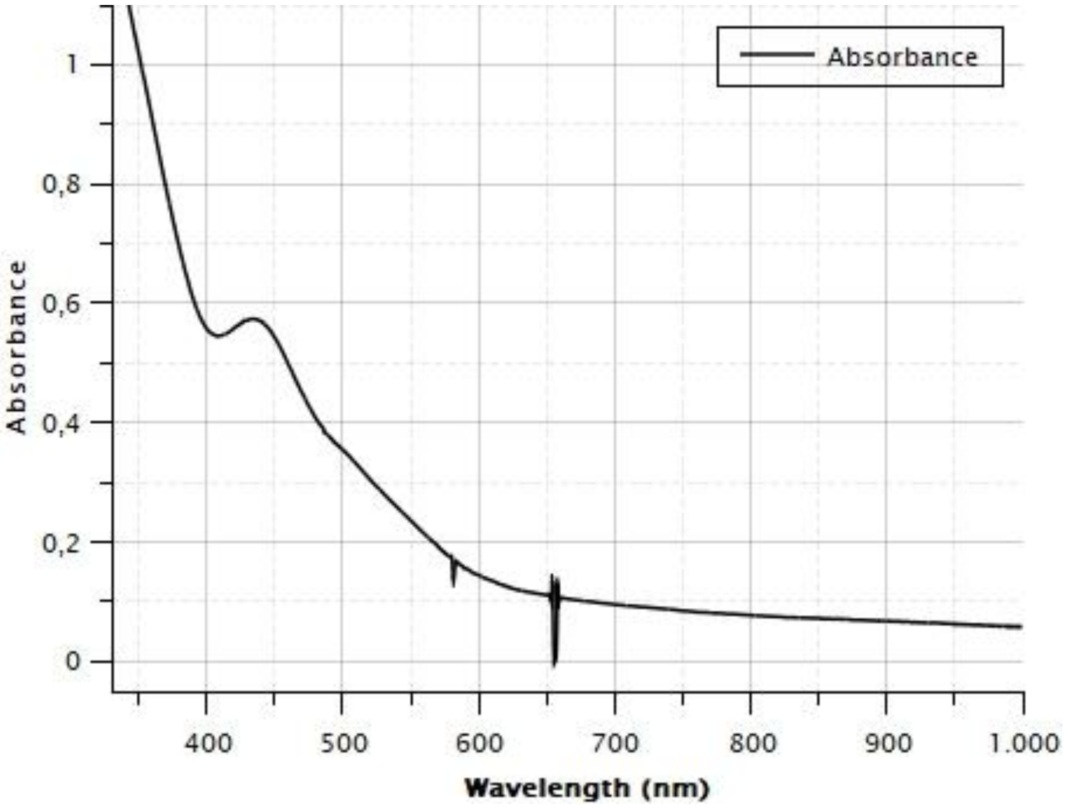

Figure S35: UV-Vis spectrum of $[Pt(PCy_3)_2(Bi\textit{c}Pr_2)(SbF_6)]$ in dichloromethane ($c = 4.6 \cdot 10^{-4}$ mol/L), λ_{max} = 435.5, (510.0) nm.

4. High Resolution Mass Spectra

Charge number:1
Element:^{12}C:0 .. 5, ^{1}H:0 .. 10, ^{209}Bi:0 .. 1, ^{79}Br:0 .. 1

Tolerance:5.00[ppm], 2.00 .. [mDa]

Unsaturation Number:-1.5 .. 50.0 (Fraction:Both)

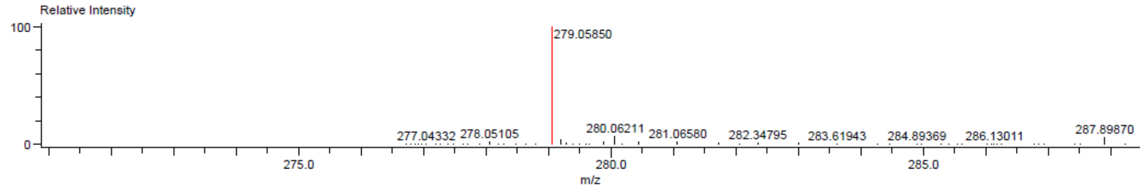

Composition:$C_5H_{10}Bi$
Mono Isotopic Mass:279.05863
Description:

Average Mass:279.11328

Created:19.07.2024 09:15:21
Nominal Mass:279
Created by:Lorakis

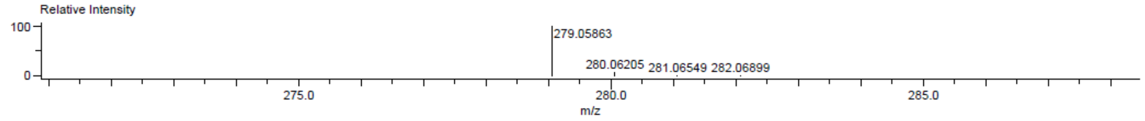

Mass	Intensity	Relative Intensity	Calc. Mass	Mass Difference [mDa]	Mass Difference [ppm]	Possible Formula	Unsaturation Number
279.05850	3519723.56	100.00	279.05863	-0.13	-0.48	$^{12}C_5{}^{1}H_{10}{}^{209}Bi_1$	1.5

Figure S36: Top: HRMS-CI mass spectrum of [Bi(CH_2)$_5$Br] enlarged at 279 m/z. Bottom: simulation of the signal at 279 m/z corresponding to [Bi(CH_2)$_5$]$^+$.

Data:231130_FD_270_Lb
Comment:
Description:
Ionization Mode:FD+
History:Average(MS[1] 3.87..3.90)-1*Average(MS[1] 4.30..4.48),#0-

Acquired:06.12.2023 12:02:34
Operator:AccuTOF
m/z Calibration File:FD_Calib_082222_b
Created:00:00:00
Created by:

Charge number:1
Element:^{12}C:0 .. 29, ^{1}H:0 .. 33, ^{79}Br:0 .. 2, ^{81}Br:0 .. 2, ^{56}Fe:0 .. 1, ^{14}N:0 .. 3, ^{16}O:0 .. 1

Tolerance:5.00[ppm], 2.00 .. [mDa]

Unsaturation Number:-1.5 .. 50.0 (Fraction:Both)

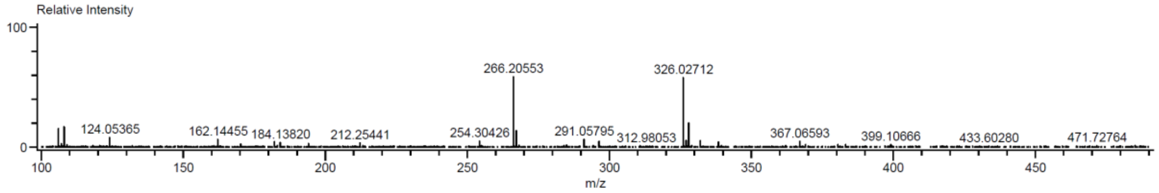

Figure S37: HRMS-LIFDI mass spectrum of [Bi(cPr)$_2$Cl] (**3-Cl**).

Data:231130_FD_270_Lb
Comment:
Description:
Ionization Mode:FD+
History:Average(MS[1] 3.87..3.90)-1*Average(MS[1] 4.30..4.48),#0-

Acquired:06.12.2023 12:02:34
Operator:AccuTOF
m/z Calibration File:FD_Calib_082222_b
Created:07.12.2023 15:18:27
Created by:Lorakis

Charge number:1
Element:^{12}C:0 .. 6, ^{1}H:0 .. 10, ^{209}Bi:0 .. 1, ^{35}Cl:0 .. 1

Tolerance:5.00[ppm], 2.00 .. [mDa]

Unsaturation Number:-1.5 .. 50.0 (Fraction:Both)

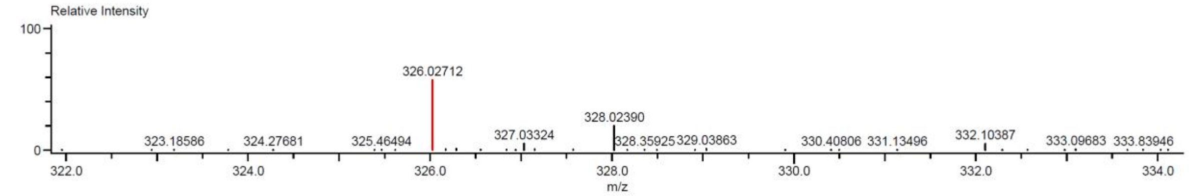

Composition:$C_6H_{10}BiCl$
Mono Isotopic Mass:326.02749
Description:

Average Mass:326.57668

Created:07.12.2023 15:18:29
Nominal Mass:326
Created by:Lorakis

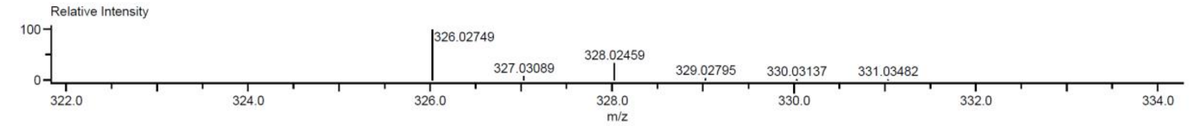

Mass	Intensity	Relative Intensity	Calc. Mass	Mass Difference [mDa]	Mass Difference [ppm]	Possible Formula	Unsaturation Number
326.02712	17502.97	57.90	326.02749	-0.37	-1.13	$^{12}C_6{}^{1}H_{10}{}^{209}Bi_1{}^{35}Cl_1$	2.0

Figure S38: Top: HRMS-LIFDI mass spectrum of [Bi(cPr)$_2$Cl] (**3-Cl**) enlarged at 326 m/z. Bottom: simulation of the signal at 326 m/z corresponding to [Bi(cPr)$_2$Cl]$^+$.

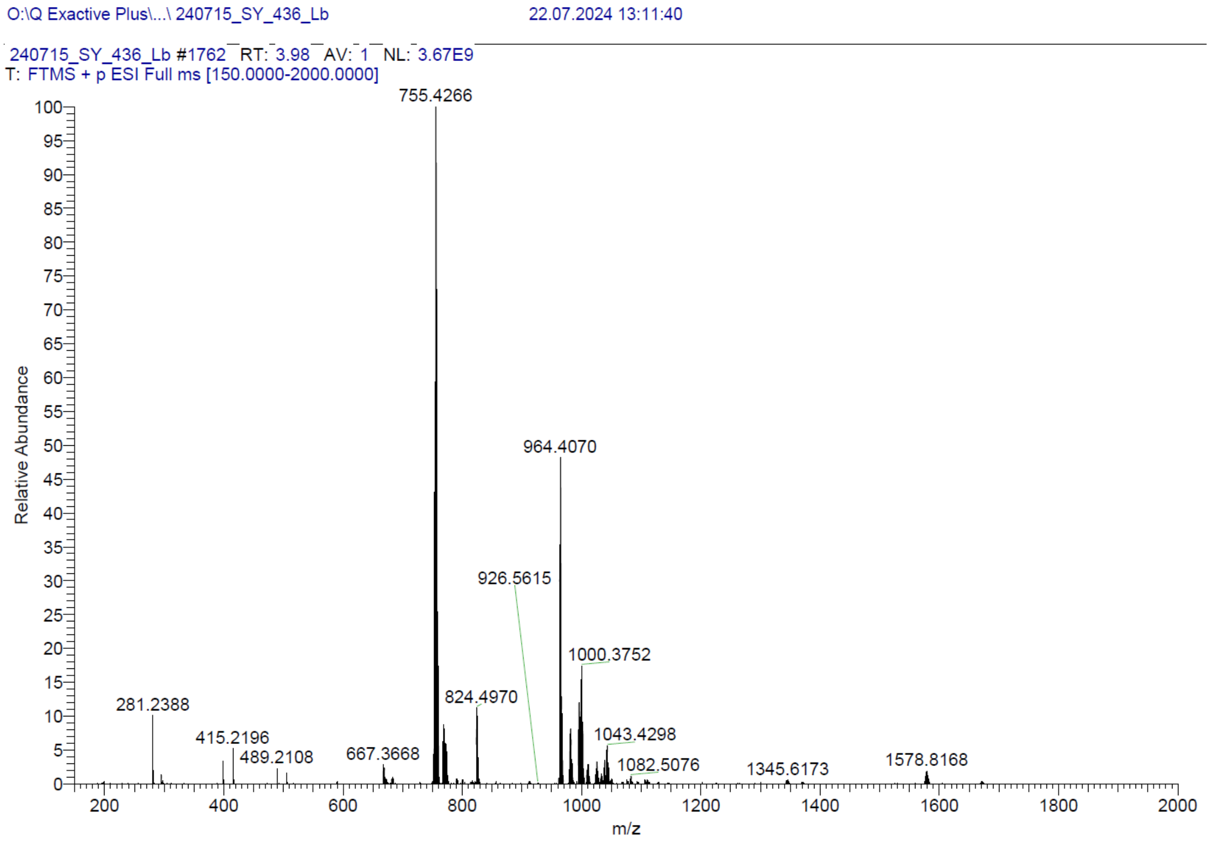

Figure S39: HRMS-ESI mass spectrum of [Pt(PCy$_3$)$_2$(BiiPr$_2$)(SbF$_6$)].

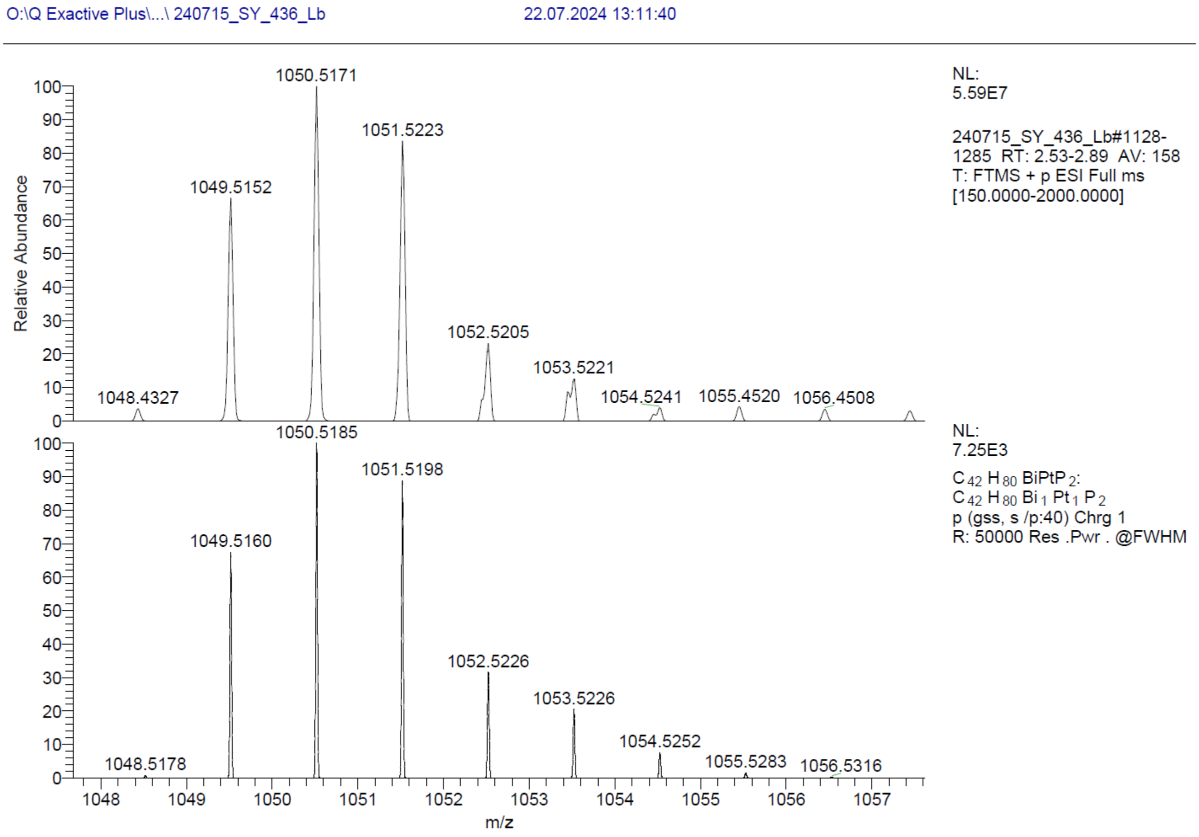

Figure S40: Top: HRMS-ESI mass spectrum of [Pt(PCy$_3$)$_2$(Bi(*i*Pr)$_2$)(SbF$_6$)] enlarged at 1050 m/z. Bottom: Simulation of the Signal at 1050 m/z corresponding to [Pt(PCy$_3$)$_2$(Bi(*i*Pr)$_2$)]$^+$.

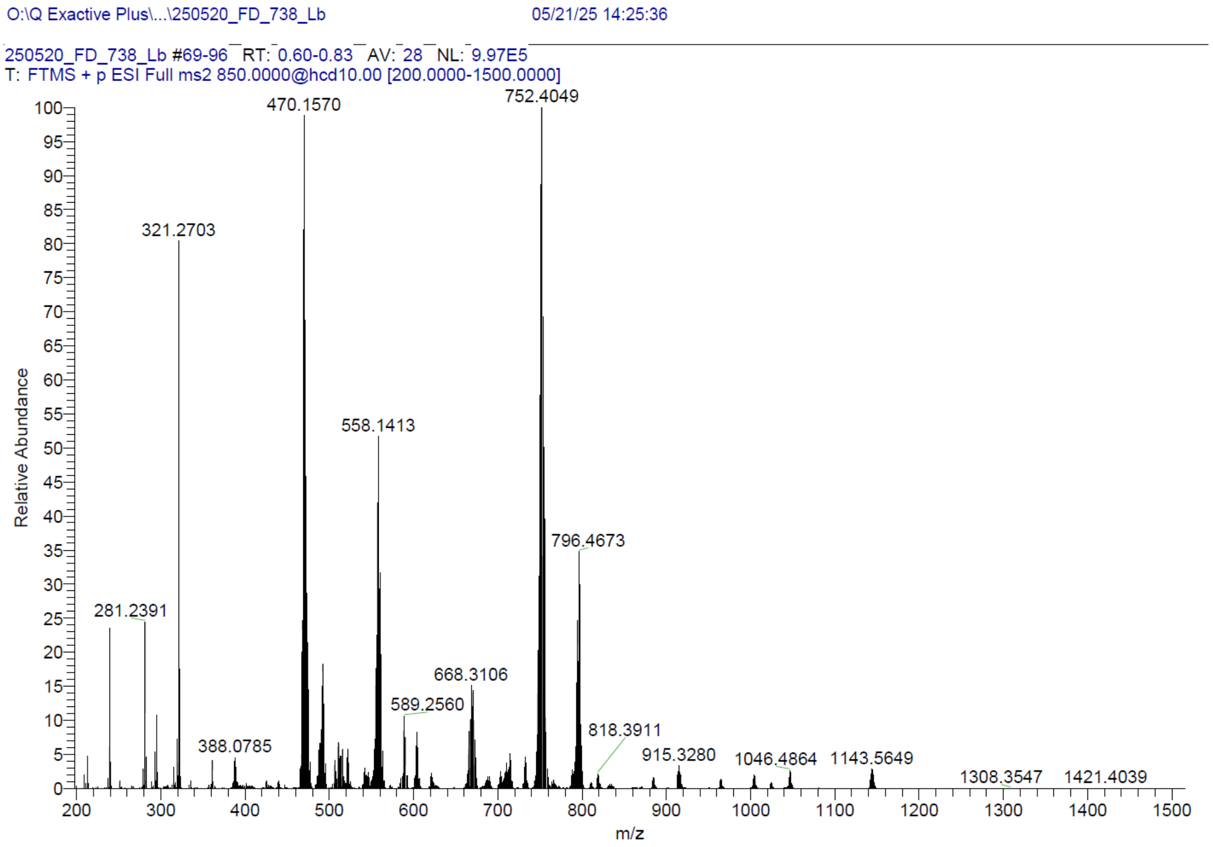

Figure S41: HRMS-LIFDI mass spectrum of [Pt(PCy$_3$)$_2$(Bi*c*Pr$_2$)(SbF$_6$)].

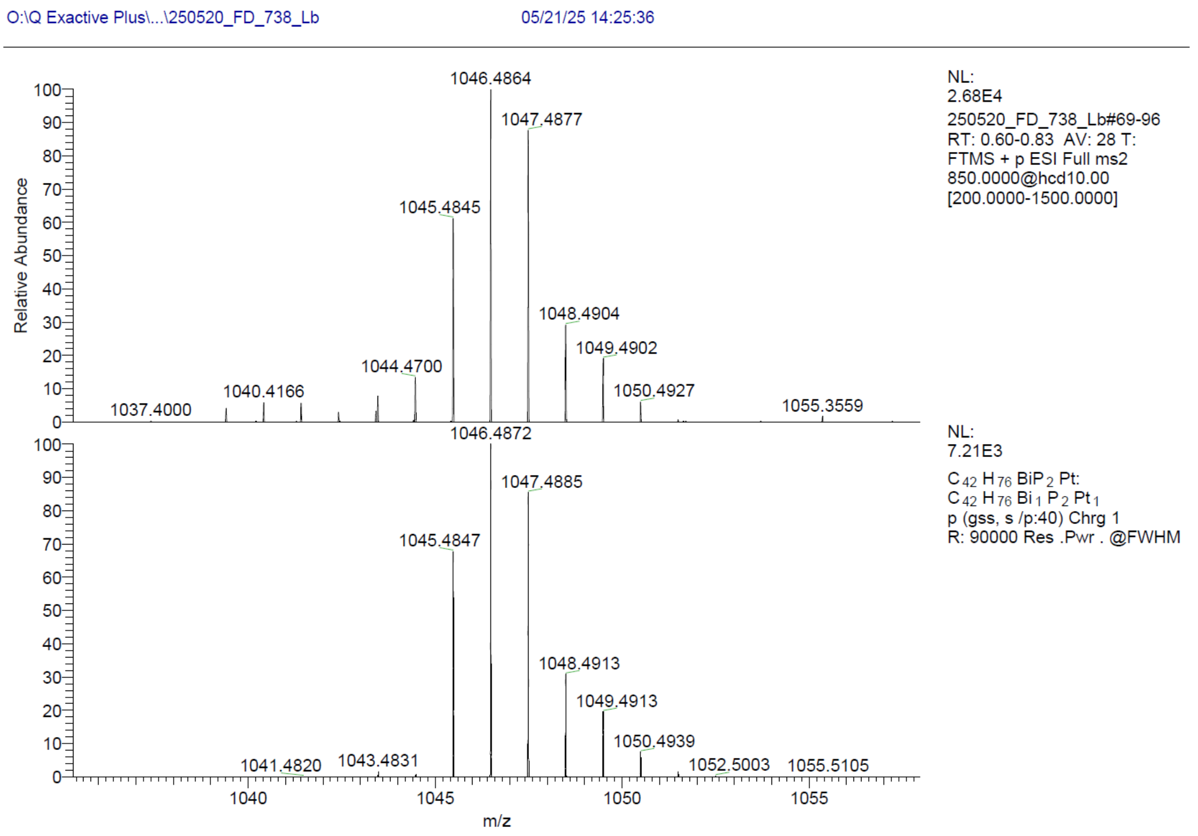

Figure S42: Top: HRMS-LIFDI mass spectrum of [Pt(PCy$_3$)$_2$(Bi*c*Pr$_2$)(SbF$_6$)] enlarged at 1046 m/z. Bottom: simulation of the signal at 1046 m/z corresponding to [Pt(PCy$_3$)$_2$(Bi*c*Pr$_2$)]$^+$.

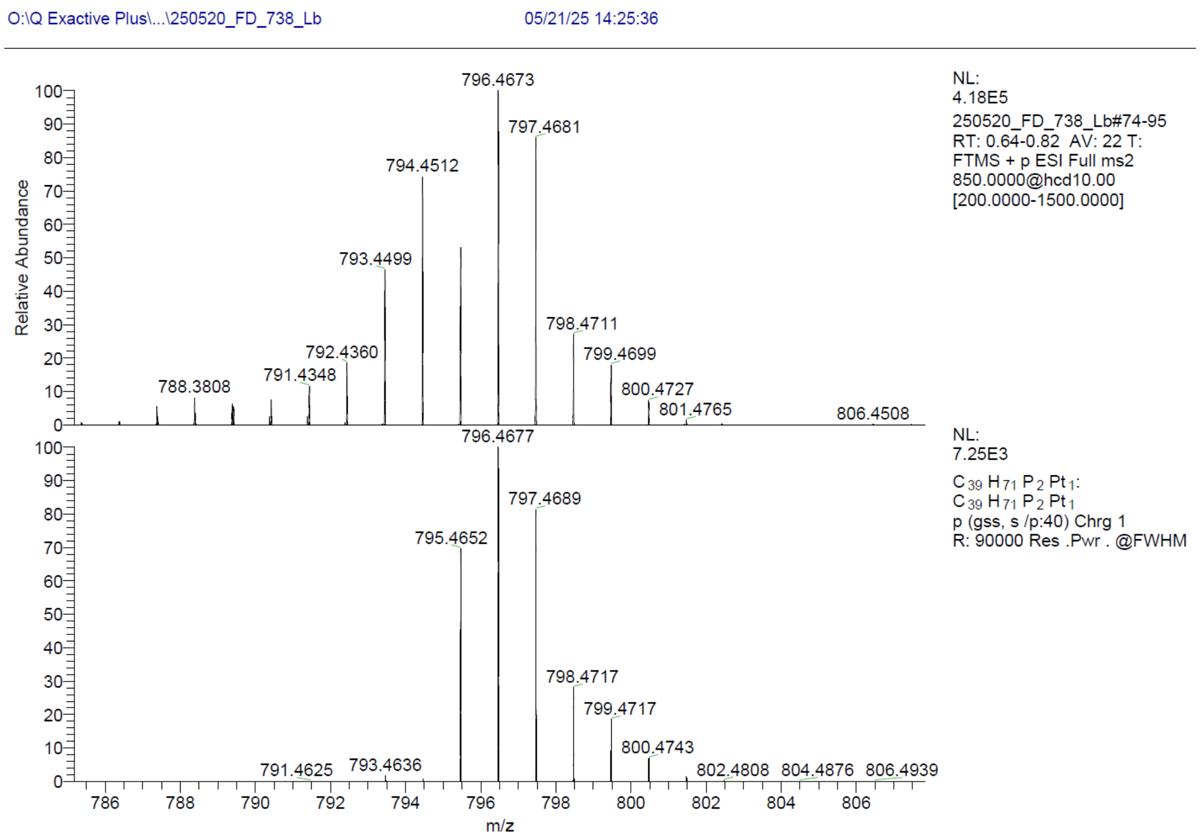

Figure S43: Top: HRMS-LIFDI mass spectrum of [Pt(PCy$_3$)$_2$(Bi*c*Pr$_2$)(SbF$_6$)] enlarged at 796 m/z. Bottom: simulation of the signal at 796 m/z corresponding to [Pt(PCy$_3$)$_2$(η^3-C_3H_5)]$^+$.

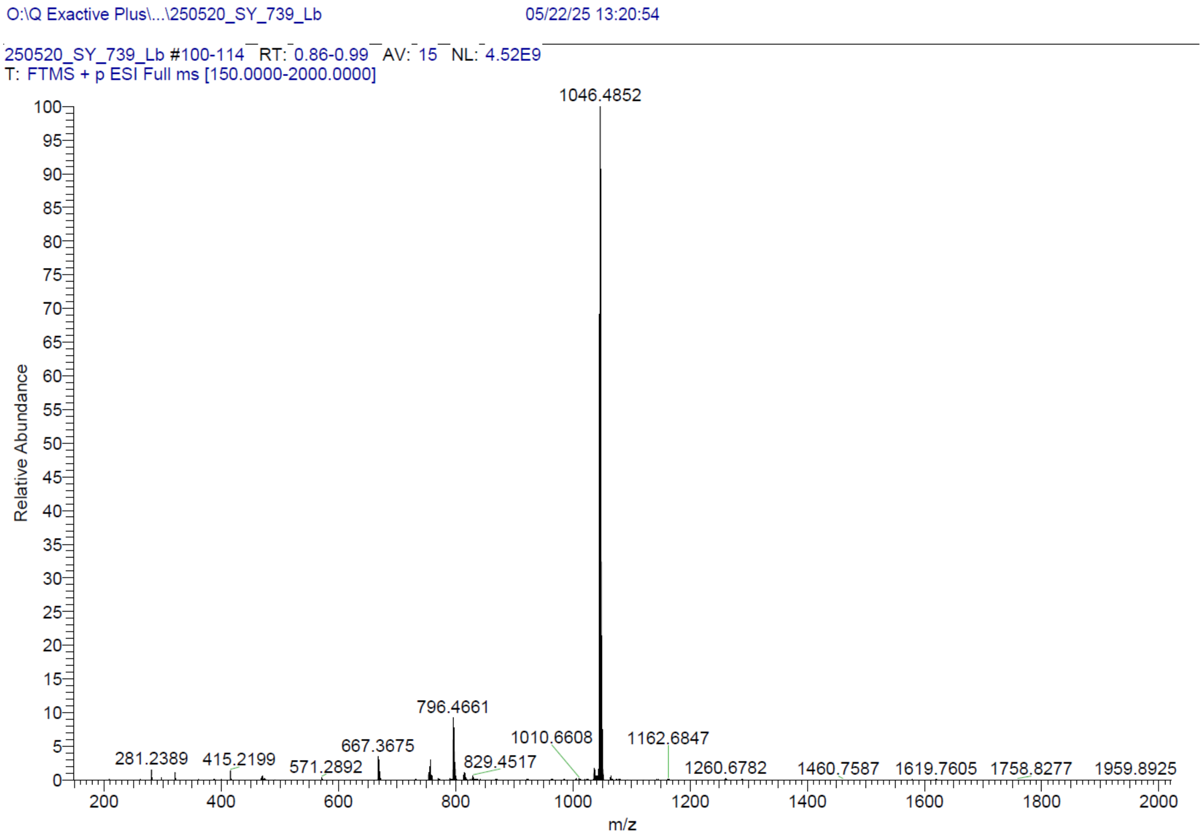

Figure S44: HRMS-ESI mass spectrum of [Pt(PCy$_3$)$_2$(Bi*c*Pr$_2$)(SbF$_6$)].

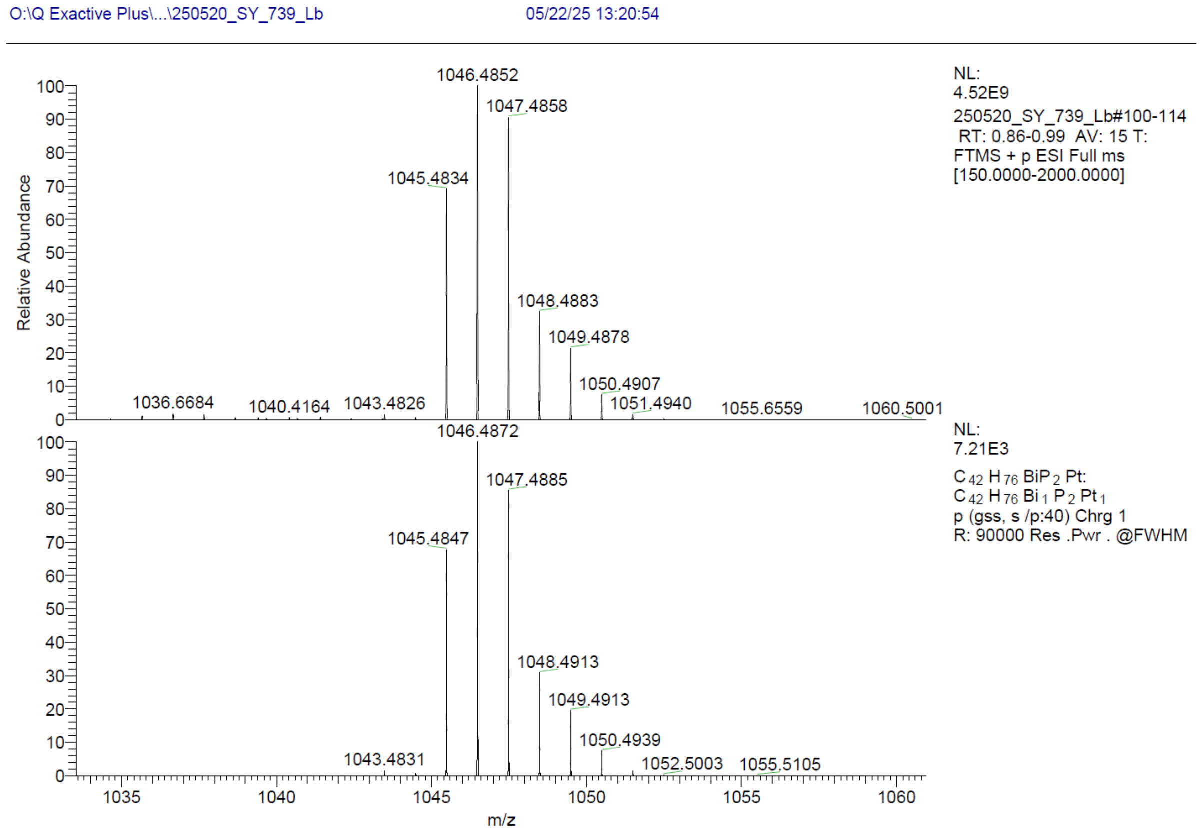

Figure S45: Top: HRMS-ESI mass spectrum of [Pt(PCy$_3$)$_2$(Bi*c*Pr$_2$)(SbF$_6$)] enlarged at 1046 m/z. Bottom: simulation of the signal at 1046 m/z corresponding to [Pt(PCy$_3$)$_2$(Bi*c*Pr$_2$)]$^+$.

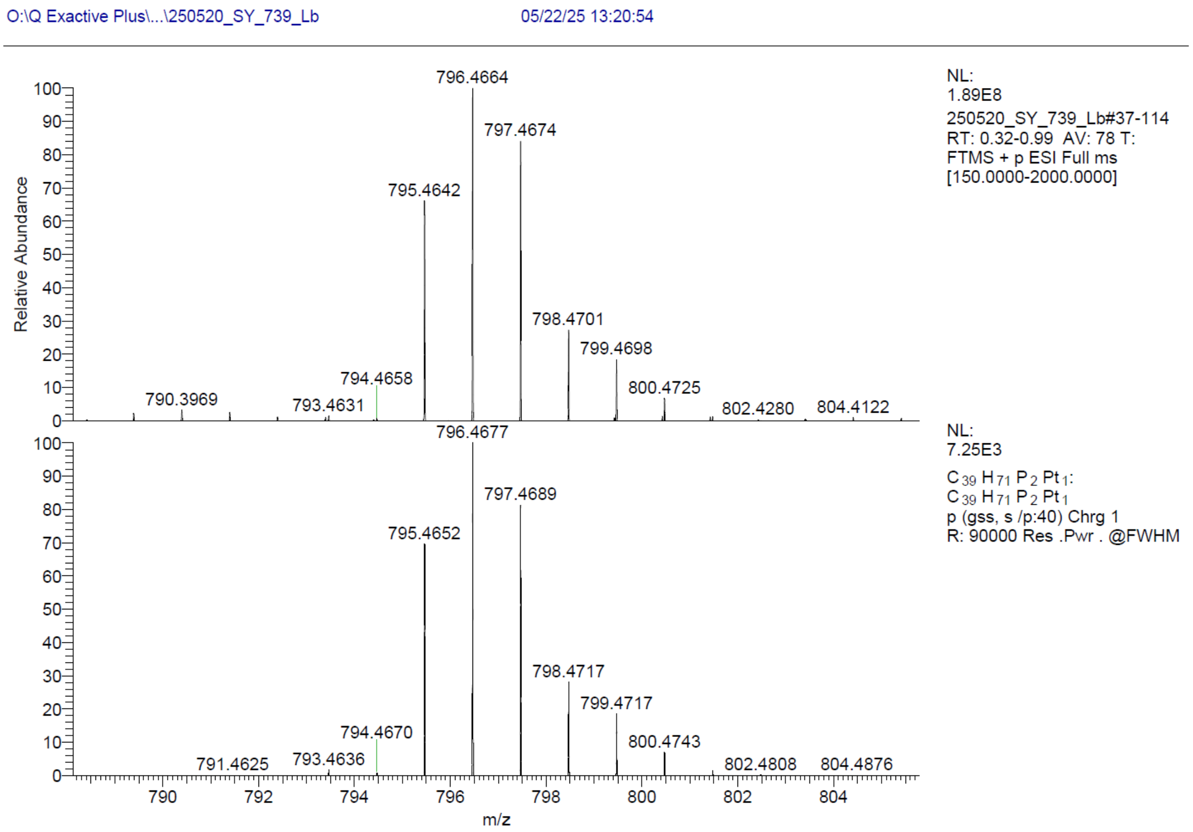

Figure S46: Top: HRMS-ESI mass spectrum of [Pt(PCy_3)$_2$(Bi*c*Pr_2)(SbF_6)] enlarged at 796 m/z. Bottom: simulation of the signal at 796 m/z corresponding to [Pt(PCy_3)$_2$(η^3-C_3H_5)]$^+$.

5. IR Spectra

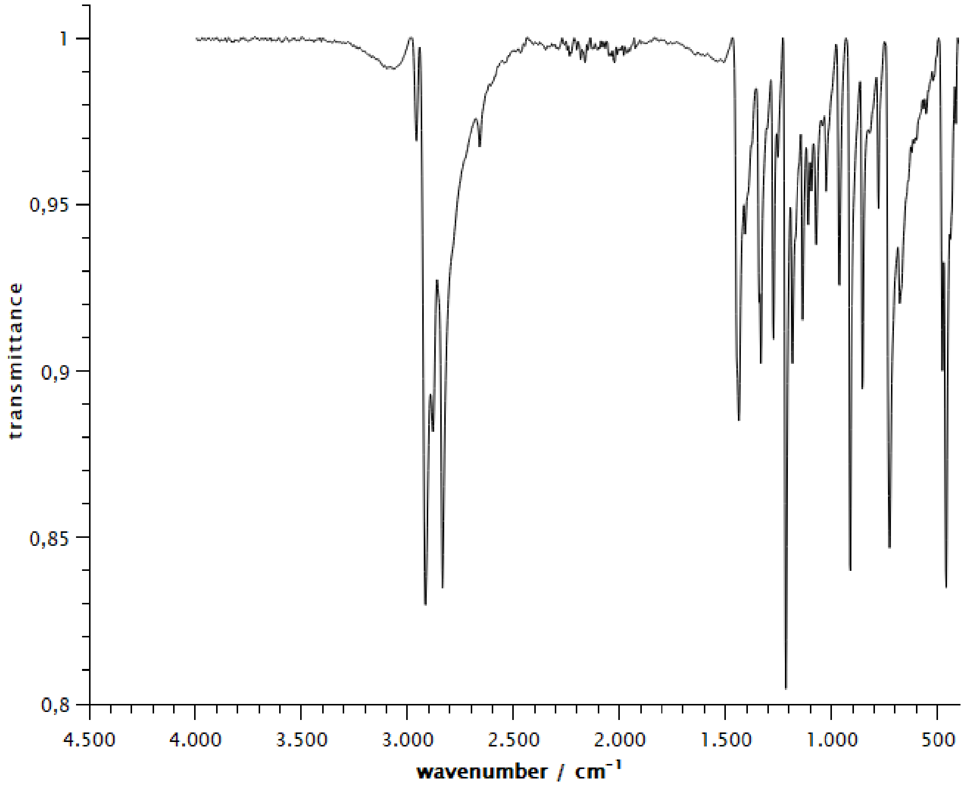

Figure S47: IR spectrum of [Bi($(CH_2)_5$Br] (neat).

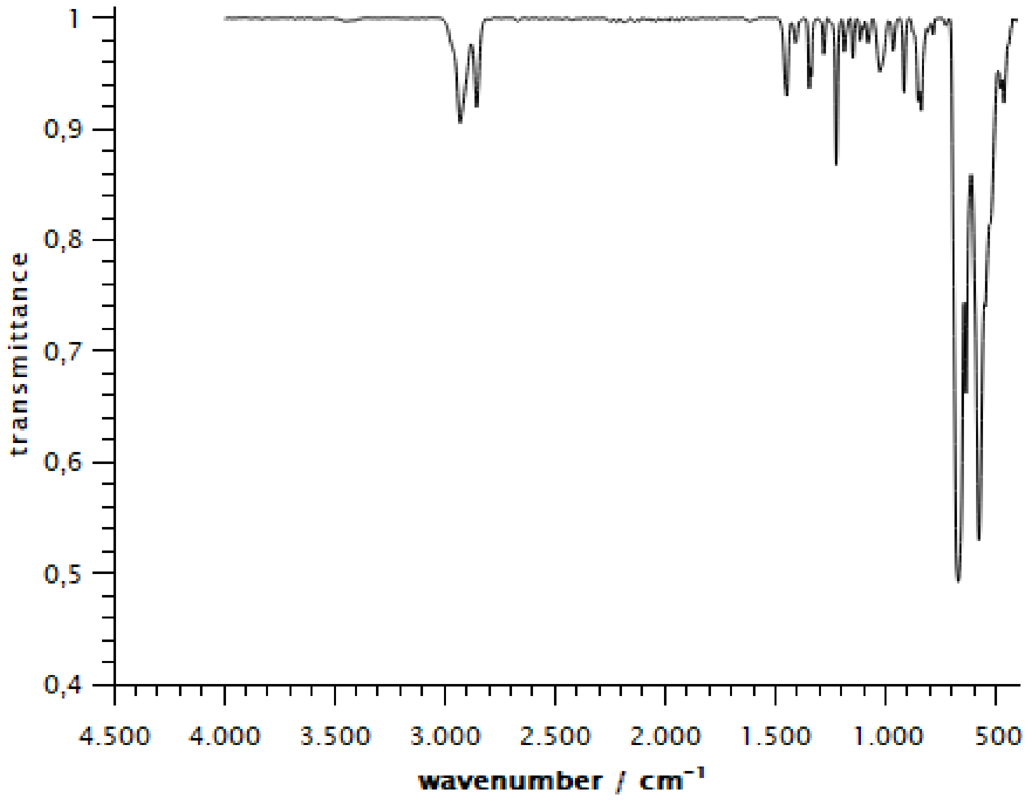

Figure S48: IR spectrum of [Bi$(CH_2)_5$(SbF_6)] (neat).

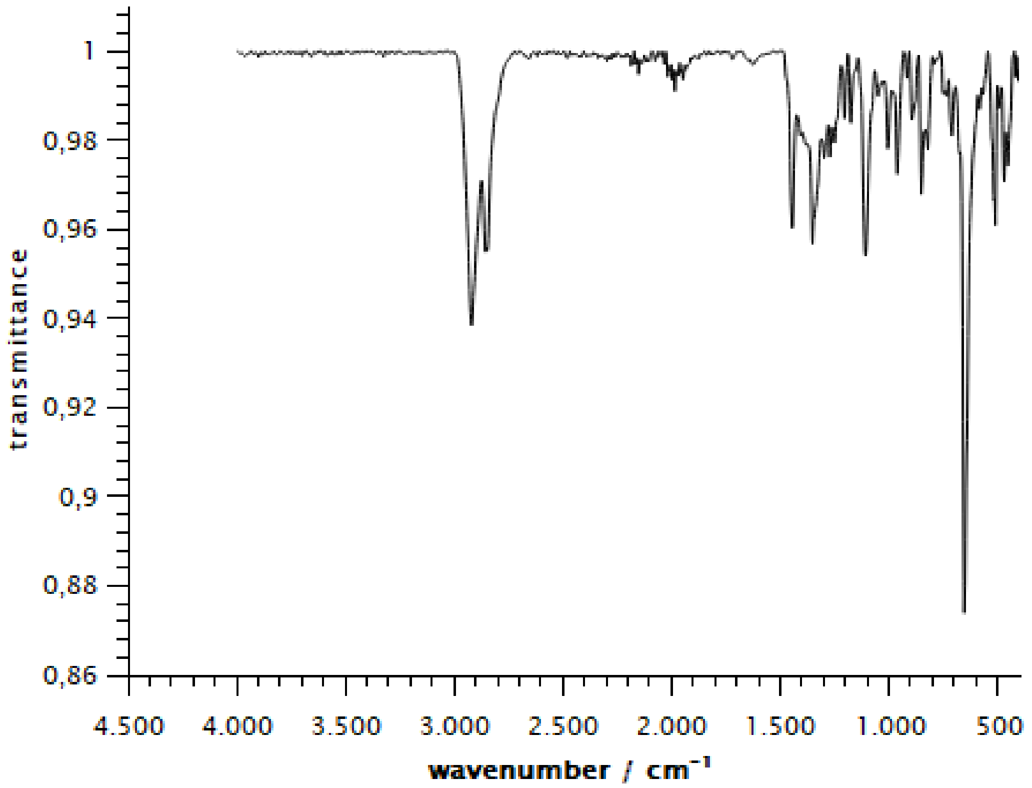

Figure S49: IR spectrum of [Pt(PCy_3)$_2$Bi(CH_2)$_5$(SbF_6)] (neat).

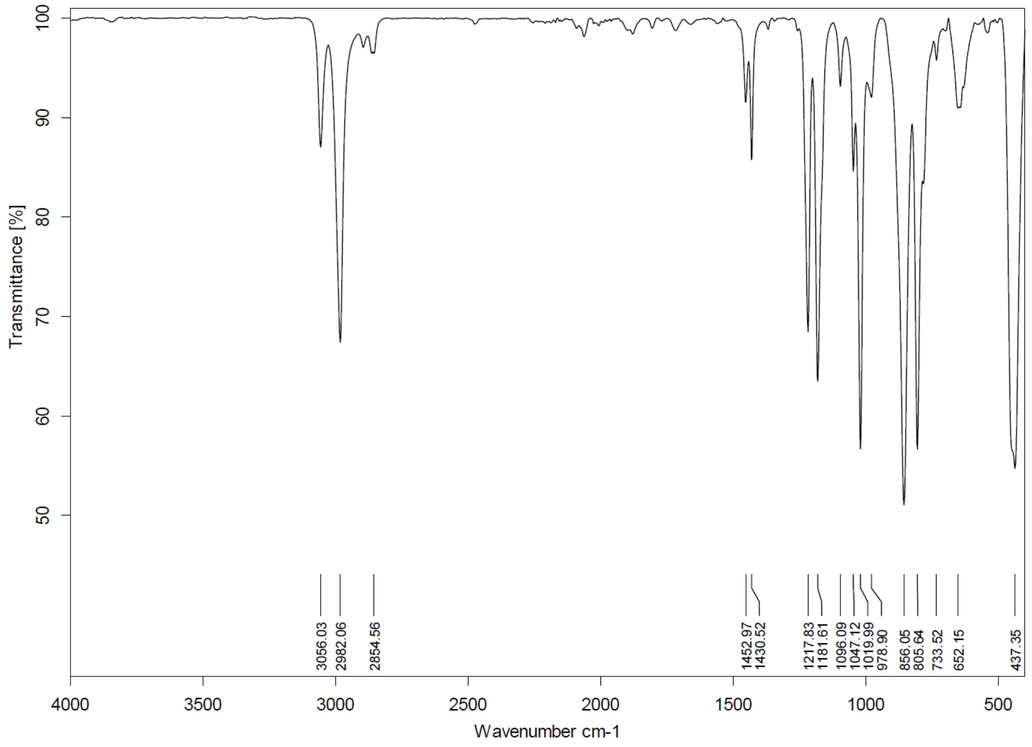

Figure S50: IR spectrum of [Bi(*c*Pr)$_3$] (neat).

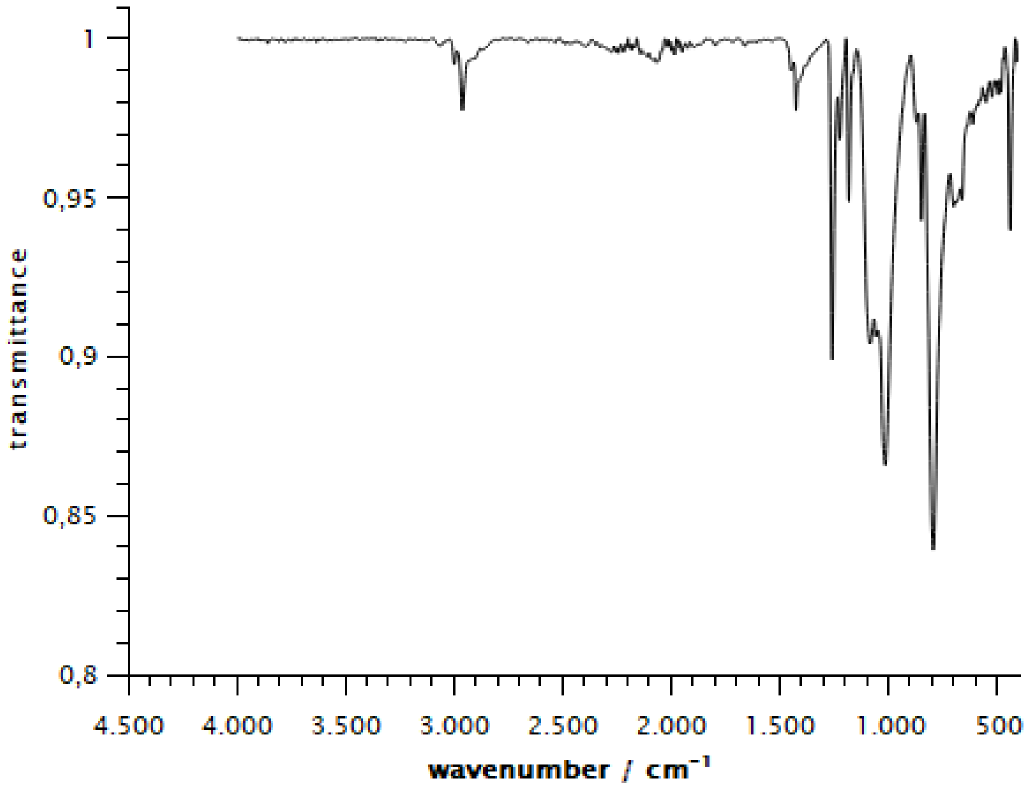

Figure S51: IR spectrum of [Bi(*c*Pr)Cl_2] (neat).

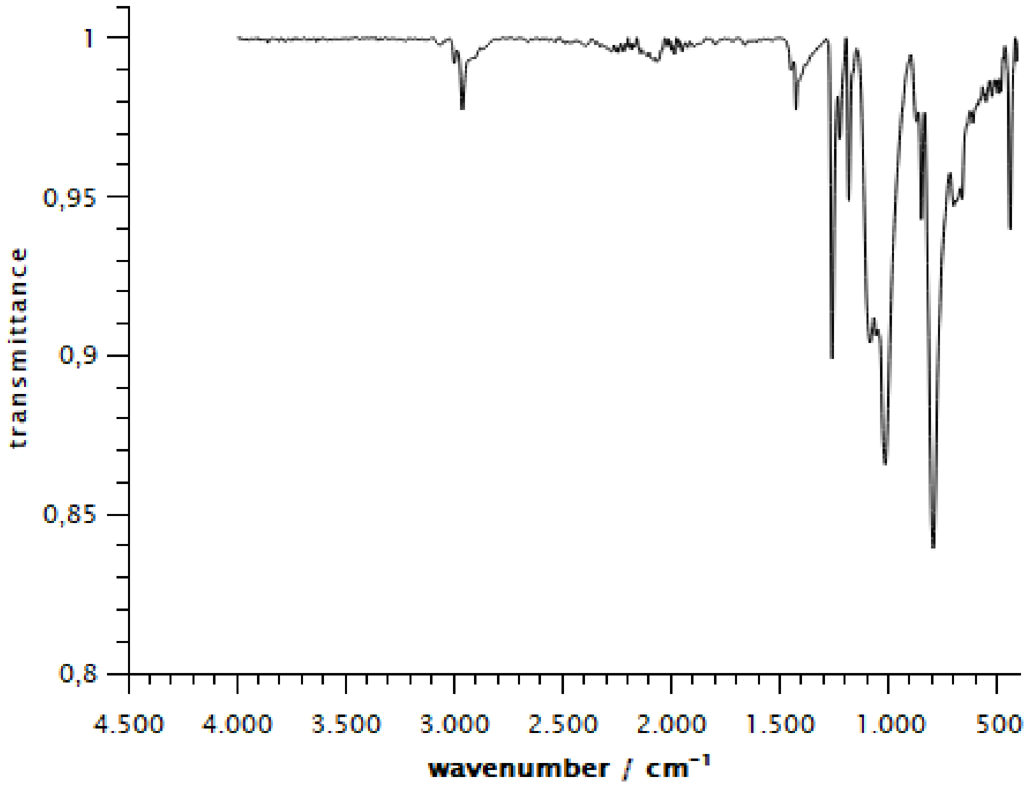

Figure S52: IR spectrum of [Bi(*c*Pr)$_2$Cl] (DCM).

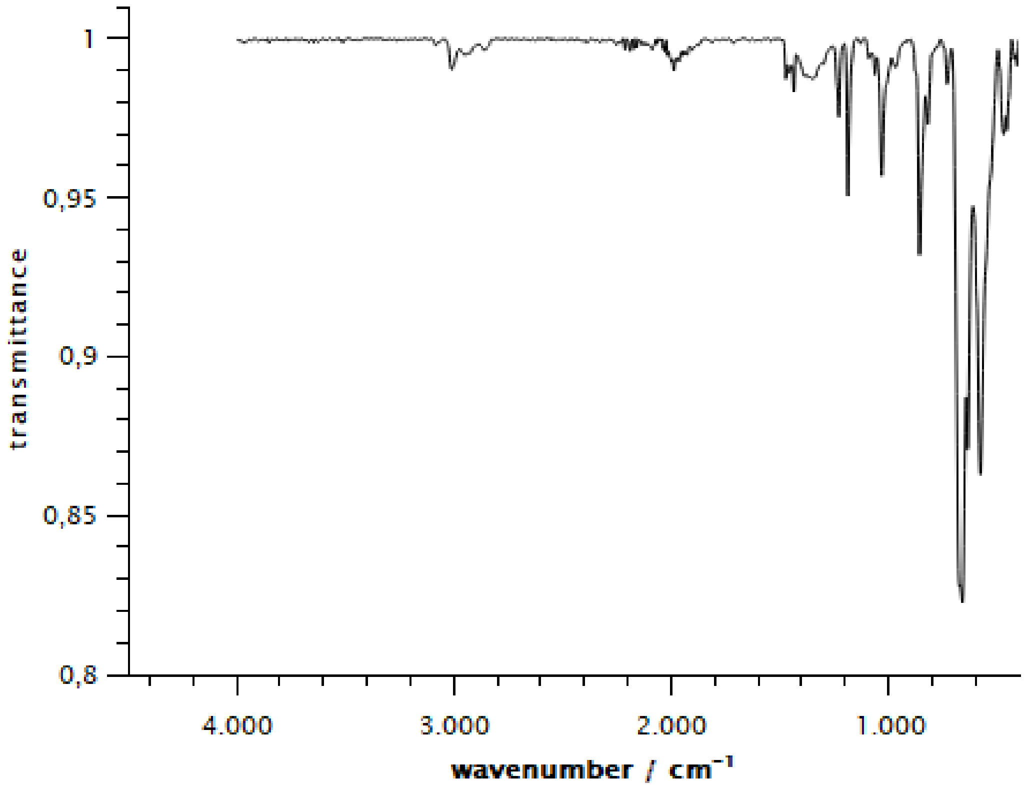

Figure S53: IR spectrum of [Bi(cPr)$_2$(SbF$_6$)] (neat).

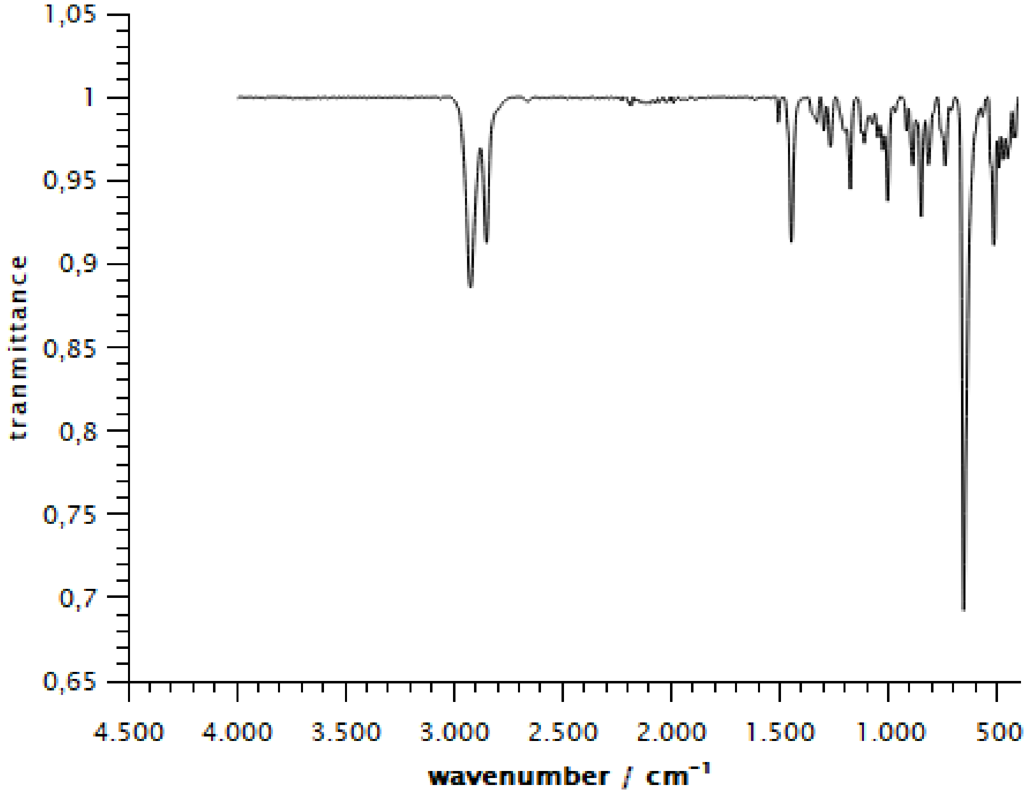

Figure S54: IR spectrum of [Pt(PCy$_3$)$_2$Bi(cPr)$_2$(SbF$_6$)] (neat).

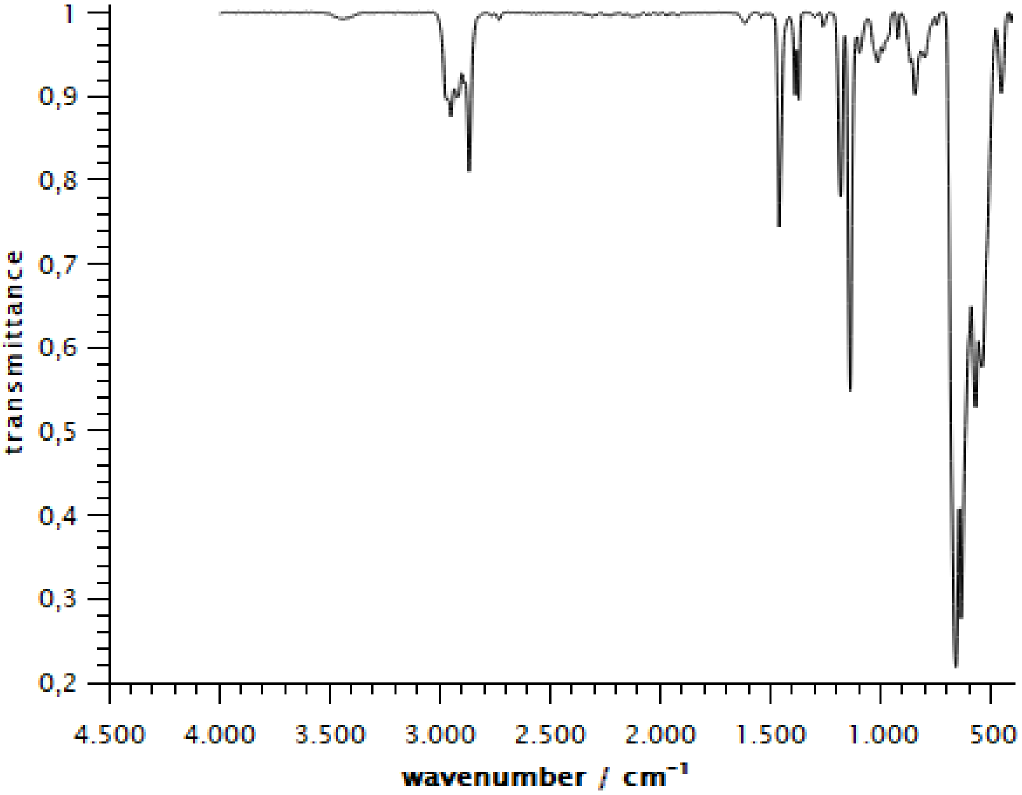

Figure S55: IR spectrum of [Bi(*i*Pr)$_2$(SbF$_6$)] (neat).

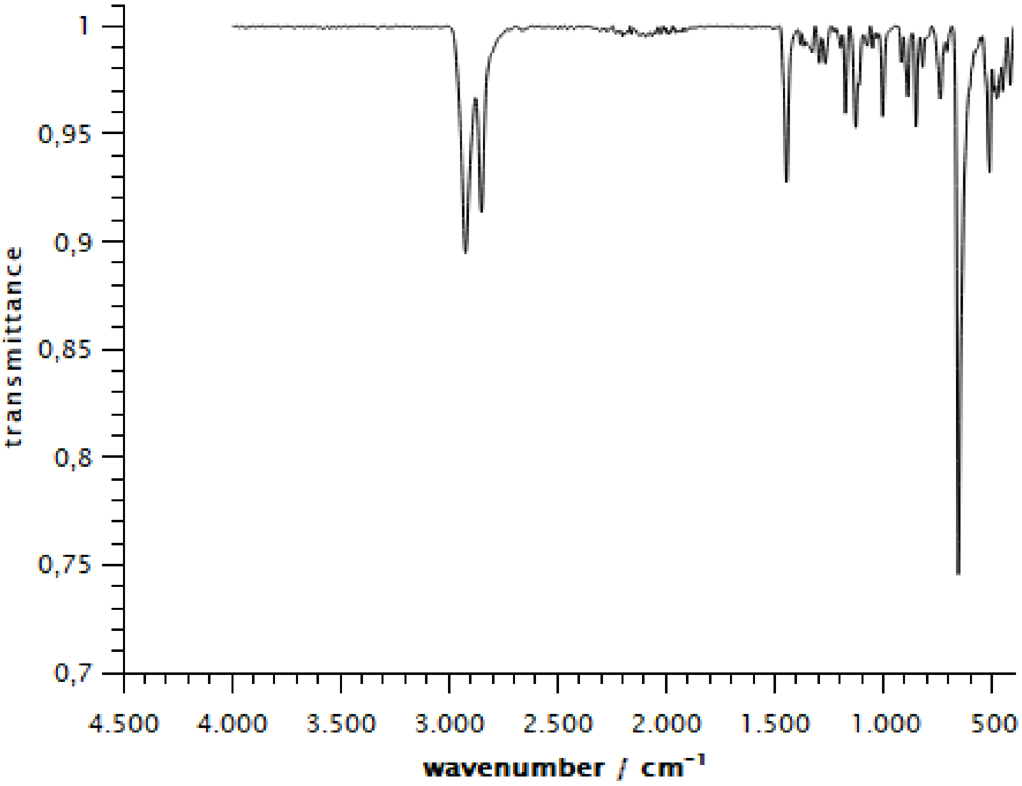

Figure S56: IR spectrum of [Pt(PCy$_3$)$_2$Bi(*i*Pr)$_2$(SbF$_6$)] (neat).

6. Structural characterization of $BiC_5H_{10}Br$ (1)

The bismuth atoms in compound **1** exhibit a distorted bisphenoidal coordination polyhedron, with the bromine atoms occupying the axial positions. The Br–Bi–Br angles (170.02(5)° and 173.56(6)°) are smaller than the ideally expected angle of 180°. The C–Bi–C angles range from 92.1(7)° to 93.5(7)°, reflecting the involvement of two p(Bi) orbitals in Bi–C bond formation and geometric constraints due to the cyclic nature of the structural motif. There are four independent molecules in the unit cell, which assemble into a polymeric chain (Figure S48a). The polymeric chain forms a helical structure in which the organic residues are always oriented outward (Figure S48b). The chains run parallel in an offset arrangement, resulting in interchain Bi–Br distances of 3.827 - 3.893 Å, both of which are slightly below the sum of the van der Waals radii of bismuth and bromine (3.92 Å),[65,66] suggesting weak interactions.

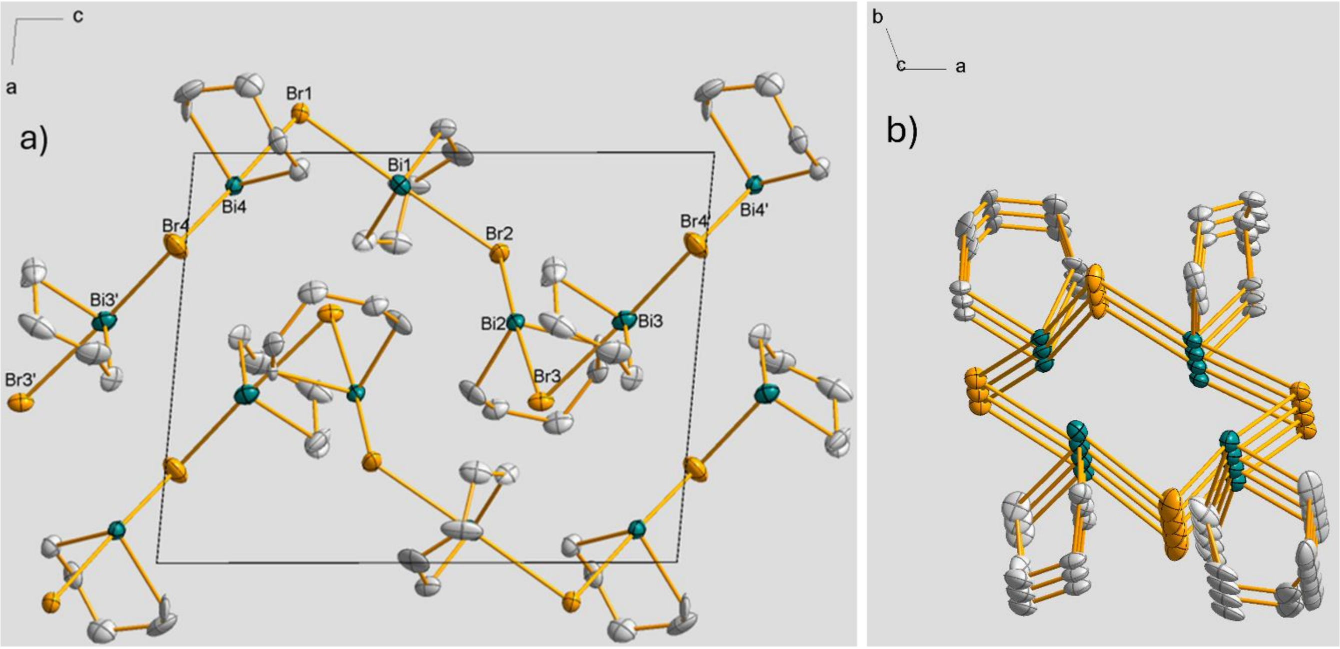

Figure S57: Molecular structure of $Bi(CH_2)_5Br$ (**1**) view along the *b* axis (a) and along the *c* axis (b), displacement ellipsoids are drawn at 50% probability level, hydrogen atoms omitted. Selected bond lengths [Å] and angles [°]: Bi1–Br1: 2.9614(18),3.0191(18), Bi1–Br2: 2.8956(19), Bi2–Br2: 2.8534(19), Bi2–Br3: 2.9797(19), Bi3–Br3: 2.999(2), Bi3–Br4: 2.865(2), Bi4–Br1: 3.0191(18), Bi4–Br4: 2.866(2), Bi1–C1: 2.239(15), Bi2–C5: 2.263(16), Bi2–C6: 2.247(17), Bi2–C10: 2.253(16), Bi3–C11: 2.242(19), Bi3–C15: 2.23(2), Bi4–C16: 2.235(18), Bi4–C16: 2.257(19), Br1–Bi1–Br2: 170.02(5), Br2–Bi2–Br3: 173.36(6), Br3–Bi3–Br4: 171.59(6), Br1–Bi4–Br4: 173.56(6), Bi1–Br1–Bi4: 91.60(5), Bi1–Br2–Bi2: 111.93(6), Bi2–Br3–Bi3: 89.79(5), Bi3–Br4–Bi4: 104.79(8), C1–Bi1–C5: 93.5(7), C6–Bi2–C10: 92.3(6), C11–Bi3–C15: 92.7(8), C16–Bi4–C20: 92.1(7).

7. Structural characterization of [Bi(*c*Pr)Cl_2]

Since Bi(*c*Pr)$_3$ and Bi(*c*Pr)$_2$Cl proved to be liquids even at –30 °C, the first molecular structure that confirmed a cyclopropyl group attached to bismuth was the one of Bi(*c*Pr)Cl_2 (**3-Cl2**) (monoclinic space group *P2*$_1$*/n*, Z = 4). Compound **3-Cl2** it forms a complex two-dimensional coordination polymer in the solid state (see Figure S 33 b) and c)). The monomer shows a trigonal pyramidal coordination polyhedron for the bismuth atom, with two chloride atoms and one cyclopropyl ligand.

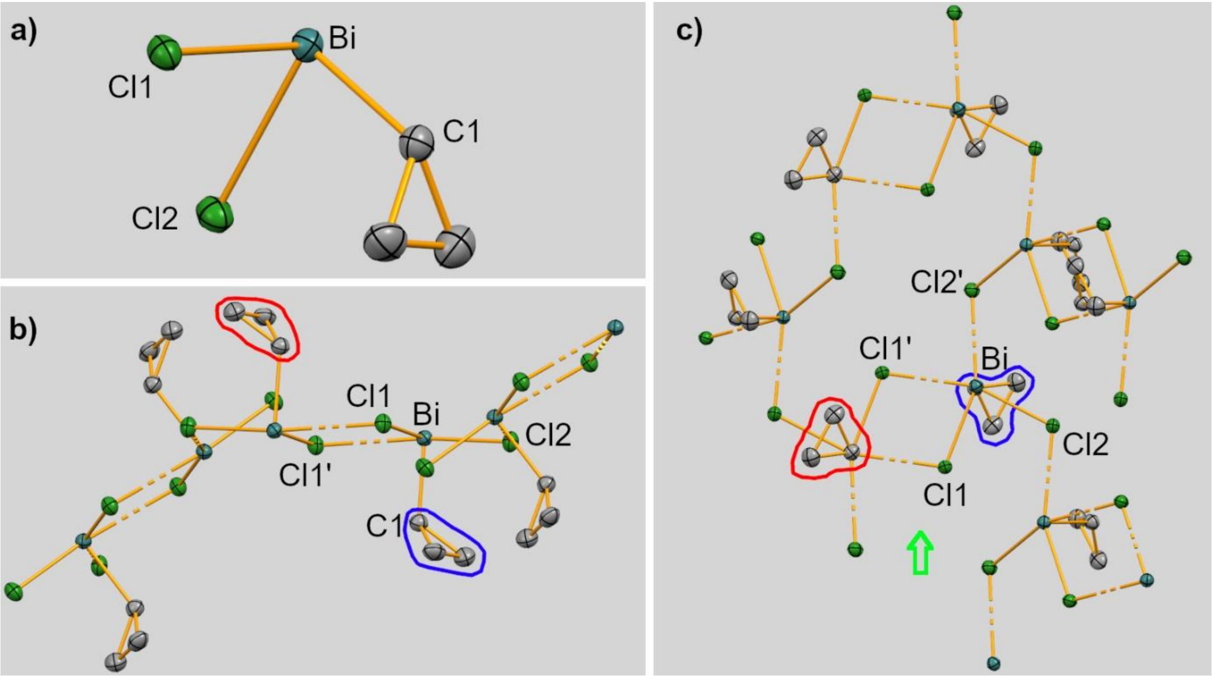

Figure S58: Molecular structure of Bi(*c*Pr)Cl_2, displacement ellipsoids are drawn at 50% probability level, hydrogen atoms are omitted for clarity. a) Monomer unit. b) side view of the coordination polymer. c) top down view of the coordination polymer, green arrow marks the view axis in b), red and blue circles mark corresponding *c*Pr-groups in b) and c). selected bond lengths [Å] and angles: Bi–C1 2.211(4), Bi–Cl1 2.6378(9), Bi–Cl2 2.6212(9), Bi–Cl1' 2.8985(9), Bi–Cl2' 2.9773(10), C1–Bi–Cl1 92.83(11), Cl1–Bi–Cl2 83.72(3), Cl1–Bi–Cl1' 77.69(3), Cl2–Bi–Cl2' 119.05(3).

The Bi–C bond length amounts to 2.211(4) Å, which is in line with other compounds showing Bi–C(sp^3) bonds, like BiMeCl_2 (2.225(12) Å).[85] Since the *cyclo*propyl group has been argued to show properties ranging between those of regular alkyl- and aryl-groups, a comparison with the structurally characterized BiDipp$_2$Cl may be envisaged. This compound shows a Bi–C bond length of 2.268(4) A,[86] suggesting that steric factors are non-negligible. The chloride ligands are found within a distance of 2.6378(9) Å and 2.6212(9) Å from the central bismuth atom in Bi(*c*Pr)Cl_2. In BiMeCl_2 the bond lengths are 2.7411(14) Å and 2.7553(13) Å, whereas in BiDippCl_2 2.5430(11) Å and 2.5525(11) Å are found, leaving the analyzed Bi(*c*Pr)Cl_2 in the middle of the two extremes. Apart from the organic ligand at the bismuth atom the formation of two-dimensional coordination polymers in the Cl–Bi–Cl plane has an influence on the Bi–Cl bond lengths due to coordination in *trans* position, donating electron density into the Bi–Cl σ* orbitals. For Bi(*c*Pr)Cl_2 these coordinating chlorine atoms are found in a distance of 2.8985(9) Å and 2.9773(10) Å. Due to the symmetry of the crystal structure of BiMeCl_2, these chlorine atoms are within

the same distance as the ones mentioned above (2.7411(14) Å and 2.7553(13) Å). $BiDippCl_2$ exhibits the largest dative Bi–Cl bonds in this scope with 3.0771(10) Å and 3.1901(10) Å. Again, Bi(*c*Pr)Cl_2 lies in between the two references, this can be rationalized by the steric demand as well as the electronic influence of the organic substituents.

8. Structural characterization of [Pt(PCy_3)$_2$(η^3-C_3H_5)][SbF_6] (10)

The coordination geometry around the platinum atom is best described as trigonal planar with two phosphine ligands and one η^3-C_3H_5 unit. The relevant bond angles are close to 120 ° when a primary bonding interaction along the Pt–C2 axis is assumed (P1–Pt–P2: 111.00(2) °, C2–Pt–P1 120.92(12) °, C2–Pt–P2 125.80(12) °). Otherwise, it could also be described as a distorted square planar coordination geometry, where the phosphine ligands are in *cis* configuration, with C1–Pt–P1 and C3–Pt–P2 angles of 93.14(7) ° and 91.29(7) ° respectively. The SbF_6^- anion does not coordinate to the platinum center, implying no directional bonding interactions between the complex cation and the complex anion. The Pt–P bond lengths amount to 2.3289(6) Å and 2.3216 Å, while the Pt–C bond lengths range from 2.187(4) Å to 2.207(2) Å. All bonding parameters are not significantly different within three sigma limits from the isostructural compound [Pt(PCy_3)$_2$(η^3-C_3H_5)(PF_6)] (P1–Pt–P2 111.2 °, C–Pt–P 89.6(4) ° and 91.0(4) °, Pt–P 2.335(3) Å and 2.327(4) Å, Pt–C 2.16(5) Å to 2.24(2) Å),[87] implying that the counter ion does not have a significant influence on the bonding situation of the platinum complex. The data is also in line with other similar η^3-C_3H_5 platinum compounds.[88-92]

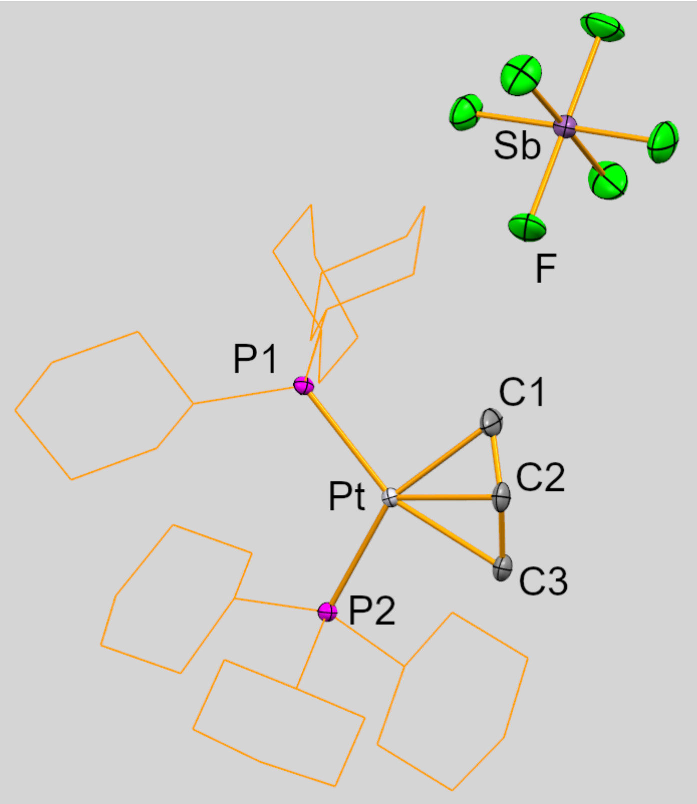

Figure S59: Molecular structure of [Pt(PCy_3)$_2$(η^3-C_3H_5)][SbF_6], displacement ellipsoids are drawn at 50% probability level, hydrogen atoms omitted and cyclohexyl groups shown as wireframe for clarity. Selected bond lengths [Å] and angles [°]: Pt–P1 2.3289(6), Pt–P2 2.3216(6), Pt–C1 2.199(3), Pt–C2 2.187(4), Pt–C3 2.207(2), P1–Pt–P2 111.00(2), P1–Pt–C1 91.34(7), P1–Pt–C2 120.92(12), P1–Pt–C3 157.64(7), P2–Pt–C3 91.29(7), C1–Pt–C3 66.55(10).

9. Single-crystal X-ray diffraction

Compound	**1**	**4**
Empirical formula	$4 \cdot BiC_5H_{10}Br$	$C_{10}H_{20}Bi_2F_{12}Sb_2$
Formula weight	1436.08	1029.72
Crystal color, habit	colorless, block	orange, block
Temperature/K	100	100
Crystal system	triclinic	monoclinic
Space group	$P\bar{1}$	$P2_1/c$
a/Å	10.4550(8)	11.9533(5)
b/Å	12.6243(9)	11.6695(4)
c/Å	12.8445(10)	14.8764(5)
α/°	104.230(2)	90
β/°	90.169(2)	92.1240(10)
γ/°	109.486(2)	90
Volume/Å^3	1542.5(2)	2073.67(13)
Z	2	4
ρ_{calc}g/cm^3	3.092	3.298
μ/mm^{-1}	27.937	19.595
F(000)	1264.0	1824.0
Crystal size/mm	0.35x0.083x0.15	0.097x0.072x0.044
Diffractometer	STOE StadiVari	Bruker D8 Quest
Radiation	CuKα (λ = 1.5406)	MoKα (λ = 0.71073)
2Θ range for data collection/°	4.15 to 50.054	4.438 to 57.396
Reflections collected	74508	56068
Independent reflections	5429[R_{int} = 0.0712]	5361 [R_{int} = 0.0389]
Data/restraint/parameters	5429/6/254	5361/0/235
Goodness-of-fit on F^2	1.104	1.121
Final R indexes [all data]	R_1 = 0.0435, wR_2 = 0.1126	R_1 = 0.0193, wR_2 = 0.0300
Final R indexes [I≥2σ(I)]	R_1 = 0.0393, wR_2 = 0.1067	R_1 = 0.0173, wR_2 = 0.0296
Largest diff. peak/hole/ e Å^{-3}	4.20/–2.13	0.59/–0.96
CCDC	2505662	2505664

Compound	**7**	**5**
Empirical formula	$C_{41}H_{76}BiF_6P_2PtSb$	$C_6H_{10}BiF_6Sb$
Formula weight	1270.77	526.87
Crystal color, habit	red, block	yellow, block
Temperature/K	100	100
Crystal system	monoclinic	triclinic
Space group	$P2_1/c$	$P\bar{1}$
a/Å	14.1508(5)	7.5895(15)
b/Å	17.5667(7)	8.4740(16)
c/Å	19.2198(9)	10.0889(19)
α/°	90	81.580(7)
β/°	111.1810(10)	72.631(6)
γ/°	90	63.806(7)
Volume/Å^3	4454.9(3)	555.59(19)
Z	4	2
ρ_{calc}g/cm^3	1.895	3.149
μ/mm^{-1}	7.800	18.288
F(000)	2472.0	468.0
Crystal size/mm	0.072x0.063x0.045	0.061x0.038x0.033
Diffractometer	Bruker D8 Quest	Bruker D8 Quest
Radiation	MoKα (λ = 0.71073)	MoKα (λ = 0.71073)
2Θ range for data collection/°	3.86 to 61.106	4.23 to 50.6
Reflections collected	122869	14520
Independent reflections	13619 [R_{int} = 0.0642]	3715 [R_{int} = 0.0959]
Data/restraint/parameters	13619/0/560	3715/18/128
Goodness-of-fit on F^2	1.113	1.139
Final R indexes [all data]	R_1 = 0.0630, wR_2 = 0.0845	R_1 = 0.0889, wR_2 = 0.1775
Final R indexes [I≥2σ(I)]	R_1 = 0.0477, wR_2 = 0.0804	R_1 = 0.0629, wR_2 = 0.1520
Largest diff. peak/hole/ e Å^{-3}	1.61/–2.47	2.16/–2.03
CCDC	2505667	2505665

Compound	**8**	**6**
Empirical formula	$C_{42}H_{76}BiF_6P_2PtSb$	$C_6H_{14}BiF_6Sb$
Formula weight	1282.78	530.90
Crystal color, habit	red, plate	red, block
Temperature/K	100	100
Crystal system	monoclinic	monoclinic
Space group	*C2/c*	$P2_1/n$
a/Å	48.263(2)	8.2473(4)
b/Å	10.0613(4)	18.0235(8)
c/Å	18.7842(9)	8.7379(4)
α/°	90	90
β/°	93.7120(10)	112.6870(10)
γ/°	90	90
Volume/$Å^3$	9102.3(7)	1198.35(10))
Z	8	4
ρ_{calc}g/cm^3	1.872	2.943
μ/mm^{-1}	7.636	16.959
F(000)	4992.0	952.0
Crystal size/mm	0.031x0.130x0.180	0.154x0.115x0.105
Diffractometer	Bruker D8 Quest	Bruker D8 Quest
Radiation	MoKα (λ = 0.71073)	MoKα (λ = 0.71073)
2Θ range for data collection/°	4.136 to 60.23	4.52 to 54.254
Reflections collected	101820	21160
Independent reflections	13367 [R_{int} = 0.0870]	2651 [R_{int} = 0.0251]
Data/restraint/parameters	13367/12/527	2651/0/132
Goodness-of-fit on F^2	1.136	1.227
Final R indexes [all data]	R_1 = 0.0912, wR_2 = 0.1441	R_1 = 0.0154, wR_2 = 0.0317
Final R indexes [I≥2σ(I)]	R_1 = 0.0670, wR_2 = 0.1348	R_1 = 0.0149, wR_2 = 0.0316
Largest diff. peak/hole/ e $Å^{-3}$	5.57/–3.23	0.86/–0.78
CCDC	Not discussed	2505666

Compound	**9**	**[Bi(*c*Pr)Cl$_2$]**
Empirical formula	$C_{45}H_{86}BiCl_6F_6P_2PtSb$	$C_3H_5BiCl_2$
Formula weight	1541.59	320.95
Crystal color, habit	green, needle	colorless, plate
Temperature/K	100	100
Crystal system	monoclinic	monoclinic
Space group	*P2$_1$/c*	*P2$_1$/n*
a/Å	9.6417(4)	7.2182(4)
b/Å	20.3223(9)	11.2020(5)
c/Å	28.7101(12)	8.2419(4)
α/°	90	90
β/°	96.828(2)	114.083(2)
γ/°	90	90
Volume/Å^3	5585.6(4)	608.42(5)
Z	4	4
ρ_{calc}g/cm^3	1.833	3.504
μ/mm^{-1}	6.516	29.712
F(000)	3016.0	560.0
Crystal size/mm	0.38x0.059x0.056	0.13x0.017x0.032
Diffractometer	Bruker D8 Quest	Bruker D8 Quest
Radiation	MoKα (λ = 0.71073)	MoKα (λ = 0.71073)
2Θ range for data collection/°	4.254 to 52.744	6.342 to 55.72
Reflections collected	147512	8012
Independent reflections	11389 [R_{int} = 0.0551]	1454 [R_{int} = 0.0329]
Data/restraint/parameters	11389/6/563	1454/0/55
Goodness-of-fit on F^2	1.248	1.095
Final R indexes [all data]	R_1 = 0.0559, wR_2 = 0.1134	R_1 = 0.0167, wR_2 = 0.0356
Final R indexes [I≥2σ(I)]	R_1 = 0.0530, wR_2 = 0.1123	R_1 = 0.0156, wR_2 = 0.0353
Largest diff. peak/hole/ e Å^{-3}	3.23/–2.31	0.76/–0.54
CCDC	2505668	2505663

Compound	**[Pt(PCy$_3$)$_2$(η3-C$_3$H$_5$)(SbF$_6$)]**
Empirical formula	$C_{45}H_{76}F_6P_2PtSb$
Formula weight	1109.83
Crystal color, habit	orange, block
Temperature/K	100
Crystal system	monoclinic
Space group	*P2₁/n*
a/Å	19.1292(6)
b/Å	11.8695(4)
c/Å	21.5929(8)
α/°	90
β/°	109.5770(10)
γ/°	90
Volume/Å^3	4619.3(3)
Z	4
ρ_{calc}g/cm^3	1.596
μ/mm^{-1}	3.733
F(000)	2236.0
Crystal size/mm	0.403x0.241x0.166
Diffractometer	Bruker D8 Quest
Radiation	MoKα (λ = 0.71073)
2*Θ* range for data collection/°	3.972 to 61.998
Reflections collected	116269
Independent reflections	14722 [R_{int} = 0.0483]
Data/restraint/parameters	14722/0/506
Goodness-of-fit on F^2	1.039
Final R indexes [all data]	R_1 = 0.0352, wR_2 = 0.0559
Final R indexes [I≥2σ(I)]	R_1 = 0.0259, wR_2 = 0.0537
Largest diff. peak/hole/ e Å^{-3}	1.60/–1.07
CCDC	2505669

10. DFT calculations

All geometry optimizations and frequency analyses were performed using the Gaussian16[93] suite of programs. All compounds were analyzed using geometry optimizations and frequency analyses on the B3LYP+GD3/def2-TZVP level of theory.[94-99] Natural bond orbital analyses were performed with the NBO 6 program suite.[100] The enthalpies and free enthalpies are provided in Table S1. The cartesian coordinates of all geometry-optimized structures are provided in Table S2 (values in Å).

Table S1. Enthalpies, free enthalpies, HOMO- and LUMO-energies determined by DFT calculations.

Compound	*ΔH* [hartree]	*ΔG* [hartree]	HOMO [eV]	LUMO [eV]
$[Bi(CH_2)_5]^+$	-410.919264	-410.960834	-11.8940968	-9.23091847
$[Bi(cPr)_2]^+$	-448.970360	-449.017288	-11.8845728	-8.54900114
$[Bi(iPr)_2]^+$	-451.410509	-451.463674	-11.3659238	-8.75172597
$[BiMe_2]^+$	-294.203052	-294.241121	-12.7545208	-9.63010951
$[Pt(PCy_3)_2(Bi(CH_2)_5)]$ (**7**$^+$)	-2624.59933	-2624.73785	-8.60451237	-5.32962206
$[Pt(PCy_3)_2(Bi(cPr)_2)]$ (**8**$^+$)	-2662.63885	-2662.78423	-8.65458132	-5.27792043
$[Pt(PCy_3)_2(Bi(iPr)_2)]$ (**9**$^+$)	-2665.08834	-2665.23420	-8.33620810	-5.31356734
$[Pt(PCy_3)_2(BiMe_2)]$ (**I**$^+$)	-2507.88852	-2508.02345	-8.77730467	-5.39492939
$Pt(PCy_3)_2$	-2213.543876	-2213.664544	-4.67382766	-0.03864017

In each case, the LUMO of the $[BiR_2]^+$ ($R_2 = (CH_2)_5$, $(cPr)_2$, $(iPr)_2$, Me_2) complex cation shows dominant contributions by one p(Bi) orbital, which is orthogonal to the plane defined by the Bi atom and the two carbon atoms bound to it. The HOMOs and LUMOs of compounds **7**$^+$, **8**$^+$, **9**$^+$, and **I**$^+$ are qualitatively identical and shown for compound 7$^+$ in the figure below.

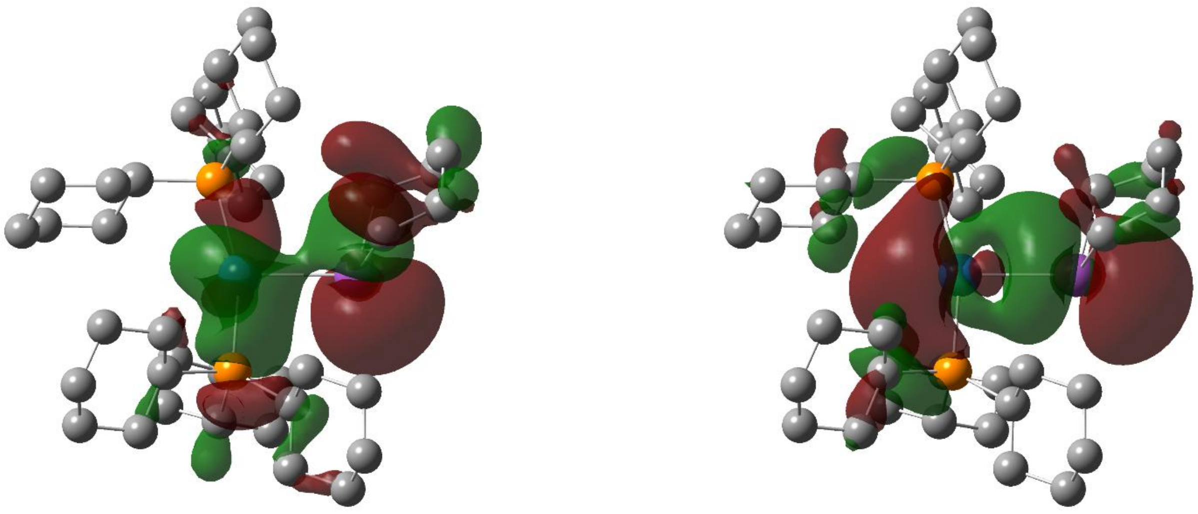

Figure S60: HOMO (left) and LUMO (right) of complex cation **7**$^+$ (isovalue = 0.02), as determined by DFT calculations.

According to NBO analyses, the Pt-Bi bonds are formed through Pt atomic orbitals (**7**$^+$: 69.8%, **8**$^+$: 70.1%, **9**$^+$: 70.8%) and Bi atomic orbitals (**7**$^+$: 30.2%, **8**$^+$: 29.9%, **9**$^+$: 29.2%), indicating a considerable polarization towards Pt. The Pt atoms contribute s-orbitals (**7**$^+$: 43%, **8**$^+$: 42%, **9**$^+$: 43%), and d-orbitals (**7**$^+$: 57%, **8**$^+$: 57%, **9**$^+$: 57%), while the Bi atoms contribute almost exclusively p-orbitals (**7**$^+$: 97%, **8**$^+$: 97%, **9**$^+$: 97%).

The Pt-Bi bonds are associated with Wiberg bond indices of 0.71 (**7**$^+$), 0.70 (**8**$^+$), and 0.70 (**9**$^+$).

Table S2. Cartesian coordinates (Å) of geometry-optimized structures obtained from DFT calculations.

$[Bi(CH_2)_5]^+$

Bi	0.82801362	-0.00012339	-0.01971596
C	-2.12412112	-1.29333475	-0.28011815
H	-2.76028035	-2.13497843	0.00852829
H	-2.13900894	-1.27371856	-1.37374617
C	-0.71299972	1.62227360	0.21675852
H	-0.70248988	1.76063652	1.30896178
H	-0.29669007	2.52998190	-0.22011303
C	-2.12235521	1.29330988	-0.28185479
H	-2.13483126	1.27119414	-1.37549376
H	-2.75888620	2.13589030	0.00324756
C	-2.73544159	0.00074086	0.26655271
H	-3.79757587	0.00120442	0.01645149
H	-2.68491154	0.00148296	1.36118680
C	-0.71363549	-1.62170671	0.21547077
H	-0.29836679	-2.52984044	-0.22142486
H	-0.70077053	-1.75930883	1.30797206

$[Bi(c\text{Pr})_2]^+$

Bi	0.02311900	-0.67724900	0.00004800
C	-1.95564500	0.24257900	-0.00045700
H	-2.75069700	-0.49240700	-0.00012800
C	1.17167000	1.17645500	-0.00069100
H	0.59045000	2.08899200	-0.00170400
C	2.52589100	1.25143300	0.73630900
H	2.69464300	2.18046200	1.26511100
H	2.89079200	0.37408800	1.25489700
C	-2.26624000	1.56047300	0.73649900
H	-1.46127700	2.06274000	1.25672900
H	-3.21061700	1.56623700	1.26487200
C	2.52680600	1.25022900	-0.73601600
H	2.89220000	0.37189600	-1.25259000
H	2.69640700	2.17825800	-1.26629800
C	-2.26690500	1.56077700	-0.73633400
H	-1.46235900	2.06312100	-1.25711700
H	-3.21189600	1.56663000	-1.26360800

$[Bi(\textit{i}Pr)_2]^+$

Bi	-0.00000012	-0.55380834	-0.00068001
C	-1.66295291	0.99040286	0.08279898
H	-1.19478185	1.91685775	0.41619517
C	1.66293920	0.99060922	-0.08024952
H	1.19463584	1.91802307	-0.41077098
C	1.99127618	1.06752436	1.41949793
H	1.14031531	1.35663903	2.04556238
H	2.75224931	1.84090775	1.57317387
H	2.42322991	0.13918542	1.80662922
C	-1.99110288	1.07186199	-1.41674659
H	-2.42226134	0.14444079	-1.80698696
H	-1.14024200	1.36366046	-2.04164905
H	-2.75274708	1.84505804	-1.56802196
C	2.85650623	0.62364952	-0.94716873
H	3.30162793	-0.32973854	-0.65450492
H	3.63127778	1.38827191	-0.83596085
H	2.60157067	0.57303556	-2.00616263
C	-2.85664536	0.62076311	0.94843513
H	-3.63140860	1.38571070	0.83944776
H	-2.60186600	0.56690322	2.00730541
H	-3.30171256	-0.33172899	0.65278106

$[BiMe_2]^+$

Bi	-0.00000000	-0.00000000	0.28645119
C	0.00000000	-1.63068337	-1.23727637
H	1.02625454	-1.69131811	-1.61636188
H	-0.28334832	-2.57799026	-0.78603480
H	-0.65865214	-1.35748693	-2.06166933
C	-0.00000000	1.63068337	-1.23727637
H	-1.02625454	1.69131811	-1.61636188
H	0.28334832	2.57799026	-0.78603480
H	0.65865214	1.35748693	-2.06166933

[Pt(PCy$_3$)$_2$Bi(CH$_2$)$_5$]$^+$ (**7**$^+$)

Bi	-0.02929966	2.40591020	-0.48631709
Pt	0.13221774	-0.29717133	-0.12593495
P	-2.07219955	-0.91434045	0.27758438
P	2.44362737	-0.41811248	-0.43229517
C	4.97476879	1.01740712	-0.26240394
H	5.47615144	0.26055297	0.34578026
H	5.14701016	0.75154456	-1.30587190
C	3.47395052	1.03784908	0.07051932
H	3.02507197	1.83359610	-0.53799782
C	5.59533957	2.39259622	0.02041051
H	5.16392503	3.12401745	-0.67222817
H	6.66658705	2.36061947	-0.18950921
C	-2.83857226	-0.13896735	1.77901014
H	-3.04085577	0.87810513	1.42292595
C	-1.85909493	-0.00022152	2.95934909
H	-1.62532803	-0.98239481	3.37133618
H	-0.91194306	0.41758318	2.61612566
C	-3.62387462	0.07042886	-3.51843157
H	-3.11203959	0.22465138	-4.47139556
H	-4.05214113	1.03795601	-3.23281260
C	-1.32533851	-3.20676317	1.81492435
H	-0.31170249	-2.79697362	1.85158348
H	-1.85549027	-2.80919524	2.67818160
C	3.86027203	2.81685564	1.81404053
H	3.32374942	3.56486936	1.21882457
H	3.71212479	3.08541441	2.86253272
C	-1.26619703	-4.73429851	1.93188707
H	-0.73476100	-5.00486785	2.84722795
H	-2.28512341	-5.12217447	2.03776489
C	5.34935825	2.85067155	1.45994443
H	5.89274036	2.19089371	2.14537926
H	5.75269095	3.85425812	1.60933736
C	-4.40221056	-1.68149716	-1.27292807
H	-3.95477667	-2.62697692	-1.59075700
H	-4.91260293	-1.87896624	-0.33028930

C	-0.60691047	-5.38567176	0.71670139
H	0.44908793	-5.09409816	0.67440029
H	-0.62588069	-6.47301136	0.81296536
C	3.26524839	1.43294394	1.54261045
H	3.76856622	0.70669974	2.18411044
H	2.20712052	1.40042805	1.80849338
C	-2.61052074	-0.35789376	-2.45426480
H	-1.83645599	0.40082648	-2.34654970
H	-2.09869626	-1.26590765	-2.78528706
C	-2.00985999	-2.76454110	0.51128766
H	-3.04864469	-3.10516821	0.54363238
C	-5.42104457	-1.24083159	-2.33058975
H	-6.18569325	-2.01252419	-2.44356066
H	-5.93562398	-0.33908130	-1.98064701
C	-4.74780121	-0.95502350	-3.67362580
H	-5.48404513	-0.60464124	-4.39982314
H	-4.33270932	-1.88705609	-4.07352585
C	-4.77902306	0.09708454	3.38271370
H	-5.05438429	1.08298503	2.99156384
H	-5.70409507	-0.37013512	3.72728410
C	-2.58041413	4.26552517	-0.24745230
H	-3.62764515	4.48167004	-0.48611842
H	-1.99845884	5.00655923	-0.80851254
C	-0.19125337	3.15910913	1.67024828
H	-0.73601205	2.40837417	2.23767931
H	0.82032044	3.22298919	2.06803587
C	-1.31448367	-3.42471726	-0.69527371
H	-1.78276467	-3.12596634	-1.63271952
H	-0.27912556	-3.06845789	-0.74469682
C	-3.29945989	-0.61908061	-1.10156126
H	-3.78961612	0.31319476	-0.80109379
C	2.50633490	-1.88051457	2.02463296
H	1.50133869	-1.47228923	2.15116399
H	3.18333357	-1.19795202	2.54091658
C	-0.90617857	4.51051084	1.72148597
H	-0.35499688	5.25987666	1.14106256

H	-0.87836194	4.86847157	2.75672940
C	4.43500035	-3.87741454	1.02816383
H	5.46024586	-4.24043645	0.92889192
H	3.79932110	-4.58596597	0.48501056
C	2.61599715	-3.26353167	2.67275810
H	2.35836052	-3.19515298	3.73219771
H	1.88278453	-3.93649526	2.21517232
C	-2.36709670	4.49342975	1.25460585
H	-2.82177101	5.44993616	1.52302637
H	-2.91502678	3.72727602	1.81603633
C	-4.17695801	-0.73538056	2.24291092
H	-4.02197171	-1.76380021	2.58379512
H	-4.88750623	-0.77862632	1.41717378
C	2.88297983	-1.95293348	0.53235892
H	2.20529386	-2.68872523	0.07981831
C	-2.47199409	0.85893485	4.06849328
H	-2.63660068	1.87553153	3.69331814
H	-1.76932908	0.94308427	4.90061520
C	-3.80217072	0.27247740	4.54641327
H	-4.24420683	0.90913188	5.31553181
H	-3.61750529	-0.70087945	5.01460967
C	-1.29643772	-4.94897900	-0.57530574
H	-2.32486881	-5.32596210	-0.58884997
H	-0.79408668	-5.37902318	-1.44487583
C	-2.26161438	2.85018469	-0.73354057
H	-2.51130244	2.71362768	-1.78518764
H	-2.81643243	2.11626955	-0.15387921
C	0.88187138	-0.83478948	-4.29878167
H	-0.15008014	-0.72131749	-4.63898633
H	1.47573168	-1.10108027	-5.17995461
C	3.00533818	-0.80737877	-2.16732726
H	4.07974531	-0.96380700	-2.05138521
C	4.31249655	-2.49632291	0.37163359
H	4.59616929	-2.56609599	-0.67912649
H	5.01618630	-1.80732416	0.84582598
C	2.81357887	0.35551306	-3.16523165

H	3.13805197	1.30381961	-2.73251769
H	3.49091523	0.16433224	-4.00421281
C	0.97860337	-1.95230264	-3.26203664
H	0.30837092	-1.71415233	-2.43009353
H	0.63633233	-2.90155036	-3.68094203
C	1.39022806	0.48308060	-3.71544223
H	1.36699608	1.27242083	-4.47005383
H	0.71166549	0.79260985	-2.91742926
C	2.40907742	-2.10822668	-2.74398615
H	3.04958364	-2.40546881	-3.58119833
H	2.46947310	-2.92494376	-2.02297863
C	4.01996849	-3.84765164	2.50065292
H	4.06796798	-4.85212720	2.92620141
H	4.73370186	-3.23580818	3.06327131

$[Pt(PCy_3)_2Bi(c\text{Pr})_2]^+$ (**8**$^+$)

Pt	-0.10535658	0.27224559	0.13110456
Bi	-0.05127358	-2.39883324	0.55752551
P	-2.38435348	0.34264207	-0.31742354
P	2.12580243	1.00501683	0.14038063
C	-3.52276729	-0.90773520	0.43430969
H	-3.06217611	-1.84414456	0.09557290
C	-2.61923387	0.18711850	-2.15199912
H	-3.68211039	0.34038002	-2.36045142
C	2.39506472	1.61156881	-1.59723819
H	1.55929644	2.31297284	-1.70847350
C	-1.81840881	1.26360129	-2.90936873
H	-2.14967519	2.26320286	-2.62266720
H	-0.76348589	1.18653834	-2.62676781
C	2.18038839	0.53164662	-2.67422558
H	1.25007893	-0.00676652	-2.48506033
H	2.98577829	-0.20442239	-2.63350739
C	1.79365124	2.16173580	2.65470263
H	0.83362735	1.63716124	2.70872450
H	2.54682622	1.46221757	3.02185204
C	-2.36386358	-1.36234053	-4.15178924

H	-3.41784073	-1.26638275	-4.43414005
H	-2.04964925	-2.36452165	-4.45219635
C	1.03262632	-2.97114567	-1.33716296
H	1.65612534	-2.18115233	-1.73101516
C	-4.97925876	-0.92696634	-0.05788076
H	-5.49515330	-0.02667749	0.28319700
H	-5.02666240	-0.92617723	-1.14799441
C	-1.54277545	-0.30008451	-4.88226297
H	-0.47883486	-0.45782631	-4.67199485
H	-1.66729201	-0.39810516	-5.96255043
C	-2.72760594	2.45251073	1.57261966
H	-1.75982067	2.11695704	1.95210963
H	-3.48878232	1.93618616	2.16053246
C	2.06680027	2.54117617	1.18653032
H	3.04821642	3.01909251	1.12569086
C	3.68855685	2.39830930	-1.85887018
H	3.83186911	3.17667570	-1.10653566
H	4.54563251	1.72395123	-1.78398987
C	-2.22687723	-1.22089015	-2.63372234
H	-2.83534114	-1.98100490	-2.13887976
H	-1.18690755	-1.41383728	-2.35652347
C	-3.47248786	-0.92625143	1.97142767
H	-3.96702584	-0.03464781	2.36230561
H	-2.43922173	-0.88977245	2.32531818
C	-2.89187975	3.96231482	1.77170200
H	-2.79621957	4.20740905	2.83176320
H	-2.07837215	4.48347345	1.25568667
C	1.02093901	3.55602151	0.69138073
H	1.23779700	3.86997697	-0.33079278
H	0.03177506	3.08241300	0.67328350
C	4.08078392	-1.02486690	-0.20730196
H	3.24335767	-1.65361910	-0.50458560
H	4.47309009	-0.56643715	-1.11842011
C	-1.94391454	1.10297187	-4.42741059
H	-2.97950392	1.29830077	-4.72629132
H	-1.32903167	1.85734921	-4.92388474

C	2.15375422	1.16422611	-4.06869822
H	2.02402621	0.38620346	-4.82465445
H	1.28120533	1.82295541	-4.14640203
C	3.42581681	1.96962039	-4.34320877
H	3.36927040	2.44467627	-5.32477696
H	4.28232904	1.28698022	-4.37569077
C	3.61779328	0.07856002	0.75952426
H	3.22696794	-0.41365049	1.65261121
C	-4.18922697	-2.16582364	2.51481295
H	-3.64907149	-3.06312819	2.19224518
H	-4.16278787	-2.15918615	3.60676964
C	1.75008665	3.39943933	3.55413562
H	2.73886758	3.87083058	3.56931328
H	1.53208480	3.09889949	4.58140476
C	-4.25643057	2.54747019	-0.43904496
H	-5.04730617	2.02306255	0.10200820
H	-4.37819788	2.30094470	-1.49486654
C	0.96720920	4.78772163	1.60109476
H	1.91946302	5.32411961	1.52693567
H	0.19656584	5.47431552	1.24315843
C	-2.88601632	2.08674647	0.08607576
H	-2.12413044	2.65928213	-0.45822462
C	-4.23562956	4.45470253	1.22985377
H	-5.04445873	4.02017048	1.82763991
H	-4.31415306	5.53810434	1.34102093
C	-5.70630957	-2.16189880	0.48975250
H	-6.74658854	-2.15198250	0.15738502
H	-5.25053310	-3.06219792	0.06240892
C	0.71258468	4.40782053	3.06002041
H	0.71928042	5.30064023	3.68850173
H	-0.28665773	3.96901568	3.14982607
C	1.67928347	-2.60783137	1.97711548
H	2.62419799	-2.83771215	1.50432548
C	4.81918418	0.94346292	1.19026341
H	5.22610588	1.47427138	0.32676935
H	4.51938721	1.70146365	1.91303298

C	1.72939881	-1.84200581	3.27593407
H	0.93865651	-1.13403897	3.49422933
H	2.70062598	-1.53313332	3.64361894
C	5.18417354	-1.88625142	0.41571533
H	5.50236413	-2.64584566	-0.30198229
H	4.78182357	-2.42309100	1.28162279
C	3.66385917	3.02322515	-3.25907429
H	4.60121752	3.55301448	-3.44219465
H	2.86691322	3.77425241	-3.30074089
C	6.37226496	-1.03499527	0.86229105
H	7.12913231	-1.65962229	1.34109293
H	6.84703257	-0.58643505	-0.01752673
C	1.51921375	-4.38224165	-1.55254043
H	2.47497692	-4.51744958	-2.04288142
H	1.30763372	-5.13651295	-0.80349587
C	-4.42518813	4.05796903	-0.23567905
H	-3.69059340	4.58585035	-0.85434672
H	-5.41077387	4.36765365	-0.58995857
C	1.40603437	-3.30325711	3.29631585
H	2.15616083	-3.99223999	3.66319092
H	0.39110127	-3.59754581	3.53897455
C	0.36638980	-3.85376995	-2.35929319
H	-0.61972992	-4.25347721	-2.15129512
H	0.53033283	-3.62808196	-3.40559369
C	-5.63526885	-2.23206351	2.01689769
H	-6.11749908	-3.14389108	2.37497981
H	-6.19868879	-1.39380558	2.44178128
C	5.91907550	0.07371203	1.81151665
H	6.76414897	0.70511512	2.09462325
H	5.53997948	-0.37383177	2.73739310

$[Pt(PCy_3)_2Bi(iPr)_2]^+$ (**9**$^+$)

Pt	-0.10747132	0.27688619	0.10879171
Bi	-0.07435114	-2.43082419	0.43876548
P	-2.39295081	0.37658217	-0.32067264
P	2.10780229	1.04938780	0.14967434

C	-3.52682014	-0.99787703	0.18853397
H	-2.97479658	-1.87872298	-0.16038876
C	-2.59922859	0.52847022	-2.15865526
H	-3.66354206	0.68604838	-2.35589710
C	2.45096016	1.60412849	-1.59251805
H	1.63360761	2.31733113	-1.75217325
C	-1.81973802	1.72456453	-2.73577929
H	-2.16140169	2.66132549	-2.29227071
H	-0.76016694	1.61743895	-2.47713684
C	2.25484934	0.49965876	-2.64714927
H	1.30010353	-0.00439930	-2.48453806
H	3.03474866	-0.25833293	-2.54763995
C	1.74897464	2.33202688	2.61791984
H	0.78170995	1.82932370	2.71933126
H	2.49720601	1.64463379	3.01478827
C	-2.31582150	-0.68934894	-4.36943789
H	-3.37788163	-0.59137381	-4.61873561
H	-1.97345614	-1.62179824	-4.82442383
C	1.29771004	-3.11682937	-1.31299555
H	1.79980304	-2.20425813	-1.63070529
C	-4.91739163	-1.05971213	-0.46705670
H	-5.52385624	-0.21299009	-0.14033332
H	-4.84317197	-0.99418967	-1.55322581
C	-1.54344779	0.49998508	-4.93982450
H	-0.47141028	0.33924361	-4.78417428
H	-1.69571918	0.57115147	-6.01867153
C	-2.66656308	2.21592617	1.85440860
H	-1.63007375	1.95502969	2.07849733
H	-3.28831217	1.54849701	2.45335797
C	1.99856419	2.62854810	1.12785945
H	2.96780002	3.12634700	1.03159869
C	3.76894928	2.36003069	-1.82359185
H	3.89533391	3.15917271	-1.09048812
H	4.60943056	1.67390770	-1.69210910
C	-2.15796946	-0.77238638	-2.84970640
H	-2.71446205	-1.62957950	-2.46556664

H	-1.10498427	-0.94353350	-2.60777690
C	-3.64283168	-1.12578577	1.71665212
H	-4.22715032	-0.28769922	2.10459973
H	-2.65745300	-1.06623630	2.18612680
C	-2.95514716	3.66474101	2.26092875
H	-2.76099030	3.79343032	3.32811754
H	-2.26262366	4.33008243	1.73356685
C	0.92142861	3.59304397	0.59954351
H	1.09622026	3.84193535	-0.44815343
H	-0.05949529	3.10490865	0.64833836
C	4.09422454	-0.95714405	-0.04146465
H	3.26791595	-1.58313357	-0.36804994
H	4.53499591	-0.52588993	-0.94362968
C	-1.96276720	1.80366910	-4.25948152
H	-3.00623539	2.02370199	-4.50980035
H	-1.36998355	2.64006576	-4.63752625
C	2.30887859	1.09002559	-4.05937999
H	2.19219263	0.29263447	-4.79705922
H	1.45932020	1.76884070	-4.19562458
C	3.61083519	1.85652804	-4.30049059
H	3.61092818	2.29839795	-5.29902449
H	4.45249418	1.15560070	-4.26886427
C	3.58862147	0.17482557	0.86659195
H	3.17318906	-0.28455658	1.76857042
C	-4.34307532	-2.43356316	2.09645551
H	-3.72044966	-3.27791859	1.78022456
H	-4.43563952	-2.50088075	3.18274550
C	1.74158891	3.62246934	3.44122164
H	2.73391038	4.08441427	3.39542668
H	1.55470371	3.38797168	4.49150398
C	-4.41814032	2.39116892	0.03592748
H	-5.09141153	1.72804919	0.58474111
H	-4.63515383	2.25653495	-1.02523348
C	0.88194705	4.88262754	1.42763496
H	1.82070149	5.42646371	1.27621673
H	0.08489205	5.53136283	1.05630701

C	-2.96307519	2.01073994	0.35760240
H	-2.31504319	2.71058101	-0.18346198
C	-4.39239624	4.06976789	1.92806901
H	-5.08414926	3.47812772	2.53787880
H	-4.56084681	5.11579609	2.19212446
C	-5.62413433	-2.36604519	-0.08206850
H	-6.61948948	-2.38861648	-0.53104732
H	-5.07003851	-3.21004545	-0.50813009
C	0.69266505	4.60446089	2.91985937
H	0.73181394	5.53898780	3.48324663
H	-0.30230499	4.18149558	3.08576136
C	1.44625919	-2.56021230	2.17201555
H	2.41266949	-2.31375218	1.73702975
C	4.77232942	1.05795760	1.30832477
H	5.22011810	1.54247326	0.43833894
H	4.44723424	1.85271362	1.97847542
C	1.08659533	-1.56460629	3.26784427
H	1.05755268	-0.53765628	2.90428079
H	1.81680564	-1.61296742	4.08375578
H	0.10876652	-1.78547686	3.70466384
C	5.15580215	-1.80511910	0.66473745
H	5.50592692	-2.58954668	-0.01007820
H	4.70338580	-2.31113111	1.52487519
C	3.81868486	2.94104877	-3.24159965
H	4.77309127	3.44874949	-3.39716167
H	3.03831911	3.70356425	-3.34378851
C	6.32536721	-0.94357970	1.14180579
H	7.04899736	-1.55307032	1.68679240
H	6.85143491	-0.53700354	0.27088443
C	2.31862453	-4.15646513	-0.86392591
H	1.83474859	-5.07498779	-0.52174716
H	2.96829421	-3.80346937	-0.06326485
H	2.96233135	-4.43670728	-1.70521370
C	-4.70304393	3.83957811	0.44821243
H	-4.09181014	4.51354894	-0.16251963
H	-5.74580064	4.08359882	0.23384660

C	1.46710988	-3.99658897	2.69052280
H	0.49151201	-4.30159200	3.08005217
H	2.17878508	-4.08746590	3.51864606
H	1.76653105	-4.71853142	1.92960773
C	0.42721345	-3.64161713	-2.45103841
H	1.05485527	-3.95017219	-3.29437262
H	-0.27366494	-2.89526803	-2.82466069
H	-0.15011629	-4.51996722	-2.14943121
C	-5.71952138	-2.53621666	1.43555280
H	-6.18613020	-3.49287473	1.67911116
H	-6.37275059	-1.75735822	1.84474721
C	5.83565811	0.20983967	2.01820376
H	6.67223194	0.84774034	2.31162057
H	5.41011954	-0.19273508	2.94440165

$[Pt(PCy_3)_2BiMe_2]^+$ (**I**$^+$)

Bi	-0.31464917	-2.62940212	0.09779172
Pt	-0.02766726	0.07632644	-0.09852547
P	2.24862930	0.36958738	0.25954025
P	-2.28509002	0.52478700	-0.52267808
C	-5.02936654	-0.41401310	-0.23398279
H	-5.40450651	0.51575870	0.20059964
H	-5.13030410	-0.32210922	-1.31581616
C	-3.56233709	-0.62211774	0.17719041
H	-3.24510679	-1.58040275	-0.25415905
C	-5.88581078	-1.58931186	0.25742974
H	-5.57314820	-2.49846206	-0.26844943
H	-6.93011256	-1.41836626	-0.01243298
C	2.88881642	-0.24894178	1.88968361
H	2.97006510	-1.32732023	1.70807075
C	1.87608920	-0.06641568	3.03689871
H	1.74023132	0.99454994	3.25020782
H	0.89865266	-0.44395944	2.73887563
C	3.56951985	-1.42346443	-3.32254567
H	3.03883027	-1.60171108	-4.26093092
H	3.78906069	-2.40850442	-2.89529836

C	1.87984281	2.97608313	1.36972535
H	0.80868500	2.75900226	1.41879282
H	2.31048136	2.63774648	2.31007557
C	-4.29154637	-1.95630276	2.18883837
H	-3.87976580	-2.88090506	1.76811652
H	-4.21522463	-2.04545091	3.27480688
C	2.09066044	4.48929946	1.24448343
H	1.59963879	4.99168533	2.08111048
H	3.16018138	4.70797727	1.33482052
C	-5.75541336	-1.80602790	1.76689589
H	-6.19076732	-0.94889490	2.29246340
H	-6.33057309	-2.68248067	2.07206006
C	4.68525732	0.42930692	-1.31399173
H	4.43885712	1.39115285	-1.77179510
H	5.22449270	0.65154152	-0.39389918
C	1.57789986	5.03829549	-0.08635225
H	0.48783478	4.92999313	-0.12989493
H	1.78761491	6.10706540	-0.16151251
C	-3.45945433	-0.76606026	1.70513022
H	-3.84223681	0.14092075	2.17752559
H	-2.41809981	-0.86127830	2.01729430
C	2.66498883	-0.64961623	-2.36005876
H	1.74362103	-1.20184202	-2.18003568
H	2.36382230	0.29045891	-2.82929270
C	2.50088893	2.21728428	0.18501810
H	3.58280670	2.37723106	0.20957282
C	5.59503979	-0.35150665	-2.26992137
H	6.50118339	0.22665169	-2.46371982
H	5.91412902	-1.28010793	-1.78400305
C	4.88145031	-0.68132264	-3.58159750
H	5.53162758	-1.27369774	-4.22847805
H	4.66850435	0.24939935	-4.11944645
C	4.75477639	-0.41532546	3.59597108
H	4.91020904	-1.48335515	3.40678254
H	5.72542541	-0.00610804	3.88443882
C	-0.19901758	-2.92715481	2.35222826

H	0.82392663	-2.78939679	2.68980595
H	-0.86496713	-2.24040255	2.86741528
C	1.95330334	2.78097181	-1.14048592
H	2.38148801	2.26137933	-1.99669683
H	0.87285484	2.60168981	-1.18294898
C	3.38837883	-0.35413499	-1.03191292
H	3.67267073	-1.31700109	-0.59381477
C	-2.13120598	2.40008659	1.62934193
H	-1.21327696	1.86038409	1.87194618
H	-2.92306817	1.94530360	2.22673896
C	-3.67228233	4.47474216	0.20734534
H	-4.61808199	4.97922429	-0.00165162
H	-2.91602999	4.95634530	-0.42295574
C	-2.01108686	3.87665514	2.01683507
H	-1.78545761	3.96040181	3.08241972
H	-1.16584586	4.32309945	1.48197832
C	4.28316784	0.25514397	2.29932716
H	4.25366355	1.33940848	2.44306685
H	5.01029134	0.05997029	1.51149561
C	-2.46766912	2.26111523	0.13217103
H	-1.66655285	2.77818783	-0.41237344
C	2.36352898	-0.75990347	4.31180240
H	2.41721666	-1.84120754	4.14253662
H	1.63712153	-0.60648533	5.11302109
C	3.74268147	-0.24686485	4.72931328
H	4.08814174	-0.76899500	5.62385323
H	3.66797735	0.81363845	4.99514132
C	2.20073106	4.28535347	-1.26114680
H	3.27956905	4.47344375	-1.28769721
H	1.79678702	4.64792973	-2.20920210
C	1.78840130	-3.45564242	-0.17810832
H	2.14860456	-3.26925955	-1.18607750
H	2.46641451	-3.03178194	0.55774375
C	-0.60130223	-0.05602672	-4.30661610
H	0.40273220	-0.40239914	-4.56188947
H	-1.11854567	0.13264870	-5.25359632

C	-2.74169021	0.67563709	-2.32452970
H	-3.77418429	1.02915593	-2.29148556
C	-3.77964774	2.99630256	-0.18783447
H	-4.03127387	2.91913570	-1.24625150
H	-4.59834149	2.53233281	0.36829504
C	-2.73730342	-0.66821019	-3.08517473
H	-3.23570631	-1.44970520	-2.50855404
H	-3.34956074	-0.52408992	-3.98157380
C	-0.52613124	1.23940900	-3.50066636
H	0.07512403	1.05727229	-2.60428477
H	-0.01548174	2.02061434	-4.06863030
C	-1.34647254	-1.13598758	-3.52265813
H	-1.44439741	-2.04557755	-4.11929409
H	-0.75206757	-1.39925793	-2.64449944
C	-1.91806311	1.72673627	-3.09655512
H	-2.48024280	1.96317007	-4.00622232
H	-1.85068415	2.66309757	-2.54038051
C	-3.29073583	4.64518756	1.67934732
H	-3.17140319	5.70411026	1.91756672
H	-4.10760472	4.27397206	2.30800398
H	1.71282868	-4.53138614	-0.01278656
H	-0.51322697	-3.95340198	2.54570127

$Pt(PCy_3)_2$

P	2.30440312	-0.28826271	-0.02164169
P	-2.17482618	0.14415888	0.13502209
C	-3.04647828	0.05586487	1.77842860
H	-2.59807894	0.91055700	2.29825566
C	3.27726380	0.04886732	1.53560555
H	3.27845559	-0.92964582	2.02665223
C	-3.06102544	-0.90968648	-1.12318398
H	-2.38172201	-0.80843678	-1.97792598
C	2.85108990	1.02539160	-1.23890421
H	3.94629717	1.00793686	-1.27822074
C	-4.56911674	0.24506767	1.80133951
H	-4.86321972	1.13188155	1.23521014

H	-5.04633831	-0.61140509	1.31686873
C	-2.64218204	-1.18820902	2.58912745
H	-3.05254515	-2.08699923	2.12086183
H	-1.55596385	-1.29679024	2.57498221
C	2.39133425	2.41866185	-0.77485010
H	1.31108751	2.38470036	-0.60622862
H	2.84547801	2.66758936	0.18429513
C	3.08125965	-1.84633782	-0.73469956
H	3.90978273	-1.52183836	-1.37643806
C	4.73870164	0.47672139	1.33763637
H	5.25811999	-0.21071662	0.66486790
H	4.76855055	1.46021807	0.85879553
C	3.65183722	-2.82137713	0.30882822
H	4.42482266	-2.34357777	0.91160215
H	2.85202224	-3.12001698	0.99593728
C	-5.08706183	0.34550246	3.24114481
H	-4.67913862	1.25066808	3.70567925
H	-6.17496736	0.45680146	3.24041556
C	-1.88605666	2.91254455	0.52760306
H	-0.82814142	2.67059553	0.67003324
H	-2.35893333	2.83336549	1.50859435
C	-3.16188756	-1.09325343	4.02703152
H	-2.89344874	-1.99700833	4.58078380
H	-2.66379818	-0.25739180	4.53202689
C	-3.04087273	-2.39545718	-0.73078394
H	-2.03788974	-2.67649796	-0.40159192
H	-3.71420843	-2.55753481	0.11672166
C	-4.46171269	-0.49428202	-1.59880784
H	-4.47688855	0.55359484	-1.90356477
H	-5.17914557	-0.59185750	-0.78141150
C	2.04502152	-2.58842881	-1.60331659
H	1.60989045	-1.92207579	-2.34679034
H	1.21351379	-2.88744236	-0.95785200
C	2.64584021	-3.82261506	-2.27974062
H	1.88068639	-4.32534563	-2.87761743
H	3.43327246	-3.51117145	-2.97665943

C	3.29496330	1.07681770	3.84393692
H	2.78551101	1.77692241	4.51166858
H	3.25903556	0.09609138	4.33273932
C	-4.67699141	-0.87602467	4.06782074
H	-5.01959478	-0.76745628	5.10035752
H	-5.17707790	-1.76421039	3.66371894
C	5.47740104	0.55855234	2.67835556
H	5.53841719	-0.44490059	3.11538132
H	6.50599123	0.89412222	2.51931140
C	2.55179103	0.99760619	2.50661844
H	2.48332211	1.99991101	2.07702342
H	1.52482083	0.65696052	2.64936301
C	-2.49400629	1.88842905	-0.44783613
H	-3.57595872	2.05386615	-0.49269630
C	-2.02473197	4.34995954	0.01760933
H	-3.08623035	4.62232912	-0.02072234
H	-1.55153842	5.03638802	0.72515612
C	2.14857870	3.19787229	-3.16885922
H	2.41306337	3.98074581	-3.88467393
H	1.05449356	3.17817303	-3.11184383
C	2.65214773	1.83780182	-3.64985773
H	3.73874418	1.88626242	-3.78900603
H	2.22307314	1.59288123	-4.62543612
C	4.24614405	-4.07114494	-0.35235170
H	5.11258224	-3.77429196	-0.95507937
H	4.62084748	-4.75063447	0.41826853
C	2.31621652	0.72657015	-2.65089696
H	1.23034091	0.60079099	-2.59253240
H	2.72356423	-0.21646733	-3.01821158
C	3.23396154	-4.78580650	-1.24802260
H	2.42256737	-5.18320251	-0.62756718
H	3.70143288	-5.64232659	-1.74125567
C	2.71940941	3.51837237	-1.78827374
H	2.33193411	4.47487556	-1.42601551
H	3.80725368	3.63126619	-1.86689526
C	-1.90650065	2.08138063	-1.85886061

H	-0.84904173	1.80248054	-1.82916145
H	-2.38438843	1.40517657	-2.57042299
C	-2.04205659	3.52289324	-2.35573484
H	-1.57682441	3.61513406	-3.34125094
H	-3.10289990	3.76749208	-2.48629155
C	-4.91694777	-1.37564819	-2.76905358
H	-5.92350545	-1.08652026	-3.08383663
H	-4.25736037	-1.19859327	-3.62650770
C	4.75689743	1.49105407	3.65551306
H	4.79172433	2.51461097	3.26388940
H	5.27670208	1.50659105	4.61721297
C	-1.41534495	4.51360163	-1.37471468
H	-0.33631915	4.33172212	-1.31918013
H	-1.54418325	5.53896505	-1.73144502
C	-4.88015224	-2.86189150	-2.40389156
H	-5.17007592	-3.47103468	-3.26420208
H	-5.62132302	-3.05537850	-1.61932247
C	-3.49644965	-3.27704443	-1.89707442
H	-2.76994061	-3.19168558	-2.71312875
H	-3.50307412	-4.32702025	-1.59197976
Pt	0.05270524	-0.26787933	0.19710614

11. References

80. G. R. Fulmer, A. J. M. Miller, N. H. Sherden, *et al.*, “NMR Chemical Shifts of Trace Impurities: Common Laboratory Solvents, Organics, and Gases in Deuterated Solvents Relevant to the Organometallic Chemist,” *Organometallics* 29 (2010): 2176–2179, https://doi.org/10.1021/om100106e.

81. O. V. Dolomanov, L. J. Bourhis, R. J. Gildea, J. A. K. Howard, and H. Puschmann, “OLEX2: A Complete Structure Solution, Refinement and Analysis Program,” *Journal of Applied Crystallography* 42 (2009): 339–341, https://doi.org/10.1107/S0021889808042726.

82. G. M. Sheldrick, “SHELXT—Integrated Space-Group and Crystal-Structure Determination,” *Acta Crystallographica Section A Foundations and Advances* 71 (2015): 3–8, https://doi.org/10.1107/S2053273314026370.

83. G. M. Sheldrick, “A Short History of SHELX,” *Acta Crystallographica Section A Foundations of Crystallography* 64 (2008): 112–122, https://doi.org/10.1107/S0108767307043930.

84. T. Yoshida, S. Otsuka, D. G. Jones, *et al*., in *Inorganic Syntheses* (1990), 113–119.

85. H. Althaus, H. J. Breunig, and E. Lork, “Syntheses and Chemistry of Methylantimony and Methylbismuth Dihalides: An Extended Two-Dimensional Framework in the Crystal Structure of CH_3BiCl_2 and Molecular Units in the Structures of [CH_3ECl_2 (2,2’-bipyridine)] (E = Sb, Bi),” *Organometallics* 20 (2001): 586–589, https://doi.org/10.1021/om000749i.

86. T. Dunaj, M. Egorycheva, A. Arebi, K. Dollberg, and C. von Hänisch, *Zeitschrift für Anorganische und Allgemeine Chemie* (2023): 649.

87. J. D. Smith and J. D. Oliver, “Ligand Profiles of Tricyclohexylphosphine. Structure of (π-Allyl)Bis(Tricyclohexylphosphine)Platinum Hexafluorophosphate,” *Inorganic Chemistry* 17 (1978): 2585–2589, https://doi.org/10.1021/ic50187a045.

88. A. Knierzinger and P. Schönholzer, “Axially Dissymmetric Diphosphines in the Biphenyl Series: Crystal Structures of Three New Group-VIII Metal Complexes of (6,6′ -Dimethyl-1,1′ -Biphenyl-2,2′ -Diyl)Bis(Diphenylphosphine) (= Biphemp),” *Helvetica Chimica Acta* 75 (1992): 1211–1220, https://doi.org/10.1002/hlca.19920750421 .

89. T.-M. Huang, R.-H. Hsu, C.-S. Yang, J.-T. Chen, G.-H. Lee, and Y. Wang, “Organometallic Transformations From an η^3-Propargyl or η^2-Allenyl Ligand to η^3-Hydroxyallyl, η^3-Heterotrimethylenemethane, and η^6-Diallyl Ether Species,” *Organometallics* 13 (1994): 3657–3663, https://doi.org/10.1021/om00021a045.

90. B. Crociani, F. Benetollo, R. Bertani, G. Bombieri, F. Meneghetti, and L. Zanotto, “Convenient Synthesis of Cationic Allylplatinum(II) Complexes With Tertiary Phosphines by Oxidative Allyl Transfer From Ammonium Cations to Platinum(0) Substrates. Crystal and Molecular Structures of η^3-Propenyl- and η^3-2-Methylpropenyl- Bis(Triphenylphosphine)Platinum(II)Perchlorates,” *Journal of Organometallic Chemistry* 605 (2000): 28–38, https://doi.org/10.1016/S0022-328X(00)00251-5.

91. M. A. Ortuño, N. A. Jasim, A. C. Whitwood, A. Lledós, and R. N. Perutz, *Dalton Transactions* 45 (2016): 18842–18850.

92. N. W. Alcock, M. Ravindran, and G. R. Willey, “Crown Ether Complexes of Bi III. Synthesis and Crystal and Molecular Structures of $BiCl_3$·12-Crown-4 and 2 $BiCl_3$·18-Crown-6,” *Journal of the Chemical Society, Chemical Communications* (1989): 1063–1065, https://doi.org/10.1039/C39890001063.

93. M. J. Frisch, G. W. Trucks, H. B. Schlegel, *et al*. Gaussian 16, Revision A.03 (2016).

94. A. D. Becke, “Density-Functional Thermochemistry. III. The Role of Exact Exchange,” *Journal of Chemical Physics* 98 (1993): 5648–5652, https://doi.org/10.1063/1.464913 .

95. C. Lee, W. Yang, and R. G. Parr, “Development of the Colle-Salvetti Correlation-Energy Formula Into a Functional of the Electron Density,” *Physical Review B* 37 (1988): 785–789, https://doi.org/10.1103/PhysRevB.37.785.

96. S. H. Vosko, L. Wilk, and M. Nusair, “Accurate Spin-Dependent Electron Liquid Correlation Energies for Local Spin Density Calculations: A Critical Analysis,” *Canadian Journal of Physics* 58 (1980): 1200–1211, https://doi.org/10.1139/p80-159.

97. P. J. Stephens, F. J. Devlin, C. F. Chabalowski, and M. J. Frisch, “Ab Initio Calculation of Vibrational Absorption and Circular Dichroism Spectra Using Density Functional Force Fields,” *Journal of Physical Chemistry* 98 (1994): 11623–11627, https://doi.org/10.1021/j100096a001.

98. S. Grimme, J. Antony, S. Ehrlich, and H. Krieg, “A Consistent and Accurate Ab Initio Parametrization of Density Functional Dispersion Correction (DFT-D) for the 94 Elements H-Pu,” *Journal of Chemical Physics* 132 (2010): 154104, https://doi.org/10.1063/1.3382344.

99. F. Weigend and R. Ahlrichs, “Balanced Basis Sets of Split Valence, Triple Zeta Valence and Quadruple Zeta Valence Quality for H to Rn: Design and Assessment of Accuracy,” *Physical Chemistry Chemical Physics* 7 (2005): 3297–3305, https://doi.org/10.1039/b508541a.

100. E. D. Glendening, C. R. Landis, and F. Weinhold, “NBO 6.0: Natural Bond Orbital Analysis Program,” *Journal of Computational Chemistry* 34 (2013): 1429–1437, https://doi.org/10.1002/jcc.23266.
